# Truffle Extract Attenuates TGF-β1-Induced Fibrosis-Associated Responses in HFL-1 Cells and Is Associated with Changes in Smad and MAPK Signaling

**DOI:** 10.3390/ijms272110030

**Published:** 2026-11-09

**Authors:** Te-Kai Sun, Jyun-Yi Li, Yu-Wen Sun, Jing-Wen Huang, Yi-Ying Wu, Muhammad Munir, Hung-Jen Liu

**Affiliations:** 1 Doctor Program in Biotechnology Industrial Management and Innovation, National Chung Hsing University, Taichung 40227, Taiwan; kay_gm@tsairderbiotech.com (T.-K.S.); sunyuwun@gmail.com (Y.-W.S.); 2 Institute of Molecular Biology, National Chung Hsing University, Taichung 40227, Taiwan; alert109209@hotmail.com (J.-Y.L.); jwhuang85@gmail.com (J.-W.H.); yiying939@gmail.com (Y.-Y.W.); 3 iEGG and Animal Biotechnology Center, National Chung Hsing University, Taichung 40227, Taiwan; 4 Division of Biomedical and Life Sciences, Faculty of Health and Medicine, Lancaster University, Lancaster LA1 4YQ, UK; muhammad.munir@lancaster.ac.uk; 5 Rong Hsing Research Center for Translational Medicine, National Chung Hsing University, Taichung 40227, Taiwan; 6 Department of Life Sciences, National Chung Hsing University, Taichung 40227, Taiwan

**Keywords:** truffle, fibrosis, TGF-β/Smad signaling pathways, MAPK signaling pathways

## Abstract

Cellular fibrosis is a key pathological feature of chronic disease and is characterized by persistent fibroblast activation and excessive extracellular matrix deposition. In this study, an aqueous truffle extract (TE) was characterized by untargeted LC-ESI(+)-MS/MS and LC-ESI(−)-MS/MS profiling that yielded putative LC-MS/MS annotations, and was evaluated in a TGF-β1-induced HFL-1 fibroblast model. TE attenuated TGF-β1-induced Smad2 phosphorylation, Smad2/3-Smad4 association, nuclear accumulation of Smad2/3 and Smad4, and the expression of the fibrosis-associated proteins α-smooth muscle actin (α-SMA) and fibronectin. TGF-β1 increased MEK1/2 phosphorylation, whereas TE further increased p-MEK1/2 while decreasing Ras expression and p-ERK1/2, revealing a divergent, context-dependent MAPK response rather than uniform activation or inhibition of the Ras/MEK/ERK pathway. shRNA-mediated reduction in TGF-β1, MEK1/2, or ERK1/2 altered fibrosis-associated protein expression in the absence of TE and further modified the protein-expression patterns observed after TE treatment, indicating that the TE response occurred within a signaling context influenced by these components without establishing strict pathway dependence or a linear signaling hierarchy. In the DCFH-DA assay, TGF-β1 increased intracellular oxidation-dependent fluorescence, whereas both TGF-β1 plus TE and TGF-β1 plus N-acetyl-L-cysteine reduced the signal. LC-MS/MS profiling additionally provided exploratory compositional context for the tested aqueous preparation, while the database best matches and putative LC-MS/MS annotation names remain exploratory and unconfirmed. Collectively, these findings show that TE attenuates TGF-β1-induced fibrosis-associated responses in HFL-1 cells in association with suppression of canonical Smad signaling, reduced intracellular oxidative activity, and context-dependent remodeling of MAPK-network readouts. These coordinated changes suggest that the antifibrotic-associated activity of TE involves multiple interconnected cellular processes rather than modulation of a single signaling component. The results support further activity-guided chemical characterization and validation in additional fibroblast and in vivo fibrosis models.

## 1. Introduction

Fibrosis is a pathological process characterized by excessive accumulation of extracellular matrix components, primarily collagen, which disrupts normal tissue architecture and function [1,2]. It plays a central role in the progression of various chronic diseases affecting organs such as the liver, lungs, kidneys, and heart. Despite extensive research, effective therapeutic strategies to prevent or reverse fibrosis remain limited [3,4,5]. One of the key pathways implicated in fibrogenesis is the transforming growth factor-beta (TGF-β) signaling pathway. TGF-β acts as a potent profibrotic cytokine by activating canonical Smad-dependent and non-canonical pathways, including mitogen-activated protein kinases (MAPKs) such as ERK, JNK, and p38 [6,7,8]. These signaling cascades collectively promote the activation of fibroblasts into myofibroblasts, the main effector cells responsible for the extracellular matrix (ECM) production and tissue scarring [9]. Persistent activation of fibroblasts and their transition toward a myofibroblast-like phenotype are central features of fibrotic remodeling and are commonly accompanied by increased α-smooth muscle actin (α-SMA) expression and extracellular matrix production.

In canonical TGF-β signaling, receptor activation promotes phosphorylation of receptor-regulated Smad proteins, followed by association with Smad4 and nuclear accumulation of the resulting complexes, which participate in the regulation of fibrosis-associated gene expression [6,7,8,9]. In parallel, TGF-β can engage non-canonical signaling networks, including MAPK pathways [6,7,8]. Crosstalk between canonical Smad signaling and MAPK signaling can influence the magnitude and duration of fibroblast responses [10,11], making it important to interpret changes in individual signaling proteins together with downstream fibrosis-associated readouts. Recent research has increasingly focused on the potential of natural bioactive compounds to modulate fibrosis-associated signaling pathways, offering alternative therapeutic strategies. Natural products may provide chemically diverse candidates for further investigation [12,13]. Among them, truffles—hypogenous fungi belonging to the genus Tuber—are rich in bioactive compounds, including polysaccharides, polyphenols, and antioxidants [14,15,16]. Traditionally valued for their culinary and medicinal properties, recent studies have begun to reveal the potential pharmacological activities of truffle extracts, including anti-inflammatory, antioxidant, and immunomodulatory effects. However, their role in fibrotic disease and underlying mechanisms has not been thoroughly explored [17]. Oxidative imbalance is also closely associated with fibrotic remodeling and can interact with TGF-β-dependent signaling [18,19], providing an additional cellular context in which the effects of chemically complex natural preparations can be evaluated.

However, the effects of aqueous truffle preparations on fibroblast-associated fibrotic responses, Smad complex formation and subcellular distribution, MAPK-network behavior, and intracellular oxidative activity remain insufficiently characterized. Defining these responses together may provide a more integrated view of the cellular changes associated with TE while avoiding assumptions of a single linear mechanism. ERK-related signaling has been implicated in fibrosis-associated remodeling in corneal and skin tissues, illustrating the cross-tissue relevance and complexity of MAPK regulation [10,11]. Accordingly, changes in individual MAPK components should be interpreted in relation to downstream signaling and functional readouts rather than as evidence of uniform pathway activation or inhibition. The Ras/RAF/MEK/ERK cascade is subject to feedback and compensatory regulation, and changes in MEK1/2 phosphorylation do not necessarily produce parallel changes in ERK1/2 phosphorylation [20,21]. This signaling complexity is particularly relevant when evaluating multicomponent natural preparations, for which coordinated changes across several interconnected pathways may occur without establishing a single direct molecular target. Accordingly, this study used an HFL-1 in vitro fibrosis model to evaluate the fibrosis-associated cellular effects of truffle extract (TE) and their association with changes in TGF-β1/Smad and MAPK signaling.

## 2. Results

### 2.1. LC-MS/MS Profiling of the Aqueous Truffle Extract

After application of the revised screening criteria, the final LC-MS/MS datasets comprised 231 positive-ion features and 143 negative-ion features. Features that did not satisfy the revised acceptance criteria were excluded from the final Supplementary Tables S1 and S2 rather than retained as named or unnamed entries. Each retained entry is reported with chemical class, putative annotation, peak area, retention time, precursor-ion *m*/*z*, adduct type, calculated *m*/*z*, mass error (ppm), candidate formula, and matched-fragment count (Supplementary Tables S1 and S2). Experimental MS/MS spectra were acquired for the retained features during information-dependent acquisition, and matched-fragment counts are reported at the feature level. Supplementary Figure S1 presents two representative experimental MS/MS spectra as examples of the fragmentation data available for the retained annotations. Repeated annotations were re-evaluated using ionization mode, retention time, precursor *m*/*z*, exact-mass agreement, peak-area similarity, and available archived analytical information rather than peak area alone. Peak area was not used alone to retain or remove an annotation. The same annotation assigned to mode-specific features in the positive- and negative-ion datasets was treated as mode-specific evidence rather than as confirmation of a compound identity. Protein database searching yielded 133 protein-family groups; after score-based filtering, Supplementary Table S3 reports 84 exploratory database best-match entries with at least one matched peptide having a Mascot Ions Score > 43 (*p* < 0.05 according to the archived Mascot search report). Datasheet 1 contains 220 single-entry peptide rows and 41 shared peptide rows. Because authentic standards were not analyzed, all retained small-molecule names are reported as putative candidate annotations rather than confirmed molecular identities. The major chemical classes represented by the putatively annotated LC-MS/MS features, together with representative candidate annotations and their literature-reported biological activities, are summarized in Table 1.

**Table 1:** Major chemical classes represented by putatively annotated LC-ESI(+)-MS/MS and LC-ESI(−)-MS/MS features in the aqueous truffle extract and their literature-reported context.

Chemical Class	Representative Putative LC-MS/MS Annotations	Literature-Reported Context
Phenolic compounds	trans-Ferulic acid, Sinapic acid, Gallic acid, Salicylic acid, Paeonol, 2-Hydroxycinnamic acid, predominantly trans	Antioxidant, reactive oxygen species (ROS) scavenging, anti-inflammatory, and antifibrotic activities
Flavonoids	Isorhamnetin, Tricin, Tectorigenin, Quercetin-3-O-rhamnoside, Tectochrysin, Nepetin-7-glucoside	Antioxidant activity, inhibition of TGF-β/Smad signaling, suppression of myofibroblast activation, and antifibrotic effects
Terpenoids and steroids	Diosgenin, quassin, aurapten, alpha-Santonin	Regulation of oxidative stress, inflammatory signaling, and cellular metabolism
Alkaloids and other nitrogen-containing compounds	Trigonelline, Higenamine, cinchonine, Corynoxeine, Dihydropalmatine, Conessine	Cytoprotection, antioxidant defense, and modulation of cellular signaling pathways
Fatty acids and lipid derivatives	Linoleic acid, Lauroylcarnitine, 9-HODE, N-lauroylethanolamine	Lipid metabolism, membrane homeostasis, oxidative stress response, and inflammatory regulation
Organic acids	Citric acid, Fumaric acid, Succinic acid, Azelaic acid, Sebacic acid, Glutaric acid, D-(+)-Malic acid	Energy metabolism, TCA cycle activity, and cellular redox regulation
Carbohydrates and sugar derivatives	Mannitol, Ribitol, Mannose, N-Acetylglucosamine, D-Sorbitol	Carbon metabolism, osmoprotection, and cellular energy homeostasis
Amino acids and derivatives	L-Arginine, L-Glutamine, L-Glutamic acid, L-Tryptophan, L-Histidine, Pyroglutamic acid	Protein biosynthesis, amino acid metabolism, collagen turnover, and antioxidant defense
Nucleobases, nucleosides, and nucleotides	Adenine, Adenosine, Guanosine, Inosine, Uridine, ADENOSINE 5′-MONOPHOSPHATE, Guanosine 5′-monophosphate, Uridine 5′-diphosphate	Nucleotide metabolism, RNA/DNA biosynthesis, cellular signaling, and proliferation
Fungal secondary metabolites	Mycophenolic acid, Viridicatin, Zearalenone	Characteristic fungal metabolites with diverse biological activities
Vitamins and cofactors	Pantothenic acid, Pantothenate	Coenzyme biosynthesis and cellular metabolism

Note. Representative entries are selected examples of putative LC-MS/MS annotations and do not constitute confirmed compound identifications. The complete positive- and negative-ion feature lists are provided in Tables S1 and S2. The literature-context column summarizes previously reported activities of similarly named compounds or chemical classes and does not demonstrate the identity, concentration, bioavailability, or causal contribution of any feature in the tested extract.

### 2.2. Effects of Truffle Extract on Cellular Metabolic Activity and Reducing Capacity

**Figure 1:**
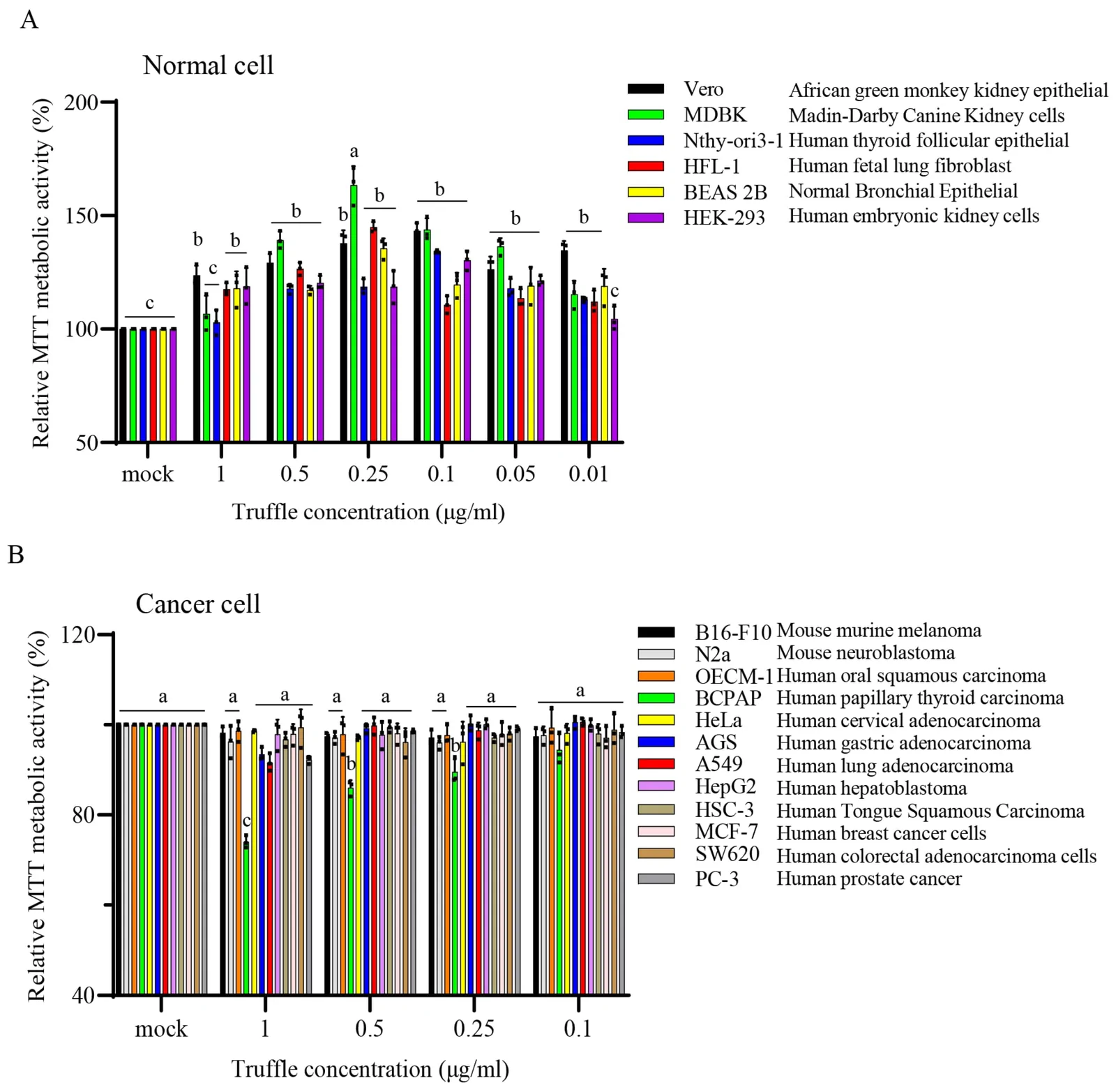
Effects of TE on cellular metabolic activity and reducing capacity in non-malignant and cancer cell lines after 24 h. (**A**) MTT-based metabolic activity and reducing capacity in six non-malignant cell lines treated with the indicated TE concentrations (0.01–1 μg/mL). (**B**) MTT-based metabolic activity and reducing capacity in twelve cancer cell lines treated with the indicated TE concentrations (0.1–1 μg/mL). Untreated controls were set to 100%. Data are presented as the mean ± SD from three independent experiments. Statistical differences were analyzed by one-way ANOVA followed by DMRT; different letters indicate significant differences among groups (*p* < 0.05). MTT-signal decreases are interpreted as reduced metabolic activity/reducing capacity rather than direct evidence of reduced cell number or cytotoxicity. Numerical values are provided in Tables S4 and S5.

To evaluate cellular metabolic activity and reducing capacity after TE exposure, six non-malignant cell lines were treated with 0.01–1 μg/mL TE, whereas twelve cancer cell lines were treated with 0.1–1 μg/mL TE for 24 h (Figure 1; Supplementary Tables S4 and S5). Across the non-malignant panel, treated-group values ranged from 102.25 ± 4.50% to 163.39 ± 6.82% of the untreated control; HFL-1 cells showed 144.90 ± 3.13% at 0.25 μg/mL TE. Because MTT reduction primarily reflects cellular metabolic and reducing capacity and may also be influenced by cell number, values above 100% were interpreted as increased MTT-based metabolic activity/reducing capacity rather than direct evidence of proliferation. In the cancer cell panel, the clearest concentration-associated decrease occurred in BCPAP cells, with MTT values of 91.62 ± 2.80%, 89.55 ± 2.30%, 86.01 ± 1.30%, and 75.51 ± 0.788% at 0.1, 0.25, 0.5, and 1 μg/mL TE, respectively. This decrease is described as reduced cellular metabolic activity/reducing capacity and is not, by itself, interpreted as proof of reduced cell number, cytotoxicity, or anticancer activity. The effects of TGF-β1 treatment alone on MTT-based cellular metabolic activity and reducing capacity in HFL-1 cells were also evaluated after 24 h at the indicated concentrations (Supplementary Figure S2).

### 2.3. Truffle Extract Modulates Kinase Phosphorylation and Attenuates TGF-β1-Induced Fibrotic Phenotypes

**Figure 2:**
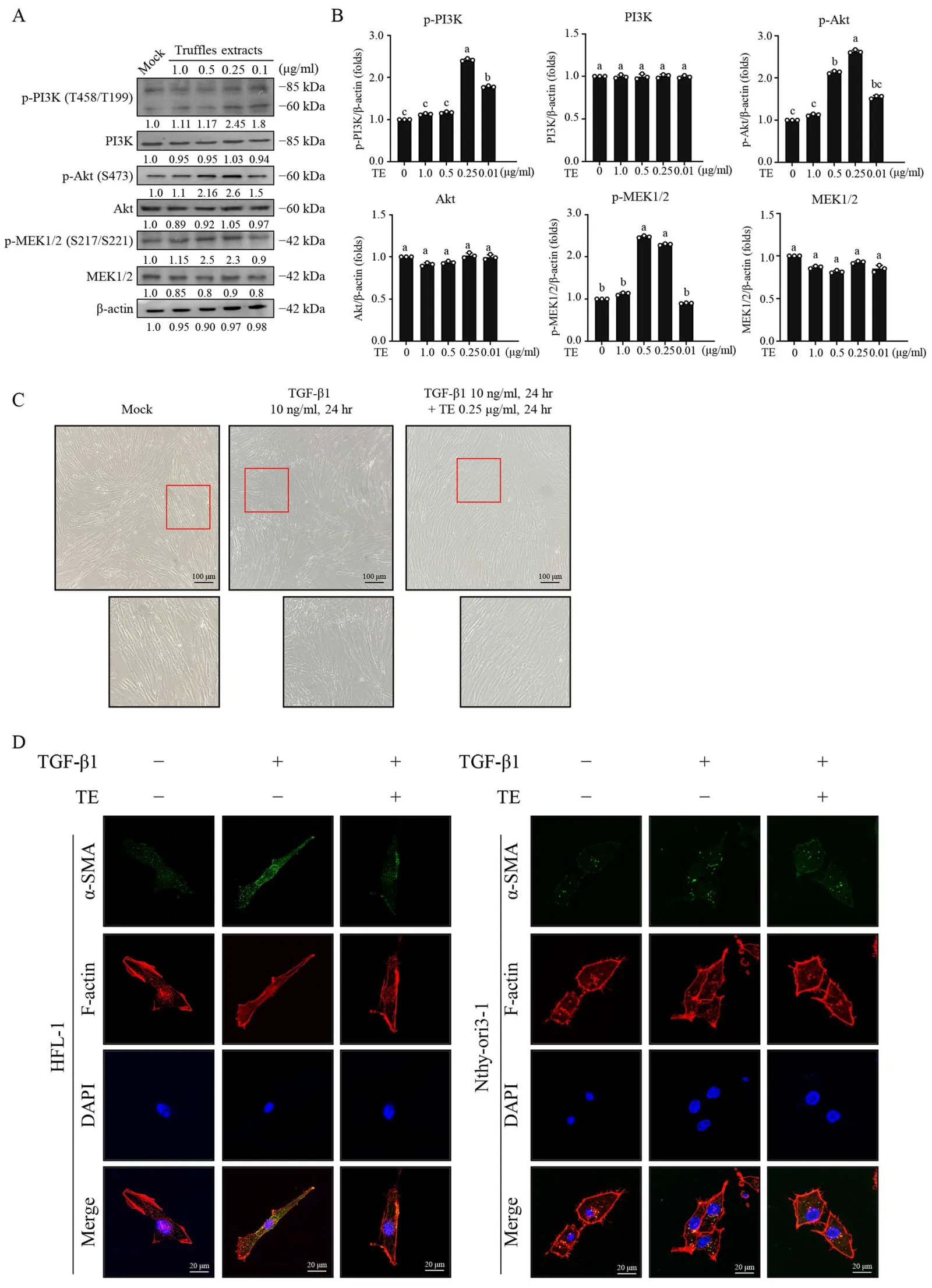
TE-associated kinase and fibrosis-related phenotypes in HFL-1 cells. (**A**) HFL-1 cells were treated with the indicated TE concentrations, and p-PI3K, PI3K, p-Akt, Akt, p-MEK1/2, and MEK1/2 were analyzed by Western blotting; β-actin was the loading control. (**B**) Phosphorylated and total proteins were quantified independently after normalization to β-actin. Data are mean ± SD from three independent experiments. Statistical differences were analyzed by one-way ANOVA followed by DMRT; different letters indicate *p* < 0.05. (**C**) Bright-field images of HFL-1 cells under the indicated TGF-β1 and TE conditions. Enlarged images correspond to the region indicated by the red box in the image. Representative images are from three independent experiments. Scale bar = 100 μm. (**D**) HFL-1 and Nthy-ori 3-1 cells were stained for α-SMA (green), F-actin with phalloidin (red), and nuclei with DAPI (blue). Nthy-ori 3-1 cells were included as a distinct non-malignant epithelial-cell imaging comparator and were not interpreted as an independent fibroblast model. Scale bar = 20 μm. Representative images and blots are shown; original/uncropped source files are provided in Figure S6 in the Supplementary Materials.

To characterize signaling responses potentially associated with TE, we examined kinase phosphorylation and fibrosis-related phenotypes. TE treatment increased the phosphorylation of PI3K, Akt, and MEK1/2, with the most pronounced responses observed at 0.25–0.5 μg/mL, whereas the corresponding total PI3K, Akt, and MEK1/2 levels remained largely unchanged (Figure 2A,B). In bright-field images, TGF-β1-treated HFL-1 cells displayed a more elongated and spread morphology, whereas TE co-treatment partially preserved a less-spread fibroblast morphology (Figure 2C). Immunofluorescence imaging showed increased α-SMA staining after TGF-β1 treatment and lower α-SMA staining after TE exposure; F-actin staining was included to visualize cellular architecture and was not quantitatively interpreted. Nthy-ori 3-1 cells served only as a distinct non-malignant epithelial-cell imaging comparator and were not interpreted as an independent fibroblast model. Therefore, the fibrosis-related conclusions are restricted to the HFL-1 in vitro model.

### 2.4. Truffle Extract Is Associated with Reduced TGF-β1-Induced Smad2 Phosphorylation and Fibrotic Marker Expression

**Figure 3:**
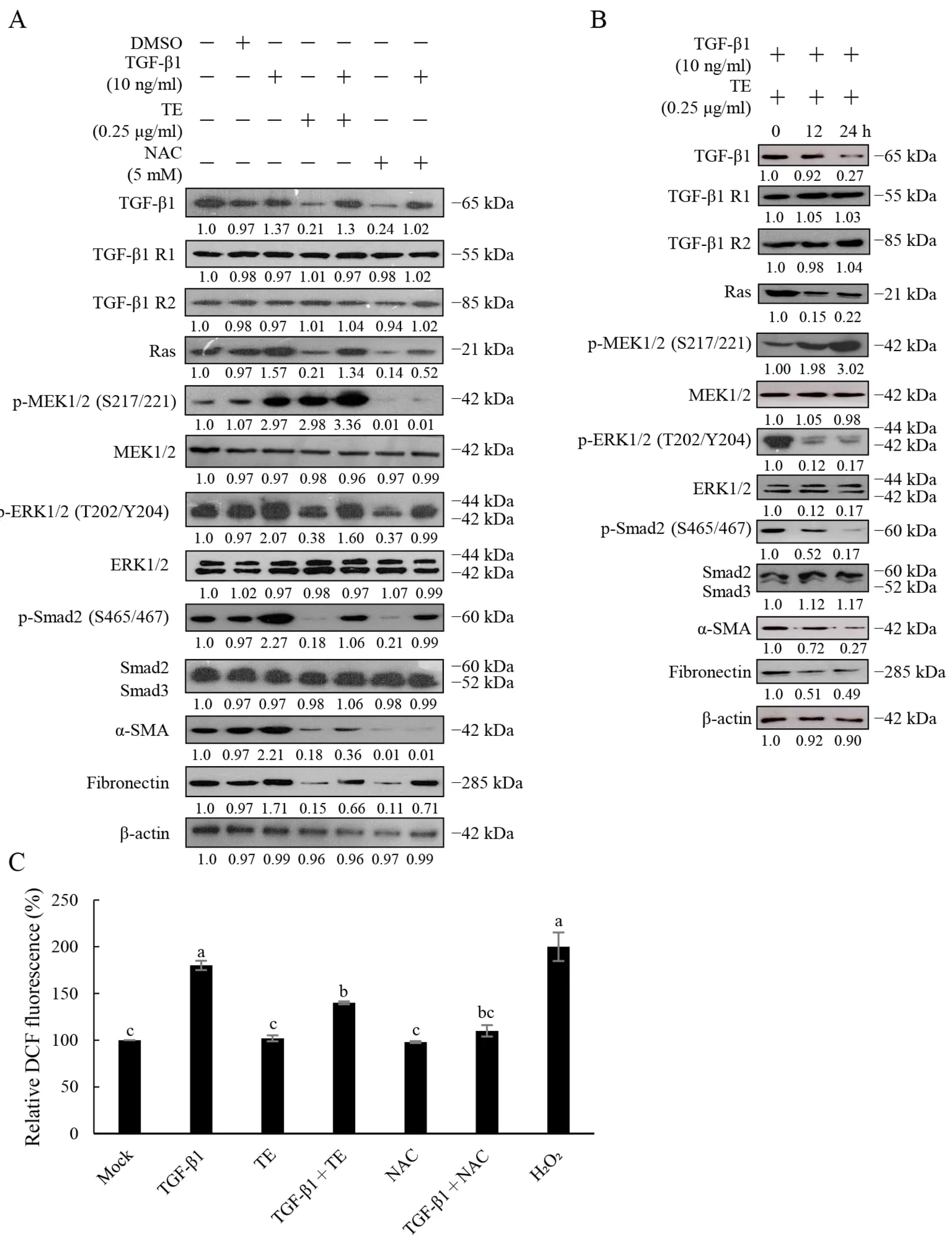
Effects of TE and NAC on TGF-β1-induced signaling and intracellular oxidative activity in HFL-1 cells. (**A**) For the post-treatment experiment, HFL-1 cells were first treated with TGF-β1 (10 ng/mL) for 24 h. After the medium was replaced, the cells were treated with TE (0.25 μg/mL) or NAC (5 mM) for an additional 24 h. Corresponding TE-only and NAC-only groups were treated with TE or NAC for 24 h without prior TGF-β1 exposure. TGF-β1, TGFBR1, TGFBR2, Ras, p-MEK1/2, MEK1/2, p-ERK1/2, ERK1/2, p-Smad2, total Smad2/3, α-SMA, and fibronectin were analyzed by Western blotting; β-actin was used as the loading control. (**B**) HFL-1 cells were simultaneously treated with TGF-β1 (10 ng/mL) and TE (0.25 μg/mL), and samples were collected at 0, 12, and 24 h for Western blotting of the indicated proteins. (**C**) Intracellular oxidation-dependent fluorescence was measured using 20 μM DCFH-DA after treatment with TGF-β1 (10 ng/mL), TE (0.25 μg/mL), NAC (5 mM), or the indicated combinations for 2 h. H_2_O_2_ (100 μM, 2 h) was included as a positive control. Fluorescence was measured at excitation and emission wavelengths of 495 and 529 nm, respectively. Data are presented as the mean ± SD from three independent experiments. One-way ANOVA followed by DMRT analyzed statistical differences; different letters indicate significant differences among groups (*p* < 0.05). Representative blots are shown, and Western blot quantification is provided in Figure S3.

To determine whether TE-associated phenotypic changes were accompanied by changes in canonical TGF-β1/Smad signaling, we examined Smad phosphorylation and fibrosis-associated proteins. TGF-β1 increased p-Smad2, α-SMA, and fibronectin. In the post-treatment experiment shown in Figure 3A, subsequent TE treatment for 24 h reduced these readouts relative to cells exposed to TGF-β1 alone. TGF-β1 increased p-MEK1/2, and subsequent TE treatment further increased p-MEK1/2 while reducing Ras and p-ERK1/2 relative to the TGF-β1-treated group. Subsequent NAC treatment produced a similar reduction in the matched TGF-β1-pretreated group. During simultaneous TGF-β1 and TE treatment, samples were collected at 0, 12, and 24 h. p-Smad2 and fibronectin decreased over time, while total Smad2/3 remained comparatively stable (Figure 3B). Across the TE-only, post-treatment, and simultaneous-treatment conditions, p-MEK1/2 increased alongside decreases in p-Smad2, α-SMA, and fibronectin, while TGFBR1 and TGFBR2 abundance remained largely unchanged.

Intracellular oxidative activity was further evaluated using the DCFH-DA assay (Figure 3C). TGF-β1 increased intracellular DCF fluorescence, whereas TGF-β1 plus TE and TGF-β1 plus NAC reduced the signal. Because DCFH-DA reports general oxidation-dependent fluorescence, these findings show that TE and NAC reduced the TGF-β1-induced increase in intracellular oxidative activity. Detailed densitometric quantification and statistical analyses of the Western blot results are shown in Figure S3.

### 2.5. Truffle Extract Reduces Smad2/3-Smad4 Association and Alters Smad Subcellular Distribution

**Figure 4:**
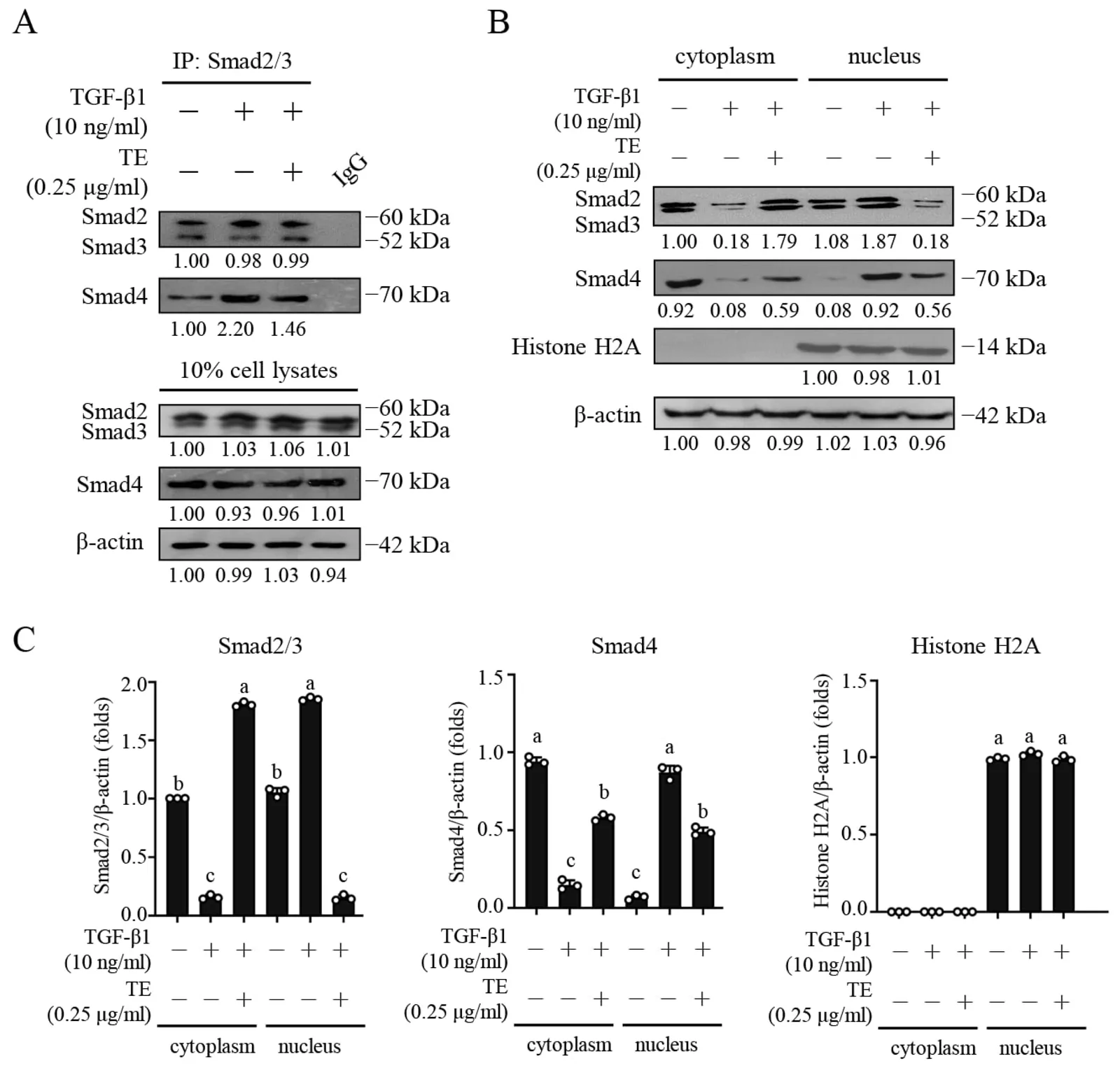
Effects of truffle extract on Smad2/3-Smad4 association and Smad subcellular distribution. (**A**) Co-immunoprecipitation analysis was performed in HFL-1 cells treated with TGF-β1 (10 ng/mL), with or without truffle extract (0.25 μg/mL), for 24 h. Cell lysates were immunoprecipitated with anti-Smad2/3 antibody, and the immunoprecipitates were analyzed by Western blotting using anti-Smad4 antibody. Ten percent of the cell lysates were analyzed as the input control, and rabbit IgG was included as a negative immunoprecipitation control. (**B**) Cytoplasmic and nuclear fractions were analyzed for Smad2/3 and Smad4 following TGF-β1 treatment with or without truffle extract. β-actin and histone H2A were used as cytoplasmic and nuclear fraction controls, respectively. (**C**) Densitometric quantification of Smad2/3, Smad4, and histone H2A in the cytoplasmic and nuclear fractions. TGF-β1 significantly increased nuclear Smad2/3 and Smad4 abundance while decreasing their cytoplasmic abundance. TE co-treatment significantly reduced nuclear Smad2/3 and Smad4 and increased their cytoplasmic abundance relative to TGF-β1 treatment alone. Histone H2A abundance remained comparable among the nuclear fractions. Data are presented as the mean ± SD from three independent experiments (*n* = 3). Statistical differences were analyzed by one-way ANOVA followed by Duncan’s multiple-range test; different letters indicate significant differences among groups (*p* < 0.05). For densitometry, local background was subtracted and each target, including proteins measured in nuclear fractions, was normalized to β-actin in the corresponding lane; histone H2A was used to document nuclear enrichment and was not used as the normalization denominator. The designated reference condition was set to 1.0 within each independent experiment before calculation of the mean ± SD.

To determine whether TE-associated changes in Smad2 phosphorylation were accompanied by changes in Smad complex formation and subcellular distribution, we examined Smad2/3-Smad4 association and cytoplasmic and nuclear protein abundance. As shown in Figure 4A, co-immunoprecipitation analysis indicated that TGF-β1 increased the association of Smad4 with immunoprecipitated Smad2/3 relative to the unstimulated control, whereas co-treatment with TE reduced this association relative to TGF-β1 alone. In the fractionation analysis (Figure 4B,C), TGF-β1 significantly increased the nuclear abundance of Smad2/3 and Smad4 while decreasing their cytoplasmic abundance. TE co-treatment reversed these changes, significantly reducing nuclear Smad2/3 and Smad4 and increasing their cytoplasmic abundance relative to TGF-β1 treatment alone. β-actin and histone H2A were used as cytoplasmic and nuclear fraction controls, respectively, and histone H2A abundance remained comparable among the nuclear fractions. Together, these findings show that TE reduced TGF-β1-induced Smad2/3-Smad4 association and reversed the nuclear accumulation of Smad2/3 and Smad4 under the tested conditions.

### 2.6. shRNA Analyses Further Characterize the TGF-β1/MAPK Signaling Context of the TE Response

**Figure 5:**
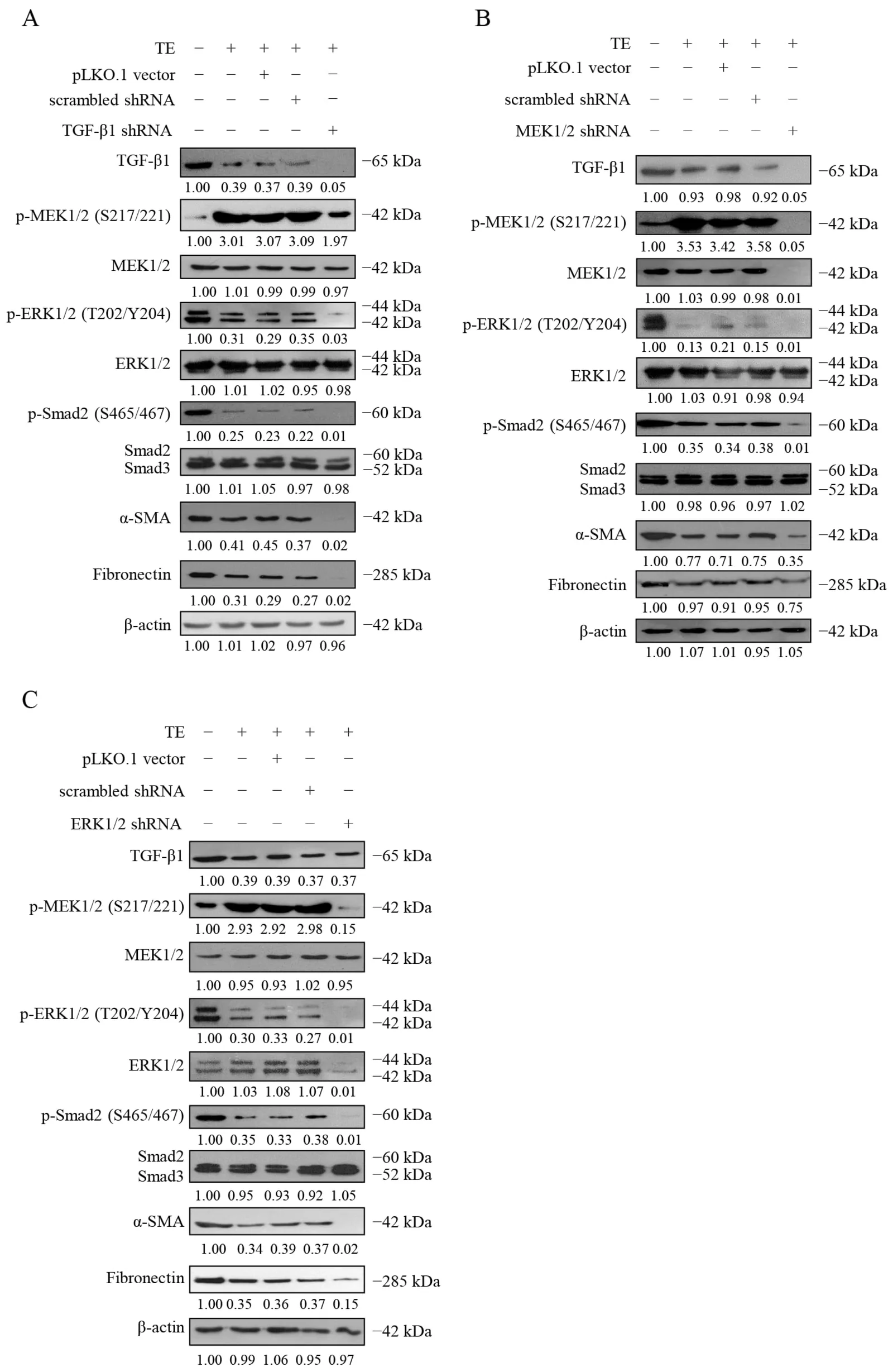
Truffle extract-mediated regulation of the TGF-β/MAPK signaling network was evaluated using specific shRNAs and Western blot analysis. (**A**–**C**) Effects of shRNA-mediated knockdown of TGF-β1 (**A**), MEK1/2 (**B**), and ERK1/2 (**C**) on TE-associated signaling and fibrosis-associated protein expression. Exogenous TGF-β1 was not added in the Figure 5 experiments. HFL-1 cells were transfected with the empty-vector control (pLKO.1), scrambled shRNA control (shNC), or target-specific shRNAs against TGF-β1, MEK1/2, or ERK1/2. Six hours after transfection, the cells were treated with TE (0.25 μg/mL) for an additional 24 h. The corresponding shRNA-only conditions without TE or exogenous TGF-β1, including target-protein reduction and fibrosis-associated protein expression, are presented in Supplementary Figure S4. Western blot analysis was performed to examine the indicated signaling and fibrosis-associated proteins. Representative blots from three independent experiments are shown. Densitometric data (mean ± SD, *n* = 3) and statistical comparisons (one-way ANOVA followed by DMRT; different letters indicate *p* < 0.05) are provided in Supplementary Figure S5.

Before evaluating the TE-treated shRNA experiments presented in Figure 5, the effects of the shRNA constructs alone were examined in the absence of TE and exogenous TGF-β1 (Supplementary Figure S4). HFL-1 cells were transfected with the empty-vector control (pLKO.1), scrambled shRNA control (shNC), or target-specific shRNAs against TGF-β1, MEK1/2, or ERK1/2. Western blotting confirmed reduced abundance of the corresponding target proteins and showed that the individual knockdowns also altered fibrosis-associated protein expression under TE-free conditions (Supplementary Figure S4A). Densitometric analyses from three independent experiments are shown in Supplementary Figure S4B. Thus, Supplementary Figure S4 provides the shRNA-only conditions for assessment of both target-protein reduction and fibrosis-associated protein expression.

The same shRNA constructs were subsequently used to examine the TE-associated response (Figure 5). HFL-1 cells were transfected with the indicated constructs, and 6 h after transfection, TE (0.25 μg/mL) was added for an additional 24 h. Exogenous TGF-β1 was not added in these experiments. In the TE-treated control conditions, pLKO.1 and shNC produced patterns similar to TE alone: p-MEK1/2 remained elevated, whereas endogenous TGF-β1, p-ERK1/2, p-Smad2, α-SMA, and fibronectin were lower than in untreated cells. TGF-β1 knockdown further reduced endogenous TGF-β1 and was accompanied by additional decreases in p-ERK1/2, p-Smad2, α-SMA, and fibronectin; p-MEK1/2 remained above the untreated level but was lower than in the TE-treated vector and shNC controls. MEK1/2 knockdown reduced total and phosphorylated MEK1/2 and was accompanied by a further reduction in α-SMA, whereas the fibronectin change was comparatively modest. ERK1/2 knockdown reduced total and phosphorylated ERK1/2 and was accompanied by marked decreases in both α-SMA and fibronectin. In the absence of TE, ERK1/2 knockdown reduced p-ERK1/2 without a corresponding decrease in p-MEK1/2 (Supplementary Figure S4). This response differed from that observed with TE alone, which was characterized by increased p-MEK1/2 together with decreased Ras and p-ERK1/2 (Figure 3). In contrast, ERK1/2 knockdown in TE-treated cells was accompanied by reduced p-MEK1/2 (Figure 5), indicating that the MEK1/2 phosphorylation response differed according to ERK1/2 knockdown and TE treatment status. Quantitative analyses and statistical comparisons are provided in Supplementary Figure S5. These results show that reduction in TGF-β1, MEK1/2, or ERK1/2 altered the TE-associated α-SMA and fibronectin patterns. The shRNA-only conditions in Supplementary Figure S4 allow the effects of the individual knockdowns on fibrosis-associated proteins to be considered separately from the TE-treated conditions shown in Figure 5. Together, these findings show that TGF-β1, MEK1/2, and ERK1/2 are associated with the signaling context in which the TE response occurs. However, because individual knockdowns also altered fibrosis-associated proteins in the absence of TE, the present data do not establish strict pathway dependence or a linear signaling hierarchy among these components.

## 3. Discussion

LC-MS/MS profiling provided exploratory compositional context for the tested aqueous preparation. The positive- and negative-ion datasets provide complementary views of ionizable features; however, the same annotation assigned in both modes represents mode-specific evidence and should not be counted as independent confirmation of a compound identity. The revised screening removed features that did not satisfy the final acceptance criteria, including assignments with unacceptable mass error or insufficient traceable support; these excluded features are not included in the final Supplementary Tables S1 and S2. Features showing acceptable support were retained only as putative annotations. This review emphasized that repeated annotations should be evaluated by integrating retention time, precursor *m*/*z*, ionization mode, adduct behavior, exact-mass agreement, and available spectral evidence, with peak area used only as a secondary selection criterion.

Accurate precursor-mass information can narrow candidate structures, but metabolomics reporting frameworks recommend comparison with authentic standards, including retention-time and MS/MS agreement, for the highest-confidence compound identification [22,23]. Accordingly, the retained names provide an exploratory list of putative LC-MS/MS annotations rather than a confirmed inventory of truffle constituents.

From a biological perspective, the cellular effects observed with the whole aqueous extract cannot be assigned to any single annotated feature. Several extract constituents may contribute independently or through additive, synergistic, or antagonistic interactions, and extraction and ionization biases may exclude bioactive constituents that are poorly recovered or detected under the present analytical conditions. Therefore, the chemical profile is most appropriately used to generate candidates for subsequent testing. Bioactivity-guided fractionation, followed by targeted quantitative LC-MS/MS, authentic-standard spiking, and biological retesting of active fractions or purified candidates, would be required to connect individual constituents with the fibrosis-associated cellular responses. Such iterative coupling of chemical characterization and biological validation is a central strategy in natural-product discovery [12]. Likewise, the exploratory protein database best matches provide hypothesis-generating information and should not be interpreted as confirmed protein identities or evidence of species-level biological origin.

Previous studies have reported antioxidant, cytoprotective, and cytotoxic activities for different truffle species and preparations [24,25,26,27]. These findings cannot be directly extrapolated to the present aqueous TE because the material, extraction procedures, chemical profiles, and experimental models differ. Because the MTT assay measures cellular reduction of tetrazolium to formazan and can be influenced by mitochondrial activity, cell number, and metabolic state, increased signals are described as increased cellular metabolic activity/reducing capacity rather than proliferation. Conversely, decreased MTT signals are described as reduced cellular metabolic activity/reducing capacity and are not interpreted alone as cytotoxicity, reduced cell number, or anticancer activity [28,29]. Direct cell counting, membrane-integrity assays, or DNA-synthesis measurements will be required to distinguish these possibilities.

Fibrosis is a complex pathological process characterized by persistent fibroblast activation and ECM deposition, ultimately leading to tissue remodeling and organ dysfunction. TGF-β1 sits at the core of fibrogenesis, signaling through both canonical Smad-dependent and non-canonical MAPK pathways [30,31,32,33]. In the HFL-1 model, TE attenuated TGF-β1-associated fibrotic responses, as evidenced by reduced α-SMA and fibronectin, along with preserved cellular morphology and MTT-based metabolic activity. Previous studies have shown that several natural compounds can modulate fibrosis-associated TGF-β1 signaling [34,35]. Similarly, mycelial polysaccharides from *Termitomyces albuminosus* attenuated CCl_4_-induced chronic liver injury in mice and were associated with suppression of TGF-β1/Smad3 and NF-κB signaling, providing comparative evidence that mushroom-derived polysaccharides may influence fibrosis-associated signaling pathways [36]. The present findings extend this area by showing that the tested aqueous truffle preparation is associated with coordinated changes in Smad-dependent fibrotic readouts and MAPK-network signaling.

TGF-β1 increased Smad2 and MEK1/2 phosphorylation, promoted Smad2/3-Smad4 association and the nuclear accumulation of Smad2/3 and Smad4, and elevated α-SMA and fibronectin in HFL-1 cells. TE reduced Smad2 phosphorylation, Smad2/3-Smad4 association, nuclear Smad2/3 and Smad4 abundance, α-SMA, and fibronectin. TE increased p-MEK1/2 while decreasing p-ERK1/2 and Ras expression. Thus, increased MEK1/2 phosphorylation was not propagated as downstream ERK1/2 activation. An additional indication of context-dependent MAPK regulation was observed in the ERK1/2 knockdown experiments. ERK1/2 knockdown alone reduced ERK1/2 and p-ERK1/2 without a corresponding decrease in p-MEK1/2, whereas ERK1/2 knockdown in TE-treated cells was accompanied by reduced p-MEK1/2. This differed from the response to TE in control-transfected cells, in which increased p-MEK1/2 occurred together with decreased Ras and p-ERK1/2. Because the Raf/MEK/ERK cascade is regulated by multiple feedback mechanisms, including ERK-dependent regulation of upstream pathway components and cellular MEK1/2 levels, these distinct responses may reflect feedback or compensatory regulation within the MAPK network rather than a simple linear relationship between MEK1/2 and ERK1/2 [33,34]. However, upstream Raf activity, Ras-GTP, phosphatase activity, and scaffold-dependent regulation were not examined in the present study. The mechanism responsible for the TE-dependent change in MEK1/2 phosphorylation following ERK1/2 knockdown therefore remains unresolved. The MAPK results are therefore interpreted as context-dependent and associative rather than as uniform activation or inhibition of the Ras/MEK/ERK pathway. Pathway-rescue, kinase-activity, and target-engagement experiments are required to establish causality.

Several established regulatory mechanisms could theoretically account for the observed increase in p-MEK1/2 together with reduced p-ERK1/2. First, attenuation of ERK-dependent negative feedback may permit upstream RAF-MEK phosphorylation to persist or accumulate. Second, induction or activation of ERK-directed dual-specificity phosphatases, such as DUSP6/MKP-3, could enhance ERK1/2 dephosphorylation despite continued MEK1/2 phosphorylation. Third, a MEK-directed allosteric effect could allow phosphorylated MEK1/2 to accumulate while reducing its capacity to phosphorylate ERK1/2, a response described for some allosteric MEK inhibitors. Fourth, altered assembly or localization of scaffold complexes involving proteins such as KSR or the MP1-p14 complex could reduce productive MEK-ERK coupling and spatial signal transmission [20,21,37,38]. These mechanisms are not mutually exclusive and are presented as testable explanations rather than demonstrated targets of TE. Time-resolved analysis of RAF activity, DUSP expression and activity, MEK kinase activity, MEK-ERK association, scaffold-protein abundance and localization, and fraction-guided target-engagement studies will be required to determine which mechanism, if any, contributes to the divergent phosphorylation pattern.

TGF-β is widely recognized as a master regulator of tissue fibrosis and inflammation, while the specificity and biological outcome of TGF-β-family signaling depend on cell type, signaling context, and integration of canonical and non-canonical pathways [30,39,40,41]. Oxidative stress and disruption of redox homeostasis are also closely associated with aging and chronic disease progression and may interact with profibrotic signaling networks [18,19]. These established frameworks provide broader biological context for the concurrent Smad, MAPK, and intracellular oxidative-activity changes observed in the present study.

The matched NAC comparison places intracellular oxidative activity within the response profile of TE. The parallel reduction in DCF fluorescence and fibrosis-associated markers is consistent with a redox-sensitive component of the cellular response, in agreement with the established interplay between oxidative homeostasis and profibrotic signaling [18,19]. NAC can modify cellular thiol status and multiple redox-responsive signaling processes [42]; therefore, the similar direction of the TE and NAC responses supports antioxidant-associated activity while leaving the precise molecular target open. DCFH-DA provides an integrated measure of intracellular oxidation-dependent fluorescence rather than species-level resolution [43,44]. Collectively, these observations identify reduced intracellular oxidative activity as one component associated with the TE response in TGF-β1-stimulated HFL-1 cells.

**Figure 6:**
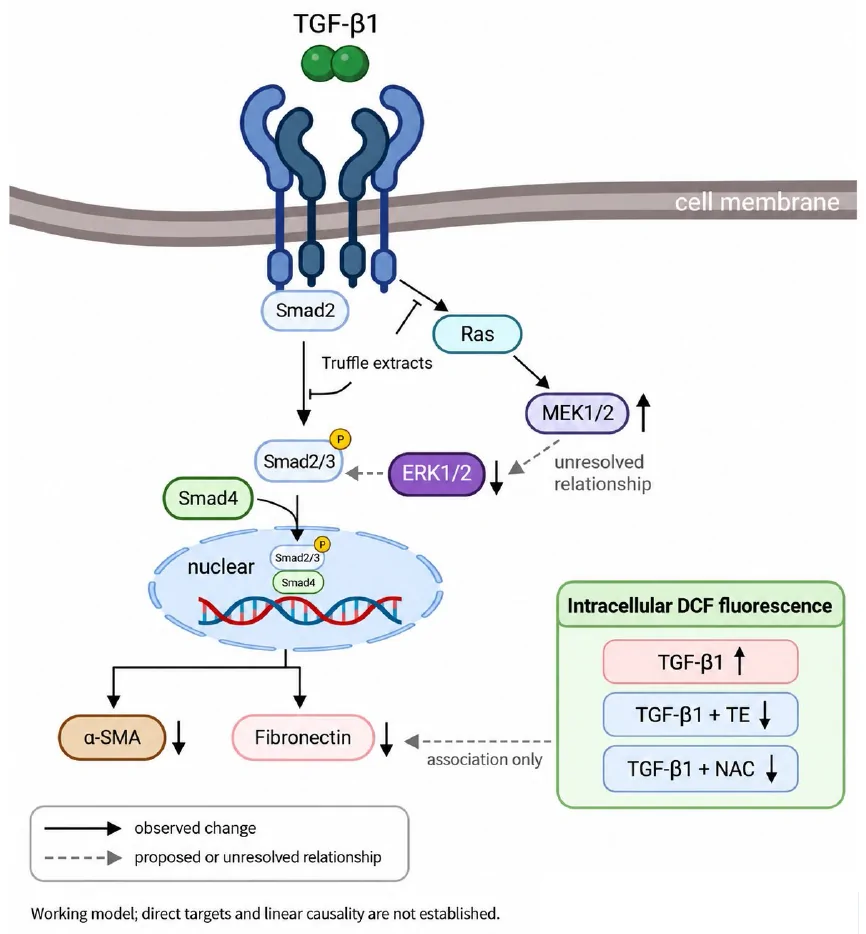
Working model of TE-associated signaling changes in the HFL-1 fibrosis model. TGF-β1 increased p-Smad2, Smad2/3-Smad4 association, nuclear Smad2/3 and Smad4 abundance, α-SMA, and fibronectin. TE reduced these Smad- and fibrosis-associated readouts and was associated with decreased Ras, increased p-MEK1/2, and decreased p-ERK1/2. The dashed p-MEK1/2-p-ERK1/2 connection denotes an unresolved signaling relationship. TGF-β1 increased intracellular DCF fluorescence, whereas TGF-β1 plus TE and TGF-β1 plus NAC reduced the signal. α-SMA and fibronectin are shown as parallel readouts, and the dashed DCF link denotes association only. Solid arrows indicate observed directions; dashed arrows indicate proposed or unresolved relationships. The model does not establish direct molecular targets, ROS scavenging as the sole mechanism, or a linear causal pathway. The grey dashed ERK1/2-to-Smad2/3 connection represents a proposed or unresolved relationship rather than an observed causal link; direct ERK1/2-to-Smad2/3 causality is not established by the present data. Created in BioRender. Li, J. (2026) https://BioRender.com/vs51wxf.

Future studies combining species-selective probes, antioxidant-enzyme measurements, and time-resolved oxidative interventions with Smad and MAPK readouts could determine whether the change in oxidative activity precedes and contributes to the attenuation of fibrosis-associated responses. Species-level authentication records, voucher-specimen information, and a batch/lot identifier were not made available by the supplier for the material used in this study. Accordingly, the findings apply specifically to the tested aqueous Tuber spp.-derived preparation and should not be generalized to a particular Tuber species. Independently authenticated material, voucher-specimen deposition, and batch-specific chemical standardization should be incorporated in future studies. In summary, the tested aqueous TE was associated with reduced canonical Smad signaling, lower levels of α-SMA and fibronectin, and altered MAPK network readouts in HFL-1 cells. The reproducible increase in p-MEK1/2 together with reduced fibrosis-associated markers indicates unresolved pathway crosstalk rather than simple MEK inhibition. These findings highlight the fibrosis-modulating activity of the whole aqueous preparation in vitro. Future validation in additional fibroblast and in vivo models, together with activity-guided chemical characterization, will help assess the broader biological and translational relevance of these observations (Figure 6).

## 4. Materials and Methods

### 4.1. Truffles

The truffle material used in this study was obtained from Tsairder Biotechnology Co. (Taichung, Taiwan). The supplier did not provide species-level authentication records, voucher-specimen information, or a batch/lot identifier for the material used in this study. Accordingly, the material is conservatively described as Tuber spp.-derived material, and the aqueous preparation is referred to as truffle extract (TE). Dried material (100 g) was ground and passed through an 80-mesh sieve. The resulting powder was extracted with 1000 mL of distilled water at 65 °C for 1 h under constant agitation, corresponding to a solid-to-liquid ratio of 1:10 (*w*/*v*). After extraction, the aqueous extract was collected, filtered, and concentrated under reduced pressure using a rotary evaporator maintained at 40 ± 5 °C. The concentrated extract was subsequently lyophilized for 72 h. Across more than 10 independent extractions, the crude extraction yield ranged from approximately 12% to 15% (*w*/*w*), calculated as the weight of the freeze-dried crude extract relative to the initial dry weight of the truffle material. The freeze-dried powder was stored at −20 °C until use. Related hot-water extraction approaches have previously been reported for mushroom-derived polysaccharide preparations [45]. For cell-culture experiments, the freeze-dried powder was dissolved in culture medium, sterilized through a 0.22-μm membrane filter, and applied at the indicated concentrations.

### 4.2. LC-MS/MS Sample Preparation, Data Processing, and Compound Annotation

For untargeted metabolite LC-MS/MS profiling, 10 mg of the freeze-dried aqueous truffle extract was reconstituted in 0.1 mL of distilled water to obtain a final concentration of 100 mg/mL (10%, *w*/*v*). The reconstituted extract was filtered through a 0.22-μm membrane before LC-MS/MS analysis. Small-molecule LC-MS/MS analysis was performed using an UltiMate 3000 RSLCnano LC system (Thermo Fisher Scientific, Waltham, MA, USA) coupled to a TripleTOF 6600 mass spectrometer (SCIEX, Framingham, MA, USA). Chromatographic separation was performed on a T3 C18 column (3 μm, 2.1 × 100 mm) maintained at 35 °C. Mobile phase A consisted of distilled water containing 0.1% formic acid, and mobile phase B consisted of acetonitrile containing 0.1% formic acid. The flow rate was 250 μL/min, and the injection volume was 5 μL. The gradient was programmed as follows: 5% B at 0–2 min, 50% B at 10 min, 80% B at 20 min, 90% B at 27–30 min, and 5% B at 30.1–35 min. Data were acquired separately in positive- and negative-ion modes. The ion-spray voltages were +5500 and −4500 V, the declustering potentials were +80 and −80 V, and the collision energies were +10 and −10 V for the positive- and negative-ion modes, respectively. The curtain gas, ion source gas 1, and ion source gas 2 settings were 17, 19, and 29, respectively, and the source temperature was 150 °C. TOF-MS and TOF-MS/MS spectra were acquired over an *m*/*z* range of 50–1000 using accumulation times of 250 and 100 ms, respectively. Information-dependent acquisition selected up to 20 precursor ions per cycle using an intensity threshold of 100, a mass tolerance of 50 ppm, a dynamic-exclusion duration of 6 s, and a collision-energy spread of 15 V.

For proteomic analysis, the freeze-dried aqueous truffle extract was reconstituted in 8 M urea prepared in 50 mM ammonium bicarbonate. Proteins were reduced with 10 mM DTT at 56 °C for 30 min and alkylated with 20 mM iodoacetamide in the dark for 30 min. The samples were diluted with 50 mM ammonium bicarbonate to reduce the urea concentration to below 1 M and were digested with sequencing-grade trypsin at an enzyme-to-protein ratio of 1:50 (*w*/*w*) at 37 °C for 16 h. The resulting peptides were desalted using C18 ZipTips before LC-MS/MS analysis. Peptides (1 μg per analysis) were separated using an UltiMate 3000 RSLCnano system (Dionex, Thermo Fisher Scientific, Waltham, MA, USA) coupled to a TripleTOF 6600 mass spectrometer equipped with a Nanospray III ion source (SCIEX, Framingham, MA, USA). Peptides were first loaded onto an Acclaim PepMap C18 trap column (100 μm × 2 cm; nanoViper C18, 5 μm, 100 Å; Thermo Fisher Scientific, Waltham, MA, USA) and subsequently separated on a bioZen Peptide XB-C18 Nano column (2.6 μm, 250 × 0.075 mm; Phenomenex, Torrance, CA, USA). Mobile phase A consisted of 0.1% formic acid in ddH_2_O, and mobile phase B consisted of 0.1% formic acid in acetonitrile. Chromatographic separation was performed over 150 min at a flow rate of 0.4 μL/min. Data-dependent acquisition used precursor-ion and product-ion mass ranges of *m*/*z* 350–1250 and *m*/*z* 65–1800, respectively. The acquired spectra were searched using Mascot version 3.1 (Matrix Science, London, UK) against the SwissProt sprot database. Search parameters included trypsin specificity, a maximum of one missed cleavage, monoisotopic masses, a peptide-mass tolerance of 0.05 Da, and a fragment-mass tolerance of 0.03 Da. The reported modifications were carbamidomethylation (C), deamidation (NQ), oxidation (HW), and oxidation (M). The search included all taxonomy entries and used a significance threshold of *p* < 0.05. After score-based filtering, 84 exploratory database best-match entries with at least one matched peptide with a Mascot Ions Score > 43 (*p* < 0.05 according to the Mascot search report) were retained. Protein annotations were taken from the Mascot report, and the functional categories in Supplementary Table S3 are used only for descriptive organization. These entries are conservatively reported as exploratory matches rather than confirmed protein identities, species-level assignments, functional validation, or evidence of the biological origins of the matched organisms. The corresponding peptide-level Mascot search output is provided in Datasheet 1 of the Supplementary Information. The archived search was performed on 17 February 2023 against SwissProt sprot (UniProt release 2022_05; 563,552 sequences and 203,007,781 residues). The search output comprised 133 protein-family groups. Datasheet 1 distinguishes 220 single-entry peptide rows from 41 shared peptide rows; shared peptides were not interpreted as uniquely supporting a single protein or species. Because validated false-discovery-rate information was unavailable in the archived output, no confirmed protein identity is claimed, and the Ions Score > 43 threshold is used only as the archived search-specific exploratory significance criterion rather than as a universal protein-validation cutoff.

Small-molecule LC-MS/MS raw data were processed using MS-DIAL software (version 5.5.250820) [46]. The processing workflow was configured to search the public MS-DIAL spectral libraries MS/MS_public_all-pos-VS19 and MS/MS_public_all-neg-VS19 for the positive- and negative-ion datasets, respectively. Processing parameters included a minimum peak height of 30 amplitude, a minimum peak width of 5 scans, an MS1 mass range of *m*/*z* 100–1000, an MS/MS mass range of *m*/*z* 50–1000, an MS1 tolerance of 0.05 Da, and an MS/MS tolerance of 0.05 Da. The MS-DIAL library-search processing settings included a dot-product threshold of ≥150, a weighted dot-product threshold of ≥150, a reverse dot-product threshold of ≥500, and a minimum matched-fragment setting of 2. The minimum matched-fragment setting of 2 was used as a processing parameter and was not applied as a post hoc requirement for final annotation retention because the number of library fragments available for comparison varied among reference spectra. For final feature curation, an absolute precursor-ion mass error of ≤30 ppm was used as the mass-error acceptance criterion [47,48,49]. Candidates with at least two matched fragments were retained; candidates with one matched fragment were retained only when the matched-peaks percentage was 100%. Supplementary Tables S1 and S2 report retention time, observed precursor *m*/*z*, adduct type, calculated *m*/*z*, mass error (ppm), candidate formula, and matched-fragment count for the listed features. Because authentic reference standards were not analyzed, retained names are reported conservatively as putative candidate annotations rather than confirmed compounds or level-1 identifications. For final annotation curation, observed precursor *m*/*z* values were compared with the expected [M + H]^+^ or [M – H]^−^ candidate ions together with calculated exact mass, mass error, retention time, ionization mode, same-name feature relationships, matched-fragment information, and other available archived evidence. Candidates with unacceptable mass error, no matched-fragment support, insufficient spectral support, or other insufficient traceable evidence were excluded from the final Supplementary Tables S1 and S2; peak area was used only as a secondary descriptive criterion. Database organism or source labels were not interpreted as evidence of biological origin.

### 4.3. Cell Culture

All cell lines used in this study were maintained at 37 °C in a humidified incubator with 5% CO_2_ and subcultured at 70–80% confluence. Non-malignant cells included Vero (African green monkey kidney epithelial cells) cultured in Minimum Essential Medium (MEM); MDBK (Madin–Darby bovine kidney cells), Nthy-ori 3-1 (human normal thyroid follicular epithelial cells), BEAS-2B (human bronchial epithelial cells), and HEK-293 (human embryonic kidney cells) cultured in Dulbecco’s modified Eagle’s medium (DMEM); and HFL-1 human lung fibroblasts (ATCC^®^ CCL-153™) maintained in Ham’s F-12K (Kaighn’s) medium supplemented with 10 mM HEPES (pH 7.0–7.2). Cancer cell lines included B16-F10 (mouse melanoma), N2a (mouse neuroblastoma), BCPAP (human papillary thyroid carcinoma), OECM-1 (human oral squamous cell carcinoma), HeLa (human cervical carcinoma), AGS (human gastric adenocarcinoma), A549 (human lung adenocarcinoma), HepG2 (Human hepatoblastoma), HSC-3 (human oral squamous cell carcinoma), MCF-7 (human breast adenocarcinoma), PC-3 (human prostate adenocarcinoma), and SW620 (human colorectal adenocarcinoma). BCPAP cells were cultured in RPMI-1640, AGS cells in Ham’s F-12K, and all other cancer cell lines in DMEM. All media were supplemented with 10% fetal bovine serum and 1% penicillin–streptomycin.

### 4.4. MTT Assay

Cellular metabolic activity and reducing capacity were assessed using the MTT assay with minor modifications from the method described by Mosmann [29]. Cells were seeded in 96-well plates and treated with the indicated concentrations of TE or TGF-β1 for 24 or 72 h. After treatment, 20 μL of MTT solution (5 mg/mL in PBS) was added to each well, and the plates were incubated at 37 °C for 4 h. The culture medium was carefully removed, and 200 μL of DMSO was added to dissolve the formazan crystals. Plates were shaken for 10 min at room temperature, and absorbance was measured at 570 nm with a reference wavelength of 630 nm. Results were expressed relative to the untreated control. A decreased MTT signal was interpreted as reduced cellular metabolic activity/reducing capacity and not, by itself, as evidence of reduced cell number or cytotoxicity.

### 4.5. Antibodies and Inhibitors

The catalog numbers and working dilutions of all primary and secondary antibodies used in this study are provided in Table 2. Secondary antibodies, including anti-mouse IgG (H + L) and anti-rabbit IgG (H + L), were obtained from KPL (Gaithersburg, MD, USA). N-acetyl-L-cysteine (NAC) was used as an antioxidant comparator and was purchased from Sigma-Aldrich (St. Louis, MO, USA). NAC was dissolved in sterile distilled water to prepare a stock solution before use. Recombinant TGF-β1 was purchased from Sigma-Aldrich and used according to the experimental design. TGF-β1 is a multifunctional cytokine involved in cell proliferation, differentiation, epithelial–mesenchymal transition, and fibrotic responses through Smad-dependent and Smad-independent signaling pathways [50,51].

### 4.6. Immunofluorescence Staining

HFL-1 and Nthy-ori 3-1 cells were seeded onto sterile glass coverslips in 24-well plates and cultured overnight. The cells were then treated with TGF-β1 in the presence or absence of truffle extract for the indicated time periods. After treatment, the cells were washed three times with phosphate-buffered saline (PBS), fixed with 4% paraformaldehyde for 15 min at room temperature, permeabilized with 0.1% Triton X-100 in PBS for 10 min, and blocked with 5% bovine serum albumin (BSA) for 1 h at room temperature. The cells were incubated overnight at 4 °C with a primary antibody against α-SMA. After washing with PBS, the cells were incubated with a FITC-labeled goat anti-mouse IgG (H+L) secondary antibody, for 1 h at room temperature in the dark. To visualize the actin cytoskeleton, the cells were stained with DyLight^®^ 554 phalloidin according to the manufacturer’s instructions. Cell nuclei were counterstained with 4′,6-diamidino-2-phenylindole (DAPI). Coverslips were mounted with antifade mounting medium, and fluorescence images were acquired using a fluorescence microscope under identical exposure settings for all experimental groups.

### 4.7. TGF-β1 Stimulation, TE Treatment, and NAC Control

For the post-treatment protocol used in Figure 3A, cells were stimulated with recombinant human TGF-β1 (10 ng/mL) for 24 h. The medium was then replaced, and TE (0.25 μg/mL) or NAC (5 mM) was added for an additional 24 h. Corresponding TE-only and NAC-only controls received TE or NAC for 24 h without prior TGF-β1 exposure. For the simultaneous-treatment experiment shown in Figure 3B, TGF-β1 (10 ng/mL) and TE (0.25 μg/mL) were added together, and samples were collected at 0, 12, and 24 h. NAC was used as a matched antioxidant comparator [42].

### 4.8. Intracellular DCF Fluorescence Assay

Intracellular oxidative activity was assessed using the DCFDA/H2DCFDA Cellular ROS Assay Kit (ab113851, Abcam, Cambridge, UK) according to the manufacturer’s instructions and established DCFDA-based methodology [44]. HFL-1 cells were seeded in black, clear-bottom 96-well plates at a density of 5 × 10^4^ cells per well. Cells were incubated with 20 μM DCFH-DA for 30 min at 37 °C in the dark and washed to remove extracellular probe. The cells were subsequently treated for 2 h with TGF-β1 (10 ng/mL), TE (0.25 μg/mL), NAC (5 mM), or the indicated combinations; these concentrations were consistent with those used in the corresponding experiments. H_2_O_2_ (100 μM, 2 h) was included as a positive control. Fluorescence intensity was measured at an excitation wavelength of 495 nm and an emission wavelength of 529 nm. After subtraction of the corresponding background fluorescence, the DCF signal was expressed relative to that of the untreated control. Experiments were performed independently three times.

### 4.9. shRNA Construction and Transfection

The shRNA sequences used in this study were as follows: TGFB1 (TRCN0000003318), 5′-CCGGCCTTTCCTGCTTCTCAT-3′; ERK1/2 (TRCN0000010040), 5′-CAAAGTTCGAGTAGCTATCAA-3′; and MEK1/2 (TRCN0000002329), 5′-GCTTCTATGGTGCGTTCTACA-3′. All shRNA plasmids were purchased from the RNA Technology Platform and Gene Manipulation Core Facility, Academia Sinica, Taipei, Taiwan. Cells were transfected with the indicated shRNA plasmids using jetPRIME Transfection Reagent (Polyplus-transfection SA, Illkirch, France) according to the manufacturer’s instructions. For the shRNA-only experiments shown in Supplementary Figure S4, HFL-1 cells were transfected with pLKO.1, shNC, or target-specific shRNAs against TGF-β1, MEK1/2, or ERK1/2 without TE or exogenous TGF-β1 treatment, and target-protein reduction and fibrosis-associated protein expression were examined by Western blotting. For the TE-treated shRNA experiments shown in Figure 5, HFL-1 cells were transfected with the same constructs. Six hours after transfection, the medium was replaced, and TE (0.25 μg/mL) was added for an additional 24 h. Exogenous TGF-β1 was not added in these experiments. Cells were then harvested for protein extraction and subsequent analysis.

### 4.10. Cytoplasmic and Nuclear Fractionation

Cytoplasmic and nuclear proteins were separated using the CNM Compartmental Protein Extraction Kit (BioChain Institute Inc., Newark, CA, USA) according to the manufacturer’s instructions. Briefly, cells were harvested and sequentially fractionated to obtain cytoplasmic and nuclear extracts. The purity of fractions was confirmed using compartment-specific marker proteins, and samples were subjected to Western blot analysis.

### 4.11. Electrophoresis and Western Blotting

Cells were seeded in 6-well plates one day before treatment or transfection. After treatment, cells were washed twice with ice-cold PBS and lysed using lysis buffer (Cell Signaling Technology, Danvers, MA, USA). Protein concentrations were determined using the Bio-Rad protein assay (Bio-Rad, Hercules, CA, USA) following the manufacturer’s instructions. Equal amounts of protein were mixed with 5× Laemmli loading buffer, boiled for 10–20 min, and separated by SDS-PAGE on 10% or 12% polyacrylamide gels. Proteins were transferred to PVDF membranes (GE Healthcare Life Sciences, Chicago, IL, USA), blocked, and incubated with specific primary antibodies, followed by horseradish peroxidase (HRP)-conjugated secondary antibodies. Signals were detected using enhanced chemiluminescence (ECL Plus, Amersham Biosciences, Piscataway, NJ, USA) and visualized on X-ray film (Kodak, Rochester, NY, USA). Band intensities were quantified using ImageJ software (version 1.54; National Institutes of Health, Bethesda, MD, USA). For densitometric analysis, local background was subtracted from each band, and the signal intensity of each target protein was normalized to β-actin in the corresponding lane. For nuclear-fraction analyses, histone H2A was used to verify nuclear enrichment but was not used as the normalization denominator; nuclear target proteins were likewise normalized to β-actin. Total and phosphorylated forms of signaling proteins were quantified independently after β-actin normalization. Accordingly, phosphorylated-protein signals are presented as relative phosphorylated-protein abundance rather than as phosphoprotein/total-protein ratios. Within each independent experiment, the designated reference condition was set to 1.0, and normalized values from three independent experiments were used to calculate the mean ± SD. Replicate-level densitometric calculations and representative replicate blots are provided in the reviewer-only supporting file.

### 4.12. Co-Immunoprecipitation Assays

To investigate the association between Smad2/3 and Smad4, co-immunoprecipitation assays were performed using the Catch and Release Reversible Immunoprecipitation System (Millipore, Merck Ltd., Taipei, Taiwan) as previously described [52,53]. HFL-1 cells were seeded in 6-cm dishes and cultured in Ham’s F-12K medium supplemented with 5% FBS at 37 °C in a humidified incubator with 5% CO_2_ until they reached approximately 75% confluence. Cells were treated with TGF-β1 (10 ng/mL), with or without TE (0.25 μg/mL), for 24 h and were then harvested for protein extraction. After two washes with 1× PBS, cells were scraped and lysed in 200 μL of CHAPS lysis buffer (40 mM HEPES [pH 7.5], 120 mM NaCl, 1 mM EDTA, 10 mM sodium pyrophosphate, 10 mM β-glycerophosphate, 50 mM NaF, and 0.3% CHAPS). For immunoprecipitation, 1000 μg of total protein was incubated overnight at 4 °C with 4 μg of anti-Smad2/3 antibody. The immunoprecipitated complexes were separated by SDS-PAGE and analyzed by Western blotting using an anti-Smad4 antibody. Rabbit IgG was used as a negative control to assess the specificity of the immunoprecipitation.

### 4.13. Statistical Analysis

All quantitative data are presented as the mean ± standard deviation (SD) from three independent experiments. Differences among groups were analyzed by one-way analysis of variance (ANOVA) followed by Duncan’s multiple-range test (DMRT) using GraphPad Prism 9 software (GraphPad Software, San Diego, CA, USA). Groups with different letters are significantly different, whereas groups sharing the same letter are not significantly different. A *p*-value < 0.05 was considered statistically significant.

## 5. Conclusions

In conclusion, the tested aqueous truffle extract attenuated multiple TGF-β1-induced fibrosis-associated responses in the HFL-1 in vitro model. TE reduced Smad2 phosphorylation, Smad2/3-Smad4 complex formation, nuclear accumulation of Smad2/3 and Smad4, and the expression of α-SMA and fibronectin, supporting an association between TE treatment and suppression of canonical TGF-β/Smad signaling. In parallel, TE decreased Ras expression and p-ERK1/2 while reproducibly increasing p-MEK1/2. This discordant phosphorylation pattern indicates context-dependent regulation and unresolved crosstalk within the MAPK network rather than simple activation or inhibition of a linear Ras/MEK/ERK pathway. The shRNA experiments further showed that reduction in TGF-β1, MEK1/2, or ERK1/2 altered fibrosis-associated protein expression under TE-free conditions and modified the TE-associated α-SMA and fibronectin responses, indicating that the TE response occurred within a signaling context influenced by these components while not establishing strict pathway dependence, a linear signaling hierarchy, or a direct molecular target of TE. TE and NAC also reduced the TGF-β1-induced increase in intracellular DCF fluorescence, linking the fibrosis-associated response to reduced intracellular oxidative activity without establishing ROS scavenging as the sole mechanism. Together, the Smad, MAPK, shRNA, and DCFH-DA findings support a multifaceted cellular response to the whole aqueous extract. LC-MS/MS profiling provides exploratory compositional context for this response, but the putative LC-MS/MS annotation names and protein database best matches remain exploratory and unconfirmed and cannot identify the active constituent or establish species-level protein origins. Likewise, MTT measurements are interpreted as changes in cellular metabolic activity/reducing capacity rather than direct evidence of proliferation or cytotoxicity. Because species-level authentication of the starting material was unavailable, these conclusions apply specifically to the tested aqueous Tuber spp.-derived preparation. Future studies using authenticated material, bioactivity-guided fractionation, targeted chemical validation, pathway-rescue or target-engagement approaches, and additional fibroblast and in vivo models will be required to define the active constituents and determine the broader biological and translational relevance of these findings.

## Figures and Tables

**Table 2:** The catalog numbers and dilution factors of the respective antibodies used in this study.

Antibodies	Catalog Numbers	Clone Name	Dilution Factor	Manufacturer
Rabbit anti-PI3K	4263	D9A5	3000	Cell Signaling
Rabbit anti-p-PI3K (T458/T199) *	4228	-	2000	Cell Signaling
Rabbit anti-Akt	4691	C67E7	3000	Cell Signaling
Rabbit anti-p-Akt (S473)	3787	736E11	2000	Cell Signaling
Rabbit anti-p-ERK1/2 (T202/Y204)	4370	D13.14.4E	3000	Cell Signaling
Rabbit anti-ERK1/2	4695	137F5	3000	Cell Signaling
Rabbit-anti-p-MEK1/2 (S217/S221)	9154	41G9	1500	Cell Signaling
Rabbit-anti-MEK1/2	8782	D1A5	3000	Cell Signaling
Rabbit-anti-p-Smad2 (S465/S467)	18338	E8F3R	2000	Cell Signaling
Rabbit-anti-Smad2/3	8685	D7G7	3000	Cell Signaling
Mouse-anti-α-SMA	19245	D4K9N	3000	Cell Signaling
Rabbit-anti-Fibronectin	26836	E5H6X	3000	Cell Signaling
Rabbit-anti-Ras	8955	D2C1	3000	Cell Signaling
Rabbit-anti-Smad4	46535		3000	Cell Signaling
Rabbit-anti-TGF-β1	3711		3000	Cell Signaling
Rabbit-anti-TGF-β1 receptor I	49728	F6L3I	3000	Cell Signaling
Rabbit-anti-TGF-β1 receptor II	41896	E5M6F	3000	Cell Signaling
Mouse anti-Histone H2A *	2578		3000	Cell Signaling
Mouse anti-β-actin	MAB1501	C4	10,000	Millipore
DyLight^®^ 554 Phalloidin	13054		2000	Cell Signaling
Goat anti-mouse IgG (H + L) HRP	5220-0341	-	5000	SeraCare
Goat anti-rabbit IgG (H + L) HRP	5220-0336	-	5000	SeraCare
Goat Anti-Mouse IgG (H+L) FITC-Labeled *	5230-0307		1000	SeraCare

* Polyclonal antibodies.

## Data Availability

The data that support the findings of our study are available on request from the corresponding author.

## Supplementary Materials

The following supporting information can be downloaded at https://www.mdpi.com/article/10.3390/ijms272110030/s1.

## Abbreviations

The following abbreviations are used in this manuscript:

Akt	Protein kinase B
co-IP	Co-immunoprecipitation
DMRT	Duncan’s multiple-range test
ECM	Extracellular matrix
FBS	Fetal bovine serum
HEPES	4-(2-hydroxyethyl)piperazine-1-ethanesulfonic acid
HRP	Horseradish peroxidase
JNK	c-Jun N-terminal kinase
MAPK	Mitogen-activated protein kinase
ERK	Extracellular signal-regulated kinases
MTT	3-(4,5-Dimethylthiazol-2-yl)-2,5-diphenyltetrazolium bromide
NAC	N-acetylcysteine
PI3K	Phosphoinositide-3-kinase
TGF-β	Transforming growth factor-beta
TGFBR1	Transforming growth factor-beta receptor I
TGFBR2	Transforming growth factor-beta receptor II
α-SMA	α-smooth muscle actin
