# Supplementary material for: Truffle Extract Attenuates TGF-β1-Induced Fibrosis-Associated Responses in HFL-1 Cells and Is Associated with Changes in Smad and MAPK Signaling
Source: Int J Mol Sci. 2026 Nov 9;27(21):10030. doi: 10.3390/ijms272110030 (PMC13763988; doi:10.3390/ijms272110030)
Supplement: Supplementary file 1 [file ijms-27-10030-s001.zip › ijms-4494130-supplementary.pdf]

Supplementary materials

Table S1. Putatively annotated LC-ESI(+)-MS/MS features retained after final screening.

Chemical class	Putative annotation	Peak area	RT (min)	Precursor ion (m/z)	Adduct Type	Calculated m/z	Mass Error (ppm)	Candidate Formula	Matched fragment count
Phenolic compounds	[6]-Gingerol	13447	14.634	295.18648	[M+H]+	295.19040	-13.2314	$C_{17}H_{26}O_4$	9
	Caffeoyl putrescin	12906	6.871	251.13520	[M+H]+	251.13900	-15.2462	$C_{13}H_{18}N_2O_3$	4
	Paeonol	12376	15.003	167.06817	[M+H]+	167.07027	-12.5734	$C_9H_{10}O_3$	4
	2-Hydroxycinnamic acid, predominantly trans	12292	1.769	165.05267	[M+H]+	165.05463	-11.8178	$C_9H_8O_3$	8
	6-Shogaol	9223	13.645	277.17450	[M+H]+	277.17999	-19.1973	$C_{17}H_{24}O_3$	2
	Hydrocinnamic acid	8999	6.508	151.07290	[M+H]+	151.07536	-16.2570	$C_9H_{10}O_2$	6
	Gallic acid	7554	6.549	171.02583	[M+H]+	171.02879	-17.3641	$C_7H_6O_5$	7
	3,4-Dihydroxymandelic acid	4971	6.549	185.04161	[M+H]+	185.04445	-15.3467	$C_8H_8O_5$	6
	Ferulic acid	3185	8.331	195.06321	[M+H]+	195.06519	-10.1262	$C_{10}H_{10}O_4$	8
Flavonoids	5,7-dihydroxy-2-(4-hydroxyphenyl)-6-[3,4,5-trihydroxy-6-(hydroxymethyl)oxan-2-yl]-8-(3,4,5-trihydroxyoxan-2-yl)chromen-4-one	29586	7.275	565.14416	[M+H]+	565.15521	-19.5027	$C_{26}H_{28}O_{14}$	44
	Tectochrysin	26488	1.19	269.07737	[M+H]+	269.08084	-12.8784	$C_{16}H_{12}O_4$	2
	Nepetin-7-glucoside	6684	11.438	479.13095	[M+H]+	479.11899	26.1885	$C_{22}H_{22}O_{12}$	3
	Flavone base + 4O, C-Pen, C-Hex	6605	6.952	581.13943	[M+H]+	581.15009	-18.3544	$C_{26}H_{28}O_{15}$	38
	Tectorigenin	5890	12.341	301.07101	[M+H]+	301.07001	1.1473	$C_{16}H_{12}O_6$	3
	Tricin	5854	11.029	331.07519	[M+H]+	331.08124	-18.2410	$C_{17}H_{14}O_7$	9
	5,7-dihydroxy-6-methoxy-2-(4-methoxyphenyl)-4H-chromen-4-one	3071	12.502	315.08387	[M+H]+	315.09000	-7.7586	$C_{17}H_{14}O_6$	3
Terpenoids / Steroids	Ceratodictyol	265166	23.471	331.27835	[M+H]+	331.28427	-17.9187	$C_{19}H_{38}O_4$	18

	22-Hydoxy-2-hopen-1-one	44579	20.064	441.36316	[M+H]+	441.37271	-21.6308	$C_{30}H_{48}O_2$	26
	Diosgenin	20005	25.823	415.32539	[M+H]+	415.32068	11.3607	$C_{27}H_{42}O_3$	1
	quassin	12388	5.364	389.19555	[M+H]+	389.20001	-0.8096	$C_{22}H_{28}O_6$	1
	Schisanhenol	9396	6.871	403.20842	[M+H]+	403.20999	-7.6762	$C_{23}H_{30}O_6$	1
	alpha-Santonin	5591	12.744	247.12695	[M+H]+	247.13287	-23.9584	$C_{15}H_{18}O_3$	3
Alkaloids	Trigonelline	166425	1.148	138.05433	[M+H]+	138.05496	-4.5267	$C_7H_7NO_2$	5
	2,8-Quinolinediol	59419	1.97	162.05380	[M+H]+	162.05496	-7.1268	$C_9H_7NO_2$	4
	Conessine	41842	17.897	357.31888	[M+H]+	357.32642	-21.1173	$C_{24}H_{40}N_2$	1
	Higenamine	41316	1.105	272.12929	[M+H]+	272.12000	4.2999	$C_{16}H_{17}NO_3$	2
	Piperidine	37085	1.93	86.09561	[M+H]+	86.09643	-9.4757	$C_5H_{11}N$	2
	Echimidine N-oxide	29673	14.088	414.20789	[M+H]+	414.21225	-10.5101	$C_{20}H_{31}NO_8$	1
	cinchonine	18872	13.645	295.18531	[M+H]+	295.17999	16.3297	$C_{19}H_{22}N_2O$	3
	Hydrocinchonine	10451	17.05	297.19812	[M+H]+	297.19614	6.6627	$C_{19}H_{24}N_2O$	2
	Intermedine	5990	7.194	300.17549	[M+H]+	300.18054	-16.8544	$C_{15}H_{25}NO_5$	1
	Indole-3-carboxyaldehyde	2336	5.617	146.05768	[M+H]+	146.06004	-16.1599	C_9H_7NO	4
Lipids	Lauryl diethanolamide	1939408	14.583	288.25103	[M+H]+	288.25333	-7.9458	$C_{16}H_{33}NO_3$	13
	Lauryl diethanolamide	609637	12.082	288.25085	[M+H]+	288.25333	-8.5702	$C_{16}H_{33}NO_3$	13
	1-Monostearin	582157	27.16	359.31051	[M+H]+	359.31558	-14.1277	$C_{21}H_{42}O_4$	9
	N-lauroylethanolamine	471784	15.003	244.22549	[M+H]+	244.22711	-6.6153	$C_{14}H_{29}NO_2$	7
	Stearamide	154564	26.253	284.29038	[M+H]+	284.29480	-15.5166	$C_{18}H_{37}NO$	5
	ISOPALMITIC ACID_major	101731	25.532	257.24356	[M+H]+	257.24750	-15.3422	$C_{16}H_{32}O_2$	64
	N-Dodecanoyl-N-methylglycine	62498	18.341	272.21835	[M+H]+	272.22202	-13.4826	$C_{15}H_{29}NO_3$	5
	monoolein	50785	23.641	357.29396	[M+H]+	357.29993	-16.7261	$C_{21}H_{40}O_4$	36
	N-C18:0 Phytoceramide	46166	25.002	584.54825	[M+H]+	584.56122	-22.2155	$C_{36}H_{73}NO_4$	9
	ELAIDIC ACID	37137	26.212	283.25729	[M+H]+	283.26315	-20.7115	$C_{18}H_{34}O_2$	47
	monolinolein	26922	21.533	355.27740	[M+H]+	355.28427	-19.3821	$C_{21}H_{38}O_4$	70

	Linoleic acid	25703	23.788	281.24320	[M+H]+	281.24750	-15.3130	$C_{18}H_{32}O_2$	71
	PC(16:0/18:1(9Z))	23185	23.641	760.56276	[M+H]+	760.58508	-29.3485	$C_{42}H_{82}NO_8P$	2
	D-sphingosine	22038	21.226	300.28481	[M+H]+	300.28970	-16.3039	$C_{18}H_{37}NO_2$	3
	LPC 18:2	15602	15.912	520.32900	[M+H]+	520.33980	-20.7488	$C_{26}H_{50}NO_7P$	8
	Palmitoleoyl Ethanolamide	10652	18.583	298.26999	[M+H]+	298.27405	-13.6312	$C_{18}H_{35}NO_2$	20
	Dehydrophytosphingosine (not validated, isomer of 1679)	9254	13.605	316.27880	[M+H]+	316.28460	-18.4344	$C_{18}H_{37}NO_3$	3
Amino acids and derivatives	L-Arginine	251352	1.022	175.11792	[M+H]+	175.11896	-5.8941	$C_6H_{14}N_4O_2$	10
	N-Fructosyl isoleucine	125660	1.89	294.15040	[M+H]+	294.15470	-14.5792	$C_{12}H_{23}NO_7$	24
	4-AMINOBUTANOATE	118195	1.022	104.07096	[M+H]+	104.07060	3.4111	$C_4H_9NO_2$	6
	Isoleucine	77607	1.93	132.10052	[M+H]+	132.10190	-10.4853	$C_6H_{13}NO_2$	2
	Proline	77325	17.332	116.06958	[M+H]+	116.07060	-8.8308	$C_5H_9NO_2$	2
	Pyroglutamic acid	76845	1.063	130.04894	[M+H]+	130.04987	-7.1477	$C_5H_7NO_3$	3
	L-(+)-Ergothioneine	52078	1.19	230.09338	[M+H]+	230.09578	-10.4060	$C_9H_{15}N_3O_2S$	13
	L-Glutamine	50040	1.063	147.07445	[M+H]+	147.07642	-13.3853	$C_5H_{10}N_2O_3$	4
	Lysine	49666	1.063	147.11071	[M+H]+	147.11281	-14.2351	$C_6H_{14}N_2O_2$	5
	Fructoselysine	46686	1.022	309.16008	[M+H]+	309.16562	-17.9437	$C_{12}H_{24}N_2O_7$	26
	5-OXO-L-PROLINE	43932	1.688	130.04856	[M+H]+	130.04987	-10.0696	$C_5H_7NO_3$	5
	Palmitoylcarnitine	42545	27.387	400.33576	[M+H]+	400.34213	-15.9249	$C_{23}H_{45}NO_4$	2
	Pantothenic acid	40370	1.19	220.11317	[M+H]+	220.11795	-21.7116	$C_9H_{17}NO_5$	3
	Glutamic acid	40188	1.063	148.05880	[M+H]+	148.06044	-11.0377	$C_5H_9NO_4$	5
	L-Tryptophan	38730	5.617	205.09303	[M+H]+	205.09715	-20.1080	$C_{11}H_{12}N_2O_2$	15
	L-Histidine	36400	0.98	156.07472	[M+H]+	156.07675	-13.0257	$C_6H_9N_3O_2$	4
	L-CARNITINE	33261	1.063	162.07414	[M+H]+	162.11247	-12.9528	$C_7H_{15}NO_3$	5
	N-Acetylhistidine	32242	1.022	198.09099	[M+H]+	198.08731	18.5388	$C_8H_{11}N_3O_3$	6
	Phenylalanine	29104	3.117	166.08400	[M+H]+	166.08630	-13.3970	$C_9H_{11}NO_2$	6

	N,N,N-TRIMETHYLLYSINE	27603	0.98	189.15752	[M+H]+	189.15976	-11.8120	$C_9H_{20}N_2O_2$	5
	Cocamidopropyl betaine	24283	12.179	343.28910	[M+H]+	343.29553	-18.6998	$C_{19}H_{38}N_2O_3$	2
	L(+)-Cystathionine	23503	1.022	223.07070	[M+H]+	223.07471	-17.9517	$C_7H_{14}N_2O_4S$	5
	N-Methyl-L-proline; CE0; CWLQUGTUXBXTLF-YFKPBYRVSA-N	17270	17.534	130.08473	[M+H]+	130.08626	-11.7235	$C_6H_{11}NO_2$	2
	Tyrosine	15088	1.769	182.07874	[M+H]+	182.08118	-13.3440	$C_9H_{11}NO_3$	12
	Pantothenate	13913	3.881	220.11423	[M+H]+	220.11795	-16.8960	$C_9H_{17}NO_5$	19
	Threonine	11636	1.063	120.06402	[M+H]+	120.06552	-12.4900	$C_4H_9NO_3$	6
	Serylleucine	7199	3.445	219.13116	[M+H]+	219.13393	-12.6568	$C_9H_{18}N_2O_4$	10
	C12-homoserine lactone	6565	13.766	284.21679	[M+H]+	284.22202	-18.4020	$C_{16}H_{29}NO_3$	5
	ValylIsoleucine	6276	5.737	231.16666	[M+H]+	231.17032	-15.8283	$C_{11}H_{22}N_2O_3$	3
	Alanine	4931	1.063	90.05386	[M+H]+	90.05495	-12.1585	$C_3H_7NO_2$	1
	Glycylleucine	4156	3.967	189.12187	[M+H]+	189.12337	-7.9252	$C_8H_{16}N_2O_3$	6
	C14-homoserine lactone	3819	13.228	312.24866	[M+H]+	312.25333	-14.9250	$C_{18}H_{33}NO_3$	10
	Threonylleucine	3192	3.886	233.14570	[M+H]+	233.14960	-16.7429	$C_{10}H_{20}N_2O_4$	7
Organic acids	4,5-dihydro-5,5-diphenyl-1,2-oxazole-3-carboxylic acid, ethyl ester	26601	1.148	296.12928	[M+H]+	296.12811	3.9176	$C_{18}H_{17}NO_3$	2
	4-Quinolinecarboxylic acid	2416	2.011	174.05245	[M+H]+	174.05496	-14.3916	$C_{10}H_7NO_2$	7
Nucleosides and nucleotides	(2R,3R,4S,5R)-2-(6-amino-9H-purin-9-yl)-5-(hydroxymethyl)tetrahydrofuran-3,4-diol	402722	1.728	268.10226	[M+H]+	268.10999	-6.6034	$C_{10}H_{13}N_5O_4$	6
	Adenine	82737	1.728	136.06084	[M+H]+	136.06177	-6.8473	$C_5H_5N_5$	3
	Guanosine	82297	1.93	284.09568	[M+H]+	284.09894	-11.4925	$C_{10}H_{13}N_5O_5$	3
	Guanine	75158	1.93	152.05543	[M+H]+	152.05669	-8.2618	$C_5H_5N_5O$	5
	URIDINE 5'-DIPHOSPHO-N-ACETYLGLUCOSAMINE	25727	1.358	608.07686	[M+H]+	608.08887	-19.7124	$C_{17}H_{27}N_3O_{17}P_2$	8
	Cytarabine	19970	1.148	244.08949	[M+H]+	244.09280	93.8291	$C_9H_{13}N_3O_5$	2
	2',2'-Difluoro-2'-deoxyuridine	18834	1.148	265.06335	[M+H]+	265.06305	1.1159	$C_9H_{10}F_2N_2O_5$	1
	Uridine	17156	1.688	245.07338	[M+H]+	245.07681	-14.0061	$C_9H_{12}N_2O_6$	3

	ADENOSINE 5'-MONOPHOSPHATE	16193	1.19	348.06437	[M+H]+	348.07037	-17.2128	$C_{10}H_{14}N_5O_7P$	3
	Uracil	10911	1.688	113.03326	[M+H]+	113.03455	-11.4464	$C_4H_4N_2O_2$	4
	5'-S-Methylthioadenosine	7476	5.576	298.09129	[M+H]+	298.09683	-18.6081	$C_{11}H_{15}N_5O_3S$	3
	2-O-Methyladenosine	6520	2.541	282.11440	[M+H]+	282.11969	-18.7171	$C_{11}H_{15}N_5O_4$	2
	Adenylosuccinate	4444	2.259	464.07391	[M+H]+	464.08133	-15.9668	$C_{14}H_{18}N_5O_{11}P$	7
	Guanosine-3',5'-cyclic monophosphate	2889	1.728	346.04753	[M+H]+	346.05472	-20.7517	$C_{10}H_{12}N_5O_7P$	3
Carbohydrates	D-Sorbitol	50438	1.105	183.08382	[M+H]+	183.08632	-13.6255	$C_6H_{14}O_6$	14
	ALPHA-D-GLUCOSE 1-PHOSPHATE	27954	1.148	261.03280	[M+H]+	261.03699	-16.0723	$C_6H_{13}O_9P$	10
	1,5-Anhydrosorbitol	13156	1.105	165.07272	[M+H]+	165.07574	-18.3549	$C_6H_{12}O_5$	2
	N-Acetylglucosamine	3479	2.173	222.09322	[M+H]+	222.09721	-17.9816	$C_8H_{15}NO_6$	18
Peptides	Leucylproline	7051	5.53	229.15120	[M+H]+	229.15466	-15.1382	$C_{11}H_{20}N_2O_3$	5
	Cyclo(proline-leucine)	6589	7.81	211.13948	[M+H]+	211.14410	-21.9011	$C_{11}H_{18}N_2O_2$	7
	PyroglutamylIsoleucine	5791	7.235	243.12950	[M+H]+	243.13390	-18.4406	$C_{11}H_{18}N_2O_4$	9
	Leucylvaline	4751	5.116	231.16645	[M+H]+	231.17032	-16.7368	$C_{11}H_{22}N_2O_3$	6
	Ala-Phe	3686	5.53	237.12081	[M+H]+	237.12337	-10.7912	$C_{12}H_{16}N_2O_3$	6
Other natural products	Eudesmin	122345	14.249	387.18801	[M+H]+	387.17999	20.1327	$C_{22}H_{26}O_6$	3
	Zearalenone	31021	1.022	319.15553	[M+H]+	319.15399	4.7931	$C_{18}H_{22}O_5$	3
Environmental contaminants / Synthetic compounds	Tris(2-butoxyethyl) phosphate	1583850	18.3	399.24598	[M+H]+	399.25061	-11.6144	$C_{18}H_{39}O_7P$	10
	Di-n-butyl phthalate	350390	19.129	279.15555	[M+H]+	279.15909	-12.6654	$C_{16}H_{22}O_4$	4
	Triphenylphosphine oxide	240792	12.341	279.09114	[M+H]+	279.09332	-7.8417	$C_{18}H_{15}OP$	3
	Pesticide3_Cycluron_C11H22N2O_3-Cyclooctyl-1,1-dimethylurea	58033	21.041	199.18352	[M+H]+	199.18050	15.2133	$C_{11}H_{22}N_2O$	5
	Flumequine	28448	1.231	262.09229	[M+H]+	262.08740	18.6660	$C_{14}H_{12}FNO_3$	2

	Pramoxine	26419	18.341	294.19911	[M+H]+	294.20636	-24.6772	$C_{17}H_{27}NO_3$	1
	Desethylatrazine	24592	5.617	188.06851	[M+H]+	188.06975	-6.5906	$C_6H_{10}ClN_5$	1
	Dibucaine	24113	17.655	344.23767	[M+H]+	344.23325	12.8295	$C_{20}H_{29}N_3O_2$	7
	Tris(1-chloro-2-propyl)phosphate	23470	13.887	327.00286	[M+H]+	327.00812	-16.0411	$C_9H_{18}Cl_3O_4P$	4
	Acetyl tributyl citrate	22593	21.186	403.22424	[M+H]+	403.23264	-20.8429	$C_{20}H_{34}O_8$	7
	Methoxyfenozide	18154	12.905	369.22048	[M+H]+	369.21725	8.6961	$C_{22}H_{28}N_2O_3$	1
	Betaxolol	12226	18.462	308.21593	[M+H]+	308.22202	-19.7593	$C_{18}H_{29}NO_3$	1
	Tris(2-chloroethyl)phosphate	10750	11.438	284.95720	[M+H]+	284.96115	-13.8804	$C_6H_{12}Cl_3O_4P$	7
	Triphenylphosphate	10357	17.09	327.07268	[M+H]+	327.07806	-16.4867	$C_{18}H_{15}O_4P$	13
	Denatonium	7701	17.615	326.22655	[M+H]+	326.23526	-26.7139	$C_{21}H_{29}N_2O$	2
	Benzyl-butyl-phthalate	7083	18.766	313.13686	[M+H]+	313.14343	-20.9987	$C_{19}H_{20}O_4$	3
	N4-Acetylsulfadimethoxine	6974	1.646	353.09800	[M+H]+	353.09143	18.6431	$C_{14}H_{16}N_4O_5S$	1
	Tebuconazole	5458	7.033	308.14831	[M+H]+	308.15240	-13.3262	$C_{16}H_{22}ClN_3O$	1
Putative / Unknown compounds	(1S,3R,6S,6aR,6bR,8S,9S,11R,11aR,12R,12aR,14R)-1-ethyl-6,8,11-trihydroxy-3-methyl-10-methylenetetradecahydro-3,6a,12-(epiethane[1,1,2]triyl)-9,11a-methanoazuleno[2,1-b]azocine 1-oxide	1113652	14.088	376.25562	[M+H]+	376.25000	19.6280	$C_{22}H_{33}NO_4$	1
	Erucamide	604022	30.826	338.33683	[M+H]+	338.34174	-14.5163	$C_{22}H_{43}NO$	63
	Laurocapram	408815	23.389	282.27573	[M+H]+	282.27914	-12.0846	$C_{18}H_{35}NO$	14
	Lauryldiethanolamine	372980	11.639	274.27194	[M+H]+	274.27405	-7.7143	$C_{16}H_{35}NO_2$	4
	Phthalic anhydride	272022	19.129	149.02213	[M+H]+	149.02332	-7.9884	$C_8H_4O_3$	3
	Tetradecyldiethanolamine	122664	13.066	302.30104	[M+H]+	302.30536	-14.2768	$C_{18}H_{39}NO_2$	4
	Diphenylsulfone	88238	12.623	219.04507	[M+H]+	219.04742	-10.7612	$C_{12}H_{10}O_2S$	12
	Chasmanine	85205	17.776	452.31356	[M+H]+	452.30099	28.5109	$C_{25}H_{41}NO_6$	1
	Benzyldiphenylphosphine oxide	74041	12.502	293.10544	[M+H]+	293.10898	-12.0728	$C_{19}H_{17}OP$	4
	(2-(4,4-dimethyl-4,5-dihydrooxazol-2-yl)-5-	71724	1.728	300.14026	[M+H]+	300.13943	2.7539	$C_{18}H_{18}FNO_2$	2

fluorophenyl)(phenyl)methanol								
NCGC00384699-01!(2E,6E,10E)-3,7,11,15,19,23,27,31,35-nonamethylhexatriaconta-2,6,10,34-tetraene-1,15,19,23,27,31-hexol	58227	31.827	721.63445	[M+H]+	721.63409	0.5309	$C_{45}H_{84}O_6$	86
3,6,9,12-Tetraoxatetracosan-1-ol	48098	20.96	363.30387	[M+H]+	363.31049	-18.2514	$C_{20}H_{42}O_5$	4
5-O-methylvisammioside	44901	14.542	453.15864	[M+H]+	453.17001	-37.2561	$C_{22}H_{28}O_{10}$	1
Lactose	36895	1.105	343.11673	[M+H]+	343.12347	-19.6955	$C_{12}H_{22}O_{11}$	15
2,6-Xylidine	35622	4.346	122.09466	[M+H]+	122.09643	-14.4625	$C_8H_{11}N$	6
Scleroderolide	34103	1.105	329.09714	[M+H]+	329.10196	-14.6602	$C_{18}H_{16}O_6$	1
ethyl 3-(2,4-dihydroxy-6-methylpyridin-3-yl)-3-(3-hydroxyphenyl)propanoate	33430	1.646	318.12520	[M+H]+	318.13000	-26.4014	$C_{17}H_{19}NO_5$	4
Mycosporine serinol	27048	1.231	262.12464	[M+H]+	262.12851	-14.7783	$C_{11}H_{19}NO_6$	7
NCGC00015088-09_C12H14N2O_N-[2-(1H-Indol-3-yl)ethyl]acetamide	26067	9.32	203.11556	[M+H]+	203.11789	-11.4689	$C_{12}H_{14}N_2O$	12
(E)-4-(4-methoxybenzylidene)-3-phenylisoxazol-5(4H)-one	25068	1.231	280.09774	[M+H]+	280.10001	3.2855	$C_{17}H_{13}NO_3$	2
GLYCEROL 2-PHOSPHATE	23724	1.148	173.01836	[M+H]+	173.02095	-14.9778	$C_3H_9O_6P$	3
NCGC00347722-02!(2Z)-2-[(E)-6-(hydroxymethyl)-2,4,8,10-tetramethyldodec-2-enylidene]-4-methylpentanedioic acid [IIN-based: Match]	23542	19.983	397.28849	[M+H]+	397.29486	-16.0104	$C_{23}H_{40}O_5$	31
L-Saccharopine	23362	1.063	277.13527	[M+H]+	277.13940	-14.9486	$C_{11}H_{20}N_2O_6$	7
Putative (3-hydroxyhexadecanoyl)glycine (aka Commendamide)	22531	12.663	330.25814	[M+H]+	330.26389	-17.3954	$C_{18}H_{35}NO_4$	8
bilobol	20107	31.691	319.25481	[M+H]+	319.26315	-26.1440	$C_{21}H_{34}O_2$	10
Andrachcinidine	19898	12.219	228.19259	[M+H]+	228.19580	-14.0910	$C_{13}H_{25}NO_2$	10
3,4-dimethyl-9-phenyl-7H-furo[2,3-f]chromen-7-one	19519	1.646	291.09435	[M+H]+	291.10001	-24.8050	$C_{19}H_{14}O_3$	3
Dicyclohexylurea	19404	12.704	225.19331	[M+H]+	225.19614	-12.5662	$C_{13}H_{24}N_2O$	4
GLUCOSAMINATE	19096	1.063	196.07865	[M+H]+	196.08156	-14.8592	$C_6H_{13}NO_6$	25
L-Aspartic acid; CE0; CKLJMWTZIZZHCS-REOHCLBHSA-N	19044	1.063	134.04282	[M+H]+	134.04478	-14.6531	$C_4H_7NO_4$	4

2-Naphthylamine	18422	9.32	144.07934	[M+H]+	144.08078	-9.9650	$C_{10}H_9N$	7
Coumaroyl agmatine	17549	17.937	277.17440	[M+H]+	277.16590	30.6590	$C_{14}H_{20}N_4O_2$	3
(2S)-2-((1S)-1-Hydroxypentyl)-4-methoxy-2,3-dihydropyran-6-one	17480	9.867	215.12433	[M+H]+	215.12779	-16.0627	$C_{11}H_{18}O_4$	16
3-HYDROXY-4-(SUCCIN-2-YL)-CARYOLANE delta-LACTONE	17288	21.626	321.20143	[M+H]+	321.20602	-14.3392	$C_{19}H_{28}O_4$	27
2,2'-(Oxydimethanediyl)bis(2-ethylpropane-1,3-diol)	17132	8.159	251.18136	[M+H]+	251.18530	-15.6872	$C_{12}H_{26}O_5$	9
Fenpropimorph	16963	23.389	304.25451	[M+H]+	304.26349	-29.5177	$C_{20}H_{33}NO$	1
Tyramine	16887	1.809	138.08923	[M+H]+	138.09134	-15.2830	$C_8H_{11}NO$	6
5-(3-methoxyphenyl)-N-((octahydro-1H-quinolizin-1-yl)methyl)-1H-pyrazole-3-carboxamide	16345	23.471	369.23564	[M+H]+	369.23001	19.3305	$C_{21}H_{28}N_4O_2$	1
Dicyclohexylamine	16292	7.931	182.18817	[M+H]+	182.19032	-11.8349	$C_{12}H_{23}N$	4
DIETHANOLAMINE	15999	14.583	106.08517	[M+H]+	106.08626	-10.2281	$C_4H_{11}NO_2$	2
heteratisine	15797	16.2	392.24649	[M+H]+	392.23999	8.5161	$C_{22}H_{33}NO_5$	1
phenylethylamide 359	14933	23.348	360.32323	[M+H]+	360.32608	-7.9412	$C_{24}H_{41}NO$	6
(2'R,5'R,9S)-4-hydroxy-5',6a,8a,9-tetramethylicosahydrospiro[naphtho[2',1':4,5]indeno[2,1-b]furan-10,2'-pyran]-2(11aH)-one	14868	14.329	431.30228	[M+H]+	431.31000	-30.8505	$C_{27}H_{42}O_4$	2
chaulmoogric acid	14823	20.497	281.24324	[M+H]+	281.25000	-15.1708	$C_{18}H_{32}O_2$	2
Hydroxybutorphanol	14643	6.508	344.22186	[M+H]+	344.22202	-0.4656	$C_{21}H_{29}NO_3$	2
Lauric isopropanolamide	14386	16.458	258.23828	[M+H]+	258.24277	-17.3314	$C_{15}H_{31}NO_2$	13
6-(4-(2,3-dihydrobenzo[b][1,4]dioxin-6-yl)-3-methyl-1H-pyrazol-5-yl)-2,3-dihydrobenzo[b][1,4]dioxin-5-ol	13985	22.202	367.12729	[M+H]+	367.13000	-4.2442	$C_{20}H_{18}N_2O_5$	2
Ophthalmic acid; CE0; JCMUOFQHZLPHQP-BQBZGAKWSA-N	12197	1.646	290.13239	[M+H]+	290.13464	-7.8302	$C_{11}H_{19}N_3O_6$	3
delta-Undecalactone	11877	11.558	185.15020	[M+H]+	185.15361	-18.3974	$C_{11}H_{20}O_2$	15
Bis[2-(2-butoxyethoxy)ethyl] hexandioate	11793	17.776	435.28746	[M+H]+	435.29526	-17.8839	$C_{22}H_{42}O_8$	15
Dihydrojasmone	11775	11.558	167.14042	[M+H]+	167.14304	-15.6852	$C_{11}H_{18}O$	8
C17H33N3O4	11739	7.638	344.24640	[M+H]+	344.25440	-23.1603	$C_{17}H_{33}N_3O_4$	3

NCGC00380833-01_C20H28O5_Gibbane-1,10-dicarboxylic acid, 2-hydroxy-1,4a-dimethyl-8-methylene-, (1alpha,2beta,4aalpha,4bbeta,10beta)-	11601	8.952	349.19537	[M+H]+	349.20096	-15.9807	$C_{20}H_{28}O_5$	40
(E)-5-hydroxy-3-isobutyl-6-(3-methylbenzylidene)-1,6-dihydropyrazin-2(3H)-one	11449	8.2	273.16238	[M+H]+	273.16000	9.6121	$C_{16}H_{20}N_2O_2$	2
Corynoxeine	11318	19.17	383.19433	[M+H]+	383.19000	-5.7511	$C_{22}H_{26}N_2O_4$	1
2,5-Dichlorophenol	11183	32.117	162.97132	[M+H]+	162.97119	0.7573	$C_6H_4Cl_2O$	3
6-Hydroxynicotinic acid	11102	2.34	140.03197	[M+H]+	140.03423	-16.0639	$C_6H_5NO_3$	7
(NAG)(MUR)-None	10646	1.646	455.17795	[M+H]+	455.18716	-20.2134	$C_{17}H_{30}N_2O_{12}$	4
2,3-Phenazinediamine	10644	7.033	211.09460	[M+H]+	211.09782	-15.2669	$C_{12}H_{10}N_4$	6
Acitretin	10169	6.508	327.19568	[M+H]+	327.20001	0.6383	$C_{21}H_{26}O_3$	3
Tryptamine	10139	6.14	161.10473	[M+H]+	161.10733	-16.1064	$C_{10}H_{12}N_2$	11
4-((9S)-3,5,14-trihydroxy-10-((E)-((1-hydroxybutan-2-yl)imino)methyl)-13-methylhexadecahydro-1H-cyclopenta[a]phenanthren-17-yl)furan-2(5H)-one	9916	7.315	476.29662	[M+H]+	476.29999	-8.4915	$C_{27}H_{41}NO_6$	2
Flunisolide	9908	16.889	435.20548	[M+H]+	435.21774	-28.1775	$C_{24}H_{31}FO_6$	3
N,N-Dimethyltetradecylamine-N-oxide	9650	13.187	258.27451	[M+H]+	258.27914	-17.9311	$C_{16}H_{35}NO$	2
N-Acetyl-5-hydroxytryptamine	9489	7.194	219.10906	[M+H]+	219.11281	-17.0878	$C_{12}H_{14}N_2O_2$	11
N,N-Dimethyldecylamine oxide	9473	10.402	202.21386	[M+H]+	202.21654	-13.2579	$C_{12}H_{27}NO$	4
4-epivulgarin	9399	12.784	265.13633	[M+H]+	265.14001	-26.7990	$C_{15}H_{20}O_4$	4
Viridicatin	8591	1.646	238.08760	[M+H]+	238.08626	5.6489	$C_{15}H_{11}NO_2$	3
Xanthurenic acid	8546	4.058	206.04133	[M+H]+	206.04478	-16.7642	$C_{10}H_7NO_4$	4
(2E)-3-(4-hydroxy-3-methoxyphenyl)-2-propenoic acid	8306	2.42	195.07533	[M+H]+	195.07001	52.0069	$C_{10}H_{10}O_4$	4
Pyrrolidine; CE10; RWRDLPDLKQPQOW-UHFFFAOYSA-N	8056	1.605	72.08010	[M+H]+	72.08078	-9.3751	C_4H_9N	2
NCGC00381166-01_C15H26O3_	8039	13.847	255.19053	[M+H]+	255.19547	-19.3623	$C_{15}H_{26}O_3$	41
Leupeptin	7728	14.836	427.31118	[M+H]+	427.30273	19.7747	$C_{20}H_{38}N_6O_4$	2
Gabexate	7602	11.478	322.17970	[M+H]+	322.17615	11.0725	$C_{16}H_{23}N_3O_4$	8

(4R)-3-methylidene-4-[(E)-3-methyl-4-(4-methyl-5-oxooxolan-2-yl)but-2-enyl]oxolan-2-one	7069	7.235	265.13934	[M+H]+	265.14343	-15.4467	$C_{15}H_{20}O_4$	24
NCGC00347651-02_C20H26O3_1-Phenanthrenecarboxylic acid, 1,2,3,4,4a,9,10,10a-octahydro-1,4a-dimethyl-7-(1-methylethyl)-9-oxo-	6854	16.59	315.19120	[M+H]+	315.19547	-13.5508	$C_{20}H_{26}O_3$	40
T-2 tetraol	6835	8.614	299.14409	[M+H]+	299.14893	-16.1287	$C_{15}H_{22}O_6$	18
NCGC00169318-02!(E)-7-(4-methoxy-5-methyl-6-oxopyran-2-yl)-5-methyloct-6-enoic acid [IIN-based: Match]	6509	8.331	295.14919	[M+H]+	295.15399	-16.2975	$C_{16}H_{22}O_5$	35
N-(2,4-dimethylphenyl)formamide	6390	7.396	150.08886	[M+H]+	150.09134	-16.5262	$C_9H_{11}NO$	6
allo-Protolichesterinic acid	5913	16.585	325.23146	[M+H]+	325.23734	-18.0667	$C_{19}H_{32}O_4$	55
Ritalinic acid	5832	10.781	220.12939	[M+H]+	220.13321	-17.3316	$C_{13}H_{17}NO_2$	2
2-(2-methoxy-phenyl)-5-oxo-tetrahydro-furan-3-carboyl-lupinine	5539	1.688	388.20921	[M+H]+	388.20999	-6.7991	$C_{22}H_{29}NO_5$	2
NCGC00169423-02_C20H28O6_(2aS,5aR,7R,8aR)-6-Hydroxy-6-[2-(2-hydroxy-5-oxo-2,5-dihydro-3-furanyl)ethyl]-2a,5a,7-trimethyldecahydro-2H-naphtho[1,8-bc]furan-2-one	5293	9.867	365.18898	[M+H]+	365.19586	-18.8531	$C_{20}H_{28}O_6$	65
(2S,2'S)-3,3'-disulfanediylbis(2-aminopropanoic acid)	5260	12.663	241.02650	[M+H]+	241.03000	-19.1913	$C_6H_{12}N_2O_4S_2$	4
2-[4-(Diethylamino)-2-hydroxybenzoyl]benzoic acid	5090	2.784	314.14738	[M+H]+	314.13867	27.6803	$C_{18}H_{19}NO_4$	3
12-epinapelline	4974	8.372	360.24366	[M+H]+	360.25000	-26.8156	$C_{22}H_{33}NO_3$	2
Dihydropalmatine	4933	3.238	354.16975	[M+H]+	354.17001	-0.6626	$C_{21}H_{23}NO_4$	1
(R)-4-aminoisoxazolidin-3-one	4876	4.306	103.05275	[M+H]+	103.05000	24.7073	$C_3H_6N_2O_2$	3
L-Asarinin	4746	9.62	355.10999	[M+H]+	355.12000	-21.4711	$C_{20}H_{18}O_6$	1
Albendazole Sulfone	4735	2.173	298.08693	[M+H]+	298.08560	4.4497	$C_{12}H_{15}N_3O_4S$	4
Isotalatizidine	4168	16.768	408.26585	[M+H]+	408.27444	-21.0637	$C_{23}H_{37}NO_5$	2
(S)-3-amino-4-(((S)-1-methoxy-1-oxo-3-phenylpropan-2-yl)amino)-4-oxobutanoic acid	4098	3.405	295.12425	[M+H]+	295.13000	-15.5802	$C_{14}H_{18}N_2O_5$	2
Olivetonide	4087	8.079	249.10716	[M+H]+	249.11214	-19.9728	$C_{14}H_{16}O_4$	13
N-(2-Phenylethyl)acetamide	4012	8.947	164.10491	[M+H]+	164.10699	-12.6778	$C_{10}H_{13}NO$	5

	3-Amino-Beta-Pinene	3815	7.557	188.12518	[M+H]+	188.12006	27.2500	$C_{10}H_{18}ClN$	16
	3-(2-hydroxy-4-methoxyphenyl)-3,4-dihydro-2H-1-benzopyran-7-ol	3804	12.042	273.10695	[M+H]+	273.10999	-18.9866	$C_{16}H_{16}O_4$	4
	2-(hydroxymethyl)-5-methoxy-4H-pyran-4-one	3751	2.703	157.04655	[M+H]+	157.05000	-19.0080	$C_7H_8O_4$	11
	C13H23NO5	3363	3.756	274.17110	[M+H]+	274.16490	22.4707	$C_{13}H_{23}NO_5$	4
	Olaquindox	2899	5.076	264.08352	[M+H]+	264.09000	-54.3828	$C_{12}H_{13}N_3O_4$	1
	Kynurenic acid	2608	4.956	190.04739	[M+H]+	190.04987	-13.0469	$C_{10}H_7NO_3$	5
	NCGC00380712-01_C19H18O6_1,3-Cyclobutanedicarboxylic acid, 2,4-bis(4-hydroxyphenyl)-, monomethyl ester	2488	9.747	343.11131	[M+H]+	343.11761	-18.3749	$C_{19}H_{18}O_6$	50
	Methoxetamine	2464	9.32	248.17177	[M+H]+	248.16451	29.2734	$C_{15}H_{21}NO_2$	3
	Imazamox	2079	4.654	306.13937	[M+H]+	306.14484	-17.8431	$C_{15}H_{19}N_3O_4$	1
	NCGC00381155-01_C20H18O8_2-Furanacetic acid, 3-(3,4'-dihydroxy-2,5-dimethoxy[1,1'-biphenyl]-4-yl)-2,5-dihydro-5-oxo-	1966	8.625	387.10021	[M+H]+	387.10745	-18.6873	$C_{20}H_{18}O_8$	39

Note. Compound names are reported as putative LC–MS/MS annotations. All listed analytical features were acquired with MS/MS data; however, feature-level product-ion spectra, individual similarity scores, and matched-fragment counts were not traceable for every entry in the archived summary output. Therefore, the annotations should not be interpreted as confirmed compound identities. Authentic reference standards were not analyzed. The adduct setting was $[M+H]^+$ for positive-ion mode.

Table S2. Putatively annotated LC-ESI(−)-MS/MS features retained after final screening.

**Chemical class**	**Putative annotation**	**Peak area**	**RT (min)**	**Precursor ion (m/z)**	**Adduct Type**	**Calculated m/z**	**Mass Error (ppm)**	**Candidate Formula**	**Matched fragment count**
Phenolic compounds	3-Hydroxymandelic acid	21020	2.399	167.03503	[M-H]-	167.03499	0.2858	$C_8H_8O_4$	2
	(R)-(-)-mandelic acid	14004	6.411	151.04032	[M-H]-	151.04007	1.6707	$C_8H_8O_3$	2
	Sinapic acid	10234	8.281	223.06114	[M-H]-	223.06120	-0.2556	$C_{11}H_{12}O_5$	11
	Octyl gallate	9962	7.18	281.13972	[M-H]-	281.13943	0.9698	$C_{15}H_{22}O_5$	1

	Mycophenolic acid	9077	1.875	319.11208	[M-H]-	319.11871	-20.7819	$C_{17}H_{20}O_6$	1
	Ferulate	7244	8.281	193.05104	[M-H]-	193.05063	2.1117	$C_{10}H_{10}O_4$	9
	3-Hydroxybenzaldehyde	7124	7.341	121.02967	[M-H]-	121.02950	1.3801	$C_7H_6O_2$	5
	Salicylic acid	5326	6.452	136.99158	[M-H]-	137.02441	0.4555	$C_7H_6O_3$	3
	trans-Ferulic acid	4339	9.859	193.05100	[M-H]-	193.05060	2.0599	$C_{10}H_{10}O_4$	3
	Octyl gallate	3293	6.853	281.13988	[M-H]-	281.13943	1.5390	$C_{15}H_{22}O_5$	1
	P-Coumaric acid	1858	7.994	163.03971	[M-H]-	163.04007	-2.1936	$C_9H_8O_3$	2
	3,4-Dihydroxybenzoic acid	839	8.562	153.02017	[M-H]-	153.01933	5.4751	$C_7H_6O_4$	3
Flavonoids	8-[(2S,3R,4R,5S,6R)-3,4-dihydroxy-6-(hydroxymethyl)-5-[(2S,3R,4S,5S,6R)-3,4,5-trihydroxy-6-(hydroxymethyl)oxan-2-yl]oxyoxan-2-yl]-5,7-dihydroxy-2-(4-hydroxyphenyl)chromen-4-one	11766	7.421	593.15329	[M-H]-	593.15118	3.5340	$C_{27}H_{30}O_{15}$	3
	Flavone base + 4O, C-Pen, C-Hex	10138	6.933	579.13440	[M-H]-	579.13550	-2.0094	$C_{26}H_{28}O_{15}$	9
	Isorhamnetin	5885	11.183	315.05144	[M-H]-	315.05103	1.3133	$C_{16}H_{12}O_7$	4
	7-O-beta-glucopyranosyl-4'-hydroxy-5-methoxyisoflavone	3434	8.195	445.11273	[M-H]-	445.11401	-2.8991	$C_{22}H_{22}O_{10}$	5
	isokaempferide	2729	11.063	299.05703	[M-H]-	299.05612	3.0709	$C_{16}H_{12}O_6$	5
	Quercetin-3-O-rhamnoside	2398	13.74	447.08145	[M-H]-	447.09329	-26.4710	$C_{21}H_{20}O_{11}$	2
	3',4',5,7-tetrahydroxyflavone	1647	10.02	285.04052	[M-H]-	285.04047	0.2050	$C_{15}H_{10}O_6$	3
Terpenoids / Steroids	aurapten	17912	10.701	297.14781	[M-H]-	297.14963	-6.0848	$C_{19}H_{22}O_3$	4
Alkaloids	2,8-Quinolinediol	16473	1.956	160.04036	[M-H]-	160.04041	-0.2624	$C_9H_7NO_2$	5
	2,8-Quinolinediol	6186	4.921	160.04112	[M-H]-	160.04041	4.4864	$C_9H_7NO_2$	6
	Isorhynchophylline	4120	6.893	383.20871	[M-H]-	383.19763	28.9122	$C_{22}H_{28}N_2O_4$	2

	2,8-Quinolinediol	3443	4.021	160.03929	[M-H]-	160.04041	-6.9483	$C_9H_7NO_2$	6
	3-Hydroxypyridine	1794	2.319	94.02972	[M-H]-	94.02984	-1.2477	C_5H_5NO	2
Lipids	FA 18:3+2O	31635	13.539	309.20709	[M-H]-	309.20712	-0.1390	$C_{18}H_{30}O_4$	5
	FA 18:2+2O	16938	14.714	311.22360	[M-H]-	311.22278	2.6250	$C_{18}H_{32}O_4$	4
	Linoleic acid	15782	23.546	279.23406	[M-H]-	279.23294	3.9616	$C_{18}H_{32}O_2$	3
	9,10-DiHOME	15470	14.674	313.23719	[M-H]-	313.23843	-3.9686	$C_{18}H_{34}O_4$	10
	9-HODE	11652	17.266	295.22733	[M-H]-	295.22787	-1.8238	$C_{18}H_{32}O_3$	3
	LPE 18:2	8788	15.401	476.27660	[M-H]-	476.27830	-3.4923	$C_{23}H_{44}NO_7P$	4
	12-HYDROXYDODECANOIC ACID	7922	11.951	215.16593	[M-H]-	215.16527	3.0759	$C_{12}H_{24}O_3$	4
	Dodecanoic acid/Lauric acid candidate	7512	12.775	199.17080	[M-H]-	199.17040	2.1914	$C_{12}H_{24}O_2$	2
	12-HYDROXYDODECANOIC ACID	6315	15.446	215.16549	[M-H]-	215.16527	1.0310	$C_{12}H_{24}O_3$	4
	FA 18:1+3O	3775	11.905	329.23393	[M-H]-	329.23334	1.7686	$C_{18}H_{34}O_5$	3
	Dodecanoic acid/Lauric acid candidate	3233	15.873	199.16940	[M-H]-	199.17040	-4.5868	$C_{12}H_{24}O_2$	1
	FA 9:2+1O	1418	7.501	169.08715	[M-H]-	169.08702	0.7816	$C_9H_{14}O_3$	2
Amino acids and derivatives	Pyroglutamic acid	67571	1.672	128.03584	[M-H]-	128.03532	4.0878	$C_5H_7NO_3$	2
	Arginine	39978	1	173.10473	[M-H]-	173.10440	1.9107	$C_6H_{14}N_4O_2$	2
	Glutamine	38153	1.042	145.06180	[M-H]-	145.06186	-0.4531	$C_5H_{10}N_2O_3$	6
	N-Fructosyl isoleucine	35155	1.875	292.13885	[M-H]-	292.14017	-4.5376	$C_{12}H_{23}NO_7$	5
	Ornithine	29106	1	131.08249	[M-H]-	131.08260	-0.8481	$C_5H_{12}N_2O_2$	4
	L-Glutamic acid	26470	1.083	146.04619	[M-H]-	146.04588	2.1137	$C_5H_9NO_4$	4
	Tryptophan	21470	5.592	203.08285	[M-H]-	203.08260	1.2253	$C_{11}H_{12}N_2O_2$	7
	L-(-)-Phenylalanine	19183	3.044	164.07160	[M-H]-	164.07170	-0.6225	$C_9H_{11}NO_2$	5

	D-Pantothenic acid	18977	3.85	218.10410	[M-H]-	218.10339	3.2270	$C_9H_{17}NO_5$	5
	tyrosine	15539	1.753	180.06724	[M-H]-	180.06662	3.4612	$C_9H_{11}NO_3$	8
	Histidine	15017	1	154.06198	[M-H]-	154.06219	-1.4285	$C_6H_9N_3O_2$	6
	Threonine	8246	1.083	118.05107	[M-H]-	118.05096	0.8752	$C_4H_9NO_3$	2
	Lauroylcarnitine	6397	16.295	342.26503	[M-H]-	342.26498	0.1396	$C_{19}H_{37}NO_4$	2
	L-Ethionine	5950	4.921	162.05568	[M-H]-	162.05942	-23.0988	$C_6H_{13}NO_2S$	3
	Serine	4781	1.042	104.03532	[M-H]-	104.03532	0.0325	$C_3H_7NO_3$	3
	Methoxytyrosine	4221	6.974	210.07827	[M-H]-	210.07718	5.1817	$C_{10}H_{13}NO_4$	2
	3-oxo-C4-homoserine lactone	2492	4.961	184.06241	[M-H]-	184.06154	4.7736	$C_8H_{11}NO_4$	3
	3-hydroxy-C6-homoserine lactone	1542	5.138	214.10824	[M-H]-	214.10847	-1.1282	$C_{10}H_{17}NO_4$	4
	N-acetyl-O-methyltyrosine	1188	8.281	236.09352	[M-H]-	236.09283	2.9162	$C_{12}H_{15}NO_4$	5
Organic acids	D-(+)-Malic acid	164591	1.308	133.01501	[M-H]-	133.01425	5.7375	$C_4H_6O_5$	5
	Citric acid	90525	1.63	191.02080	[M-H]-	191.01973	5.6218	$C_6H_8O_7$	7
	Fumaric acid	43427	1.308	115.00390	[M-H]-	115.00370	2.0683	$C_4H_4O_4$	2
	Threonic acid	24756	1.13	135.03037	[M-H]-	135.02989	3.5037	$C_4H_8O_5$	10
	Azelaic acid	22864	8.898	187.09803	[M-H]-	187.09758	2.3917	$C_9H_{16}O_4$	7
	DL-Lactic acid	20145	1.63	89.02452	[M-H]-	89.02441	1.1504	$C_3H_6O_3$	1
	Oxalic acid	19290	1.63	88.98818	[M-H]-	88.98803	1.6623	$C_2H_2O_4$	3
	D-sorbosonic acid	18323	1.13	193.03597	[M-H]-	193.03537	3.0762	$C_6H_{10}O_7$	5
	SUBERIC ACID	10651	7.874	173.08196	[M-H]-	173.08194	0.1591	$C_8H_{14}O_4$	5
	Citraconic acid	7515	2.118	129.01957	[M-H]-	129.01933	1.8431	$C_5H_6O_4$	2
	Sebacic acid	6844	9.899	201.11420	[M-H]-	201.11320	4.8103	$C_{10}H_{18}O_4$	2
	Undecanedioic acid	5413	10.822	215.12950	[M-H]-	215.12888	2.8696	$C_{11}H_{20}O_4$	4

	Tridecanedioic acid	4752	12.494	243.16115	[M-H]-	243.16019	3.9777	$C_{13}H_{24}O_4$	4
	Dodecanedioic acid	4524	11.678	229.14405	[M-H]-	229.14453	-2.1066	$C_{12}H_{22}O_4$	4
	3-Methyladipic acid	4141	6.693	159.06662	[M-H]-	159.06628	2.1224	$C_7H_{12}O_4$	3
	DL-α-Hydroxyglutaric acid	3812	2.118	147.03040	[M-H]-	147.02990	3.2858	$C_5H_8O_5$	2
	Dodecanedioic acid	3770	9.024	229.14381	[M-H]-	229.14453	-3.1540	$C_{12}H_{22}O_4$	4
	Succinic acid	3728	1.915	117.01968	[M-H]-	117.01933	2.9721	$C_4H_6O_4$	5
	Octadecanedioic acid	2532	12.237	313.23710	[M-H]-	313.23843	-4.2559	$C_{18}H_{34}O_4$	2
	Glutaric acid	2060	3.727	131.03473	[M-H]-	131.03499	-1.9252	$C_5H_8O_4$	3
Nucleosides and nucleotides	URIDINE 5'-DIPHOSPHO-N-ACETYLGALACTOSAMINE	208331	1.348	606.07485	[M-H]-	606.07428	0.9175	$C_{17}H_{27}N_3O_{17}P_2$	27
	URIDINE-5-MONOPHOSPHATE	119084	1.225	323.03020	[M-H]-	323.02859	4.9824	$C_9H_{13}N_2O_9P$	10
	Guanosine	107624	1.915	282.08457	[M-H]-	282.08438	0.6308	$C_{10}H_{13}N_5O_5$	4
	URIDINE-5-MONOPHOSPHATE	66649	1.63	323.02961	[M-H]-	323.02859	3.1560	$C_9H_{13}N_2O_9P$	9
	Uridine	42745	1.672	243.06246	[M-H]-	243.06226	0.8243	$C_9H_{12}N_2O_6$	11
	Uridine 5'-diphosphate	27091	1.183	402.99530	[M-H]-	402.99493	0.9394	$C_9H_{14}N_2O_{12}P_2$	6
	Guanosine 5'-monophosphate	17701	1.225	362.05153	[M-H]-	362.05072	2.2291	$C_{10}H_{14}N_5O_8P$	5
	Adenosine	16285	1.712	266.08949	[M-H]-	266.08948	0.0472	$C_{10}H_{13}N_5O_4$	2
	2'-DEOXYGUANOSINE 5'-MONOPHOSPHATE	15733	1.183	346.05652	[M-H]-	346.05582	2.0565	$C_{10}H_{14}N_5O_7P$	7
	alpha-D-Glucose-1,6-diphosphate	15409	1.183	338.98999	[M-H]-	338.98877	3.5889	$C_6H_{14}O_{12}P_2$	6
	Inosine	7711	1.956	267.07306	[M-H]-	267.07349	-1.6214	$C_{10}H_{12}N_4O_5$	2
	Guanosine-3',5'-cyclic monophosphate	6075	1.753	344.04187	[M-H]-	344.04016	4.9754	$C_{10}H_{12}N_5O_7P$	6
	N-Allyladenosine	3083	2.827	306.12082	[M-H]-	306.12079	0.1386	$C_{13}H_{17}N_5O_4$	2
	2'-Deoxyguanosine monohydrate	2376	2.158	266.09078	[M-H]-	266.08948	4.8951	$C_{10}H_{13}N_5O_4$	3

Carbohydrates	Ribitol	242738	1.083	151.06181	[M-H]-	151.06120	4.0578	$C_5H_{12}O_5$	16
	GLUCOSE 1-PHOSPHATE	93728	1.13	259.02392	[M-H]-	259.02243	5.7040	$C_6H_{13}O_9P$	4
	Mannitol	75941	1.083	181.07232	[M-H]-	181.07176	3.0833	$C_6H_{14}O_6$	17
	Trehalose-6-Phosphate	58559	1.13	421.06760	[M-H]-	421.07526	-18.2056	$C_{12}H_{23}O_{14}P$	3
	Palatinose	55367	1.083	341.10958	[M-H]-	341.10895	1.8907	$C_{12}H_{22}O_{11}$	10
	GLUCONIC ACID	50421	1.083	195.05149	[M-H]-	195.05103	2.3775	$C_6H_{12}O_7$	18
	Mannose	33085	1.042	179.05627	[M-H]-	179.05611	0.8844	$C_6H_{12}O_6$	22
	D-Sorbitol-6-phosphate	26151	1.13	260.98985	[M-H]-	261.03809	3.7060	$C_6H_{15}O_9P$	3
	2-Deoxyribose-5-phosphate	24045	1.183	213.01815	[M-H]-	213.01697	5.5713	$C_5H_{11}O_7P$	2
	N-Fructosyl pyroglutamate	23013	1.63	290.08880	[M-H]-	290.08810	2.1716	$C_{11}H_{17}NO_8$	4
	N-Acetylmannosamine	3423	2.118	220.08336	[M-H]-	220.08266	3.1773	$C_8H_{15}NO_6$	2
Peptides	Pyroglutamyl-Isoleucine	5755	7.18	241.11916	[M-H]-	241.11938	-0.9149	$C_{11}H_{18}N_2O_4$	5
	Glu-Val-Phe	5320	7.828	392.18170	[M-H]-	392.18271	-2.5731	$C_{19}H_{27}N_3O_6$	9
	Proline-hydroxyproline	5185	5.502	227.10235	[M-H]-	227.10373	-6.0789	$C_{10}H_{16}N_2O_4$	6
	Pyroglutamyl-Isoleucine	4727	6.893	241.11918	[M-H]-	241.11938	-0.8320	$C_{11}H_{18}N_2O_4$	3
	Isoleucylisoleucine	4143	6.532	243.17020	[M-H]-	243.17140	-4.9191	$C_{12}H_{24}N_2O_3$	3
	Gly-Leu	2677	3.85	187.10934	[M-H]-	187.10881	2.8010	$C_8H_{16}N_2O_3$	6
	Ala-Phe	1919	5.462	235.10974	[M-H]-	235.10881	3.9304	$C_{12}H_{16}N_2O_3$	2
Other natural products	agnuside	7451	11.381	465.14435	[M-H]-	465.14023	8.8464	$C_{22}H_{26}O_{11}$	3
Environmental contaminants / Synthetic compounds	Kresoxim-methyl acid	25501	1.083	298.11557	[M-H]-	298.10849	23.7780	$C_{17}H_{17}NO_4$	2

	Paracetamol	17751	1.956	150.05578	[M-H]-	150.05605	-1.8131	$C_8H_9NO_2$	2
	2-Hydroxycarbamazepine	8917	1.672	251.07752	[M-H]-	251.08260	-20.2370	$C_{15}H_{12}N_2O_2$	1
	Methoxyfenozide	8564	11.381	367.21175	[M-H]-	367.20273	24.6014	$C_{22}H_{28}N_2O_3$	1
	Coumatetralyl	7241	5.936	291.09905	[M-H]-	291.10266	-12.4283	$C_{19}H_{16}O_3$	4
	Di-n-butyl phthalate	4746	16.435	277.14475	[M-H]-	277.14453	0.7840	$C_{16}H_{22}O_4$	8
	Cyclamate	4698	1.956	178.05044	[M-H]-	178.05434	-21.8921	$C_6H_{13}NO_3S$	1
Putative / Unknown compounds	NP-000062(6)	47749	7.26	563.13956	[M-H]-	563.14063	-1.8985	$C_{26}H_{28}O_{14}$	27
	(2S,3R,4R,5S,6S)-3,5-bis(acetyloxy)-2-{[(1S,2S,4S,5S,6R,10S)-2-(hydroxymethyl)-10-{[(2S,3R,4S,5S,6R)-3,4,5-trihydroxy-6-(hydroxymethyl)oxan-2-yl]oxy}-3,9-dioxatricyclo[4.4.0.0?,?]dec-7-en-5-yl]oxy}-6-methyloxan-4-yl (2E)-3-phenylprop-2-enoate	23922	1.083	359.11956	[M-H]-	359.11362	16.5226	$C_{19}H_{20}O_7$	2
	NCGC00380353-01_C15H18O6_Pentaleno[1,6a-c]pyran-9-carboxylic acid, 1,3,4,7,7a,9a-hexahydro-4-hydroxy-4-(hydroxymethyl)-6,7-dimethyl-3-oxo-, (4R,7S,7aR,9aR)-	16457	10.333	293.10382	[M-H]-	293.10306	2.5867	$C_{15}H_{18}O_6$	9
	Hippurate	14012	2.279	178.05046	[M-H]-	178.05096	-2.8457	$C_9H_9NO_3$	3
	GLUCOSAMINATE	13497	1.042	194.06721	[M-H]-	194.06702	1.0271	$C_6H_{13}NO_6$	7
	1,7-bis(4-hydroxyphenyl)heptane-3,5-diol	13403	9.648	315.15986	[M-H]-	315.16019	-1.0242	$C_{19}H_{24}O_4$	11
	(2Z)-4,6-dihydroxy-2-[(4-hydroxy-3,5-dimethoxyphenyl)methylidene]-1-benzofuran-3-one	12440	10.983	329.06545	[M-H]-	329.06668	-3.7267	$C_{17}H_{14}O_7$	6
	2-nitrophenol	7882	9.562	138.01985	[M-H]-	138.01967	1.3291	$C_6H_5NO_3$	2
	C20H22O8	7848	8.155	389.12550	[M-H]-	389.12420	3.2864	$C_{20}H_{22}O_8$	5

6-Hydroxynicotinic acid	7712	2.319	138.01970	[M-H]-	138.01967	0.2423	$C_6H_5NO_3$	2
(4E,8E)-10-(4-hydroxy-6-methoxy-7-methyl-3-oxo-1H-2-benzofuran-5-yl)-4,8-dimethyldeca-4,8-dienoic acid	6548	5.285	387.18268	[M-H]-	387.18130	3.5328	$C_{22}H_{28}O_6$	2
C12-AE1S (TENTATIVE)	6092	12.976	309.16934	[M-H]-	309.17413	-15.4566	$C_{14}H_{30}O_5S$	2
NCGC00381155-01_C20H18O8_2-Furanacetic acid, 3-(3,4'-dihydroxy-2,5-dimethoxy[1,1'-biphenyl]-4-yl)-2,5-dihydro-5-oxo-	5853	9.396	385.09178	[M-H]-	385.09290	-2.8852	$C_{20}H_{18}O_8$	8
NCGC00380357-01!7-[3,5-dihydroxy-4-(4-hydroxyphenyl)-2-methoxyphenyl]-3-methoxy-3,4-dihydrooxepine-2,5-dione	5837	8.608	385.09270	[M-H]-	385.09290	-0.4962	$C_{20}H_{18}O_8$	6
[(4E)-7-acetyloxy-6-hydroxy-2-methyl-10-oxo-2,3,6,7,8,9-hexahydrooxecin-3-yl] (E)-but-2-enoate	5209	9.859	325.12968	[M-H]-	325.12927	1.2408	$C_{16}H_{22}O_7$	5
4-[2-(6,7-dihydroxy-1,2,4a-trimethylspiro[3,4,6,7,8,8a-hexahydro-2H-naphthalene-5,2'-oxirane]-1-yl)ethyl]-2-hydroxy-2H-furan-5-one	3592	11.421	365.19655	[M-H]-	365.19696	-1.1288	$C_{20}H_{30}O_6$	5
8phiC8SPC	3538	18.884	299.09576	[M-H]-	299.09586	-0.3626	$C_{14}H_{20}O_5S$	1
10-Hydroxydecanoic acid	3535	10.105	187.13357	[M-H]-	187.13397	-2.1270	$C_{10}H_{20}O_3$	3
CMPF	3470	10.02	239.09182	[M-H]-	239.09250	-2.8322	$C_{12}H_{16}O_5$	5
Folinic acid	2849	5.643	472.15298	[M-H]-	472.15863	-11.9444	$C_{20}H_{23}N_7O_7$	4
Xanthurenic acid	2578	4.021	204.03018	[M-H]-	204.03023	-0.2511	$C_{10}H_7NO_4$	8
4,6-dihydroxy-3-(3-hydroxy-2,5-dimethylphenoxy)-5-methoxy-2,5-dimethylcyclohex-2-en-1-one	1927	9.939	321.13795	[M-H]-	321.13437	11.1730	$C_{17}H_{22}O_6$	3
NCGC00385574-01![5-acetyloxy-1,7-bis(3,4-dihydroxyphenyl)heptan-3-yl] acetate	1906	13.016	431.17176	[M-H]-	431.17114	1.4347	$C_{23}H_{28}O_8$	2
Apigenin	1871	10.903	269.04504	[M-H]-	269.04553	-1.8843	$C_{15}H_{10}O_5$	3
9-(2,3-dihydroxypropoxy)-9-oxononanoic acid	1690	8.482	261.13534	[M-H]-	261.13437	3.7454	$C_{12}H_{22}O_6$	6

Divaricatinic acid	1324	7.954	209.08117	[M-H]-	209.08194	-3.6467	$C_{11}H_{14}O_4$	4
4-Hydroxybenzotriazole	817	8.281	134.03744	[M-H]-	134.03598	10.8529	$C_6H_5N_3O$	1

Note. Compound names are reported as putative LC–MS/MS annotations. All listed analytical features were acquired with MS/MS data; however, feature-level product-ion spectra, individual similarity scores, and matched-fragment counts were not traceable for every entry in the archived summary output. Therefore, the annotations should not be interpreted as confirmed compound identities. Authentic reference standards were not analyzed. The adduct setting was $[M-H]^-$ for negative-ion mode. The four features at RT 12.775, 15.873, 17.356, and 18.255 min are conservatively reported as "Dodecanoic acid/Lauric acid candidate" because dodecanoic acid and lauric acid are synonymous; the distinct chromatographic features were retained without assigning them as confirmed isomers, alternative adducts, or chromatographic artifacts.

Table S3. Exploratory protein database best matches detected in the aqueous truffle extract by LC-MS/MS.

Best database hit	Protein annotation	Functional category
GL82_ORYSJ	Germin-like protein 8-2	Stress and defense proteins
GLUB1_ORYSJ	Glutelin type-B 1	Storage proteins
GLUB4_ORYSJ	Glutelin type-B 4	Storage proteins
GLUA2_ORYSJ	Glutelin type-A 2	Storage proteins
GLUA3_ORYSJ	Glutelin type-A 3	Storage proteins
CUCIN_ORYSI	Cupincin	Storage proteins
HSP71_PICAN	Heat shock protein 70 1	Molecular chaperones
HSP76_HUMAN	Heat shock 70 kDa protein 6	Molecular chaperones
HSP70_LEIAM	Heat shock 70 kDa protein	Molecular chaperones
HSP68_DROME	Heat shock protein 68	Molecular chaperones
HSP71_CANAL	Heat shock protein SSA1	Molecular chaperones
BIP4_ORYSJ	Heat shock 70 kDa protein BIP4	Molecular chaperones
DNAK_DESRM	Chaperone protein DnaK	Molecular chaperones
DNAK_CLOAB	Chaperone protein DnaK	Molecular chaperones
G3P_SCHCO	Glyceraldehyde-3-phosphate dehydrogenase	Glycolysis
G3PC3_ORYSJ	Glyceraldehyde-3-phosphate dehydrogenase 3, cytosolic	Glycolysis
EF1A_SERIN	Elongation factor 1-alpha	Translation
EF1A_SCHCO	Elongation factor 1-alpha	Translation
TBB_SCHCO	Tubulin beta chain	Cytoskeleton
HSP81_ORYSI	Heat shock protein 81-1	Molecular chaperones
HSC82_YEAST	ATP-dependent molecular chaperone HSC82	Molecular chaperones
ACT1_SCHCO	Actin-1	Cytoskeleton
GLB63_ORYSJ	63 kDa globulin-like protein	Storage proteins
PRO7_ORYSI	Prolamin PPROL 14E	Storage proteins
IAAS_ORYSJ	Alpha-amylase/subtilisin inhibitor	Defense proteins
RS5B_DROME	40S ribosomal protein S5b	Ribosome
BT1_MAIZE	Adenine nucleotide transporter BT1, chloroplastic/amyloplastic/mitochondrial	ATP transport
AI171_ORYSJ	17kDa alpha-amylase/trypsin inhibitor 1	Defense proteins
IF4A1_ORYSJ	Eukaryotic initiation factor 4A-1	Translation
ALDH4_YEAST	Potassium-activated aldehyde dehydrogenase, mitochondrial	Redox regulation
ALDH_ASPNG	Aldehyde dehydrogenase	Redox regulation
RS9A_SCHPO	40S ribosomal protein S9-A	Ribosome
ATPBM_ARATH	ATP synthase subunit beta-1, mitochondrial	ATP synthesis
ATPB1_PELCD	ATP synthase subunit beta 1	ATP synthesis
ADT_NEUCR	ADP,ATP carrier protein	ATP transport
6PGD1_YEAST	6-phosphogluconate dehydrogenase, decarboxylating 1	Pentose phosphate pathway
RS9_DROME	40S ribosomal protein S9	Ribosome
GL81_ORYSJ	Putative germin-like protein 8-1	Stress and defense proteins
SCP_CHIOP	Sarcoplasmic calcium-binding protein (Fragment)	Calcium-binding proteins

RL2_METB6	50S ribosomal protein L2	Ribosome
G6PD_TAKRU	Glucose-6-phosphate 1-dehydrogenase	Pentose phosphate pathway
SAHH_SCHPO	Adenosylhomocysteinase	Methionine metabolism
MDHM_BRANA	Malate dehydrogenase, mitochondrial	TCA cycle
BROM1_ANACO	Fruit bromelain	Protein degradation
ATPF_YEAST	ATP synthase subunit 4, mitochondrial	ATP synthesis
MDHM_CAEEL	Probable malate dehydrogenase, mitochondrial	TCA cycle
PGDH_MOUSE	15-hydroxyprostaglandin dehydrogenase [NAD(+)]	Redox regulation
ACT17_DICDI	Actin-17	Cytoskeleton
NLTP1_ORYSI	Non-specific lipid-transfer protein 1	Lipid metabolism
FTSI4_ARATH	Probable inactive ATP-dependent zinc metalloprotease FTSHI 4, chloroplastic	Protein degradation
GUAA_HELAH	GMP synthase [glutamine-hydrolyzing]	Nucleotide metabolism
ENO_ALTAL	Enolase	Glycolysis
PRDX_ASCSU	Peroxiredoxin	Antioxidant defense
NDK_BACHD	Nucleoside diphosphate kinase	Nucleotide metabolism
RL5_CUCSA	60S ribosomal protein L5	Ribosome
DHSO_ARATH	Sorbitol dehydrogenase	Carbohydrate metabolism
RL4A_SCHPO	60S ribosomal protein L4-A	Ribosome
Y3511_METJA	Uncharacterized protein MJECL11	Uncharacterized proteins
DNAK_GRATL	Chaperone protein dnaK	Molecular chaperones
Y2672_THEFY	UPF0234 protein Tfu_2672	Uncharacterized proteins
EF2_BOVIN	Elongation factor 2	Translation
RL13_CANAL	60S ribosomal protein L13	Ribosome
GLGL1_MAIZE	Glucose-1-phosphate adenylyltransferase large subunit 1, chloroplastic/amyloplastic	Carbohydrate metabolism
NLTP2_MAIZE	Probable non-specific lipid-transfer protein 2	Lipid metabolism
PPDK2_ORYSJ	Pyruvate, phosphate dikinase 2	Carbohydrate metabolism
SBNO2_MOUSE	Protein strawberry notch homolog 2	RNA processing and regulation
PGK_MYCBO	Phosphoglycerate kinase	Glycolysis
ARTA_ASPFN	14-3-3 family protein artA	Signal transduction and regulation
KTHY_CHLPM	Thymidylate kinase	Nucleotide metabolism
AMPA_GEOTN	Probable cytosol aminopeptidase	Amino acid metabolism
PSAM_PROMP	Photosystem I reaction center subunit XII	Photosynthesis
TBAB_SCHCO	Tubulin alpha-1B chain	Cytoskeleton
IPYR_ASHGO	Inorganic pyrophosphatase	Phosphate metabolism
RL15_FRAP2	50S ribosomal protein L15	Ribosome
FUSIL_DROME	RNA-binding protein fusilli	RNA processing and regulation
ARGJ_ACIAD	Arginine biosynthesis bifunctional protein ArgJ	Amino acid metabolism
SAM11_HUMAN	Sterile alpha motif domain-containing protein 11	Signal transduction and regulation
YIGA_SALTY	Uncharacterized protein YigA	Uncharacterized proteins
GYRA_CLOAB	DNA gyrase subunit A	Chromatin and DNA regulation
HEAT1_HUMAN	HEAT repeat-containing protein 1	RNA processing and regulation

Y1823_CLOD6	UPF0246 protein CD630_18230	Uncharacterized proteins
EIF3F_ASPCL	Eukaryotic translation initiation factor 3 subunit F	Translation
RA16_ORYSJ	Alpha-amylase/trypsin inhibitor RA16	Defense proteins
PP1_EMENI	Serine/threonine-protein phosphatase PP1	Signal transduction and regulation

Note. Entries were retained only when at least one matched peptide had an Ions Score > 43 (p < 0.05 according to the Mascot search report). Protein annotations were taken from the Mascot report. Functional categories were retained from the original Supplementary Table S3 where available; newly retained entries were assigned to the closest existing descriptive category based on their reported protein annotation. These categories are provided for descriptive organization only and do not constitute functional validation.

Table S4. MTT-based metabolic activity and cellular reducing capacity in non-malignant cells treated with truffle extract.

Cell line	mock	1 μg/ml	0.5 μg/ml	0.25 μg/ml	0.1 μg/ml	0.05 μg/ml	0.01 μg/ml	Source
Vero	100	123.69±2.5*	129.21±5.2*	141.42±5.9*	143.22±3.9*	126.25±4.2*	134.22±3.9*	African green monkey kidney epithelial
MDBK	100	107.14±6.5	139.22±3.1*	163.39±6.82**	143.79±4.1*	136.40±2.9*	115.25±4.9*	Madin-Darby bovine kidney
Nthy-ori3-1	100	102.25±4.5	117.84±1.7*	122.37±0.498*	134.07±0.4*	117.93±3.4*	113.0±0.9*	Human thyroid follicular epithelial
HFL-1	100	117.56±2.2*	126.55±2.3*	144.90±3.13*	110.71±3.1*	113.51±3.3*	112.10±3.9*	Human fetal lung fibroblast
BEAS 2B	100	117.93±6.1*	117.2±1.4*	135.54±2.9*	119.61±4.2*	119.10±6.6*	118.88±6.2*	Normal Bronchial Epithelial
HEK-293	100	118.86±6.6*	120.32±2.5*	118.94±4.2*	130.25±3.4*	121.26±1.7*	104.43±4.3	Human embryonic kidney cells

Note. Data are presented as the mean ± SD from three independent experiments. Statistical significance was determined using one-way ANOVA followed by Duncan's multiple range test (DMRT) in GraphPad Prism 9 software. *p < 0.05 and **p < 0.01 versus the untreated control.

Table S5. MTT-based metabolic activity and cellular reducing capacity in cancer cells treated with truffle extract.

Cell line	mock	1 μg/ml	0.5 μg/ml	0.25 μg/ml	0.1 μg/ml	Source
B16-F10	100	98.21±0.998	97.46±0.6	97.19±1.2	97.41±1.3	Mouse murine melanoma
N2a	100	98.68±3.21	97.14±1.1	96.02±1.1	97.7±1.5	Mouse neuroblastoma N2a cells
OECM-1	100	98.94±0.025	97.9±3.1	97.71±1.7	99.36±3.2	Human oral squamous carcinoma
BCPAP	100	75.51±0.788**	86.01±1.3*	89.55±2.3*	91.62±2.8	Human papillary thyroid carcinoma
HeLa	100	98.64±0.355	97.13±0.4	96.25±3.6	98.14±1.6	Human cervical adenocarcinoma
AGS	100	96.01±0.720	99.23±0.8	100.25±1.3	100.59±1.1	Human gastric adenocarcinoma
A549	100	99.59±0.493	99.86±1.5	98.75±1.4	100.65±0.6	Human lung carcinoma epithelial cells
HepG2	100	93.72±0.149	97.79±2.6	100.10±0.8	99.88±1.0	Human hepatoblastoma
HSC-3	100	96.38±1.205	99.49±1.0	97.13±0.7	98.05±1.7	Human Tongue Squamous Carcinoma
MCF-7	100	97.49±1.606	98.08±1.8	97.92±2.0	97.09±1.9	Human breast cancer cells
SW620	100	99.46±3.217	96.19±2.4	98.01±1.1	98.89±2.7	Human colorectal adenocarcinoma cells
PC-3	100	92.26±0.685	98.55±0.3	99.66±0.4	98.34±1.0	Human prostate cancer

Note. Data are presented as the mean ± SD from three independent experiments. Statistical significance was determined using one-way ANOVA followed by Duncan's multiple range test (DMRT) in GraphPad Prism 9 software. *p < 0.05 and **p < 0.01 versus the untreated control.

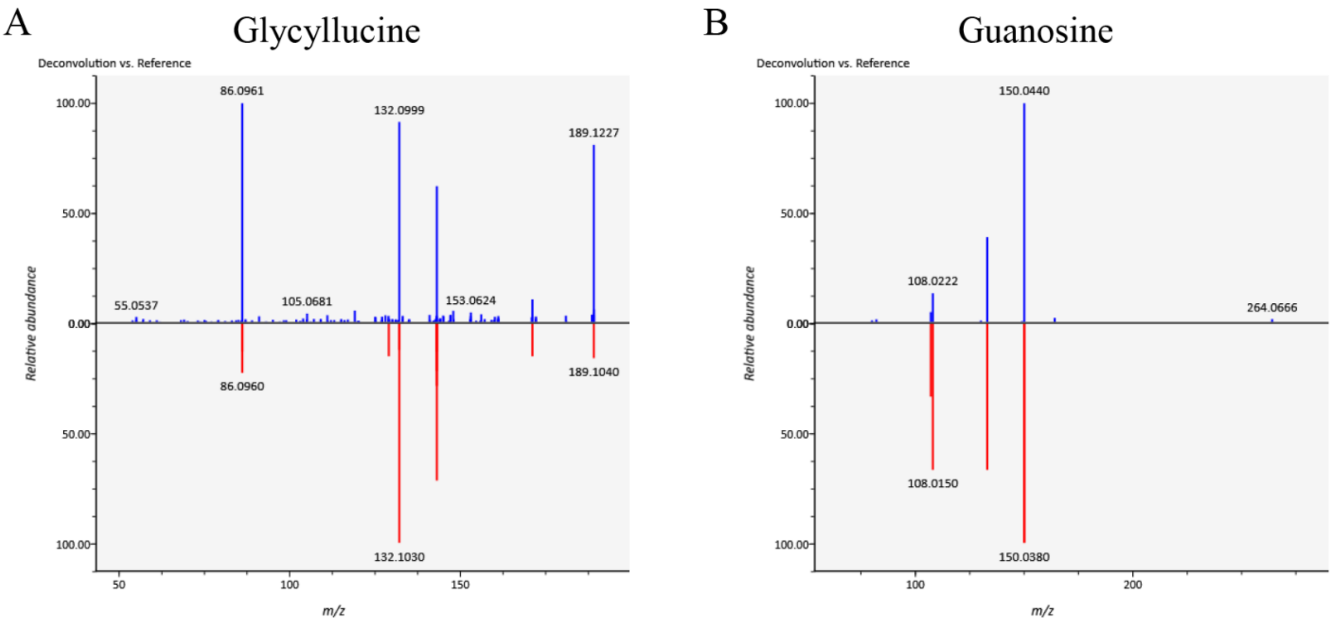

Figure S1. Representative experimental MS/MS spectra of selected putative annotations in the aqueous truffle extract. Experimental MS/MS spectra were acquired for all retained features during information-dependent acquisition. Two representative spectra are shown here as examples of the fragmentation data available for the retained annotations. The spectra correspond to selected features reported in Supplementary Tables S1 and S2. Compound names represent putative candidate annotations based on the available LC-MS/MS evidence and should not be interpreted as confirmed identifications because authentic reference standards were not analyzed.

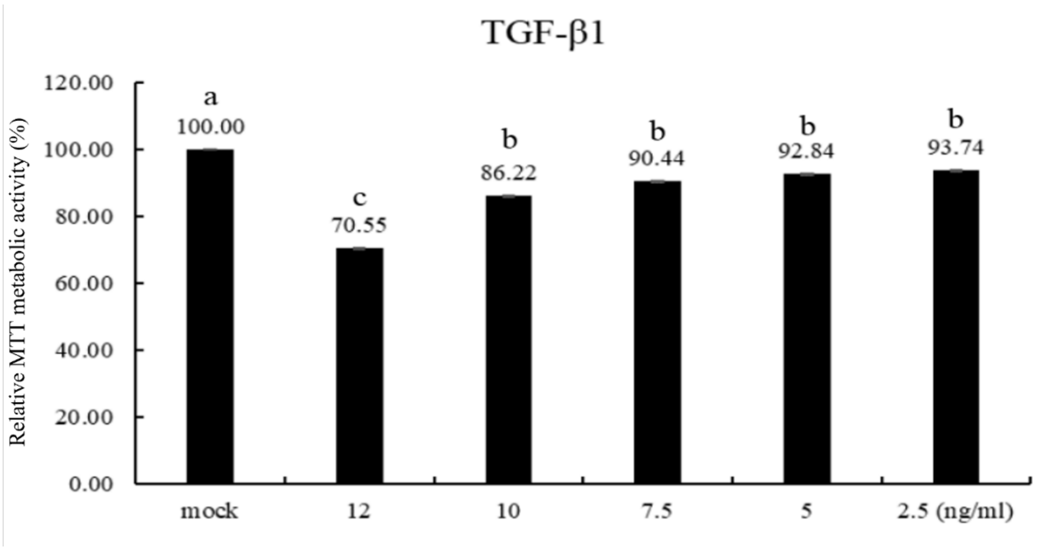

Figure S2. Effect of TGF-β1 on MTT-based cellular metabolic activity and reducing capacity in HFL-1 cells. HFL-1 cells were treated with the indicated concentrations of TGF-β1 for 24 h, and MTT-based cellular metabolic activity and reducing capacity were measured. Results were expressed relative to the untreated control, which was defined as 100%. Data are presented as the mean ± SD from three independent experiments ($n = 3$). Differences among groups were analyzed by one-way ANOVA followed by Duncan's multiple range test (DMRT) using GraphPad Prism 9 software. Different letters indicate significant differences among groups ($p < 0.05$).

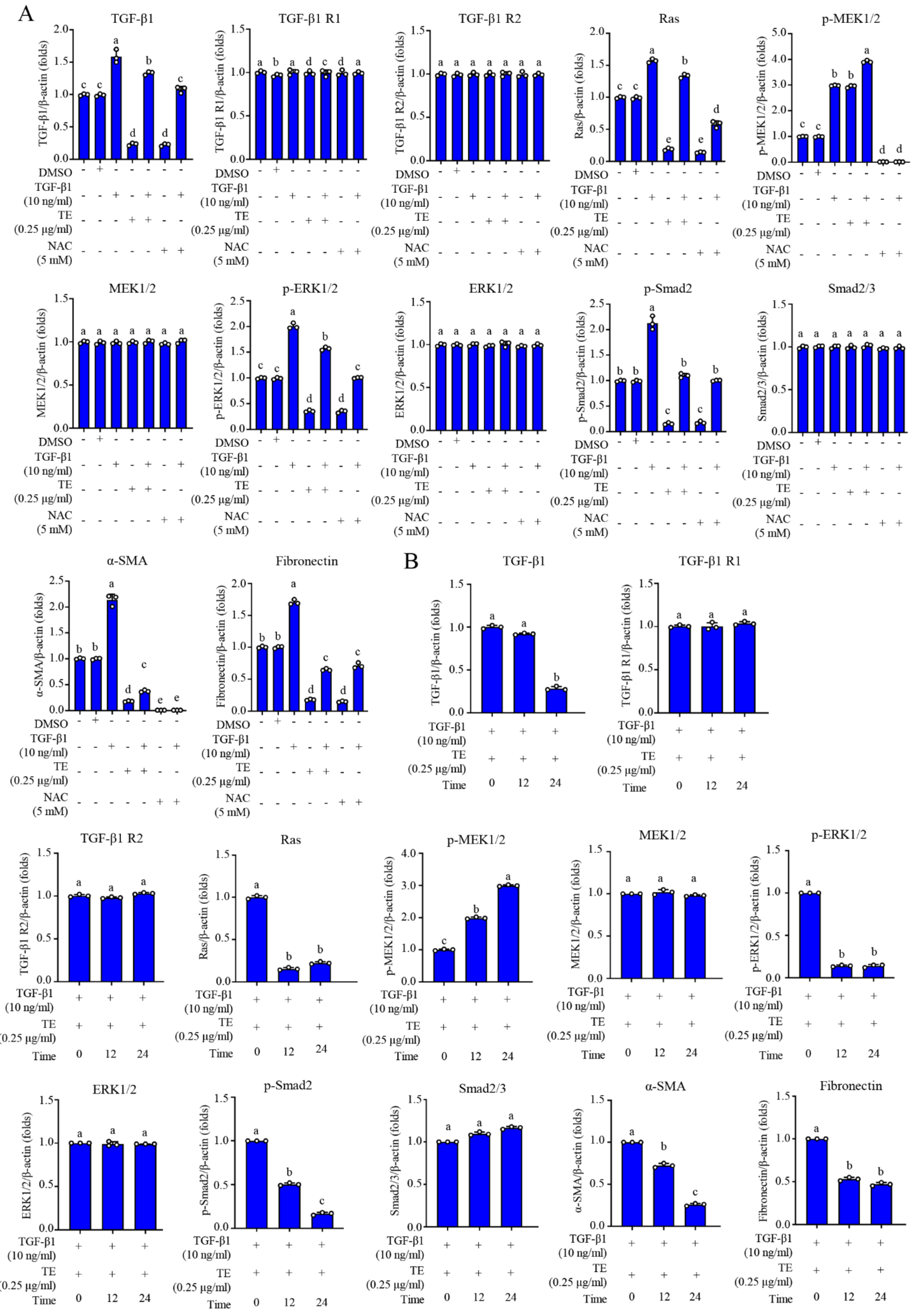

Figure S3. Quantitative analysis of TE-associated changes in TGF-β1-induced signaling and fibrotic marker proteins. Immunoblots from Figure 3A-B were quantified using ImageJ and expressed relative to the mock control (1-fold). Data are the mean ± SD from three independent experiments (n = 3). Statistical differences were analyzed by one-way ANOVA followed by DMRT; different letters indicate $p < 0.05$.

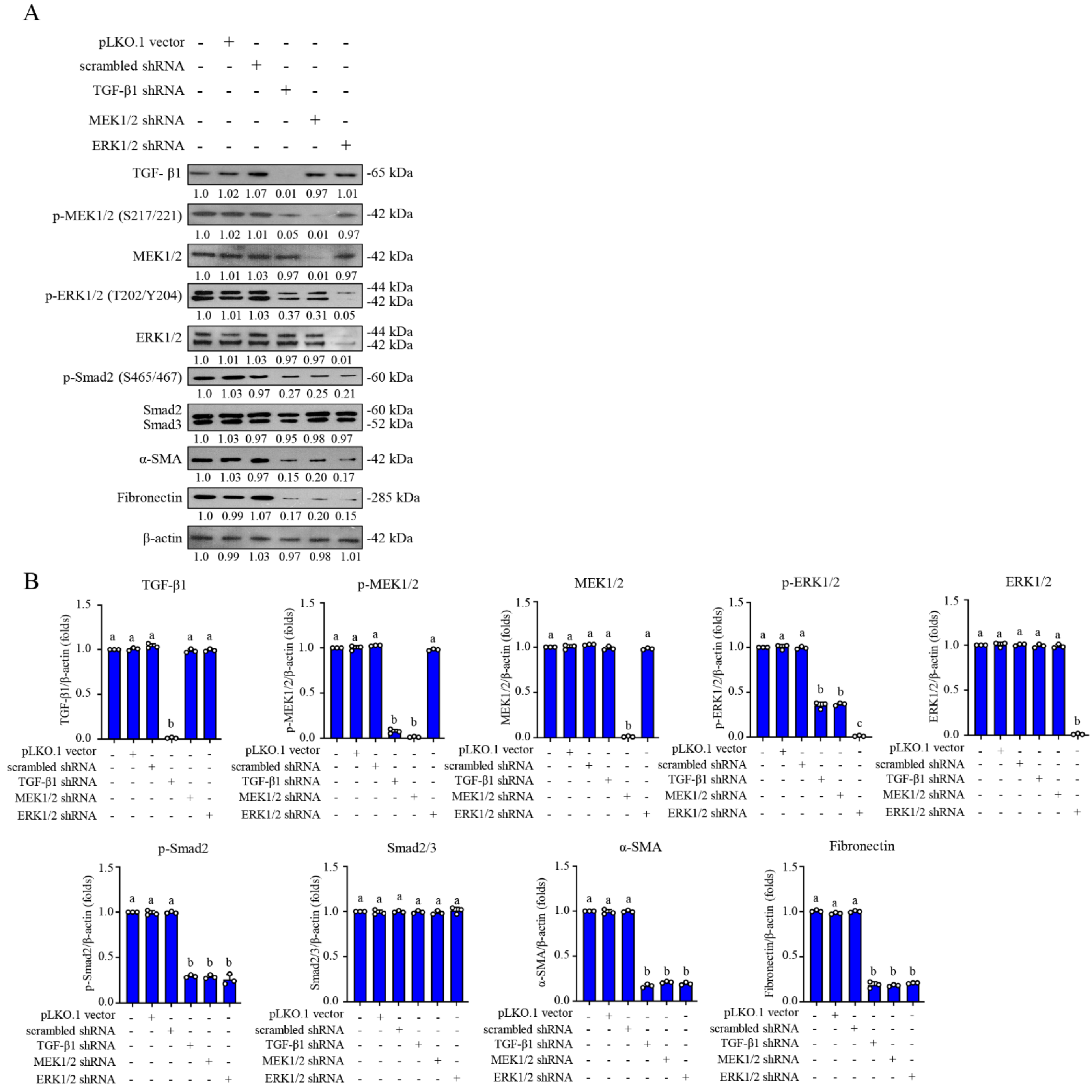

Figure S4. Effects of shRNA-mediated target reduction on fibrosis-associated proteins in HFL-1 cells. HFL-1 cells were transfected with pLKO.1, scrambled shRNA (shNC), or shRNAs targeting TGF-β1, MEK1/2, or ERK1/2; neither TE nor exogenous TGF-β1 was added. (A) Western blot analysis was performed to confirm reduction of the corresponding target proteins and to examine fibrosis-associated proteins under the shRNA-only conditions, with β-actin as the loading control. Representative blots from three independent experiments are shown. (B) Densitometric quantification of the indicated proteins. Data are presented as the mean ± SD ($n = 3$). Statistical differences were analyzed by one-way ANOVA followed by DMRT; different letters indicate $p < 0.05$.

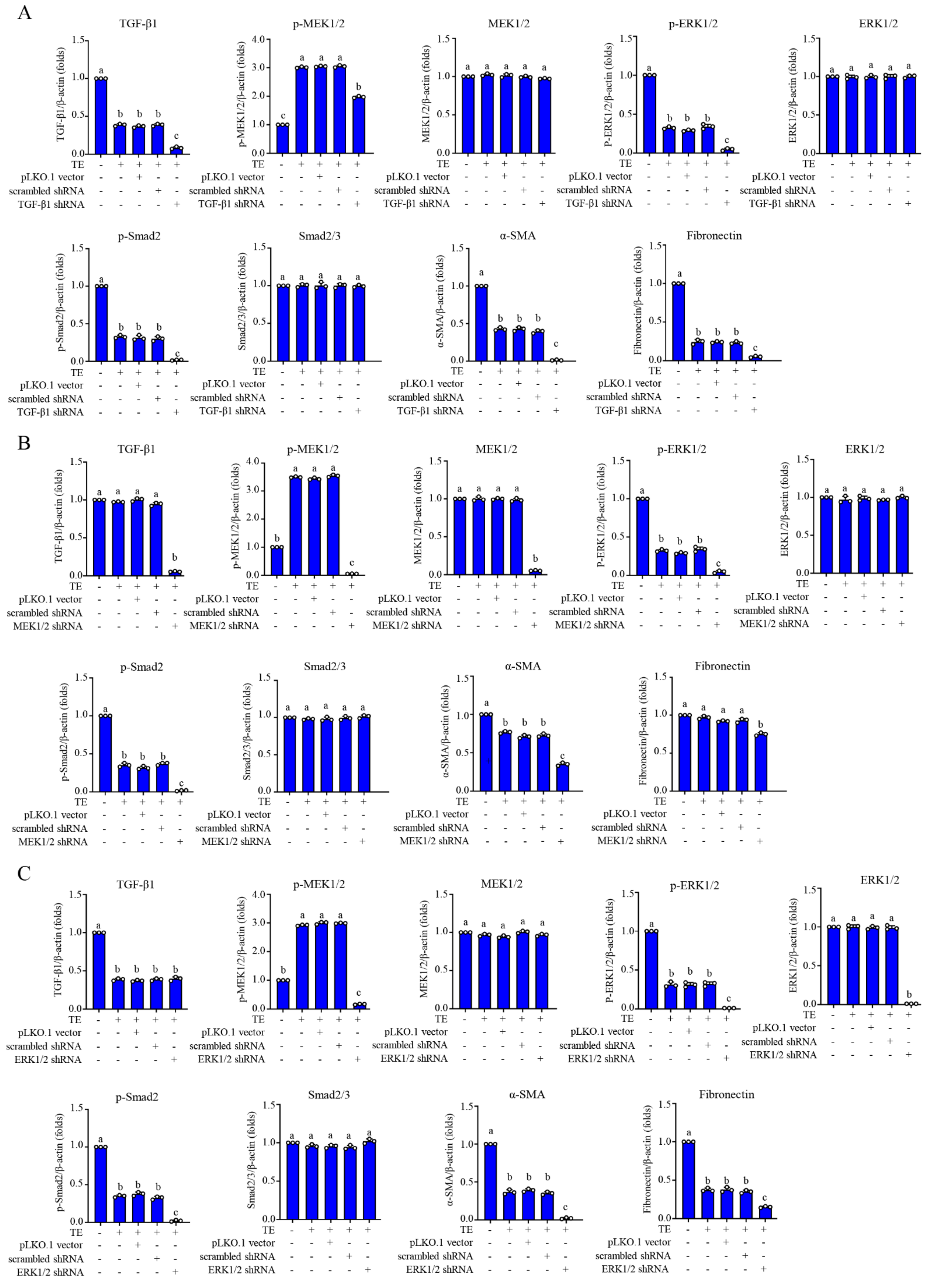

Figure S5. Quantitative analysis of the shRNA experiments shown in Figure 5A-C. HFL-1 cells were

transfected with the indicated constructs; 6 h after transfection, TE (0.25 μg/mL) was added for an additional 24 h. Exogenous TGF-β1 was not added. Immunoblots were quantified using ImageJ and expressed relative to the mock control (1-fold). Data are the mean ± SD from three independent experiments (n = 3). Statistical differences were analyzed by one-way ANOVA followed by DMRT; different letters indicate $p < 0.05$.

Figure 2A

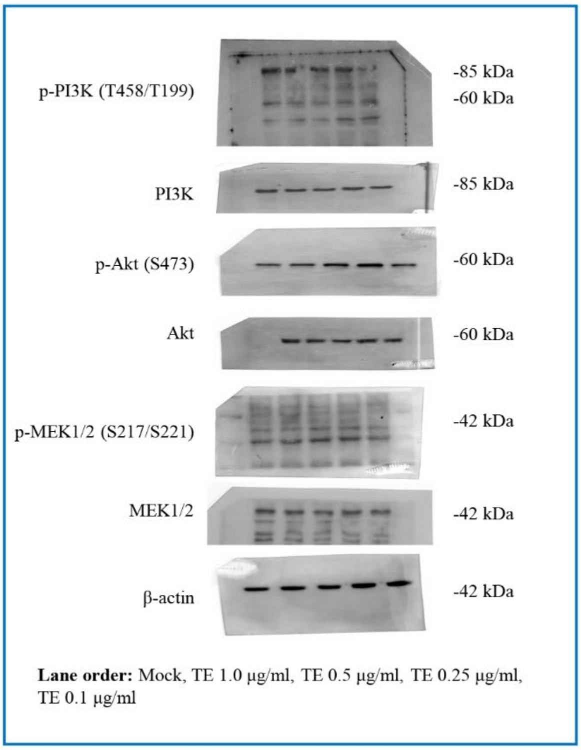

Figure 2C

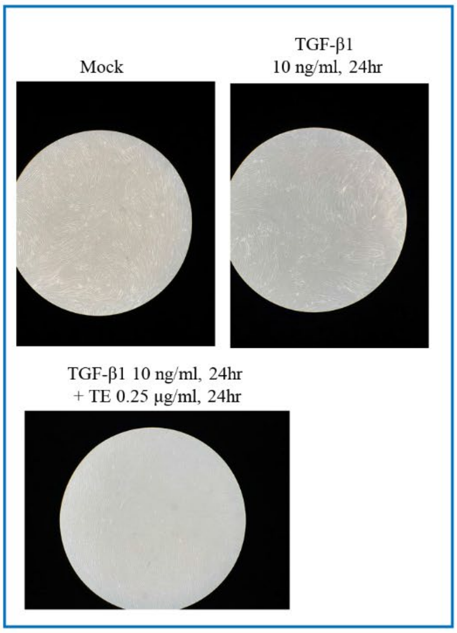

Figure 2D

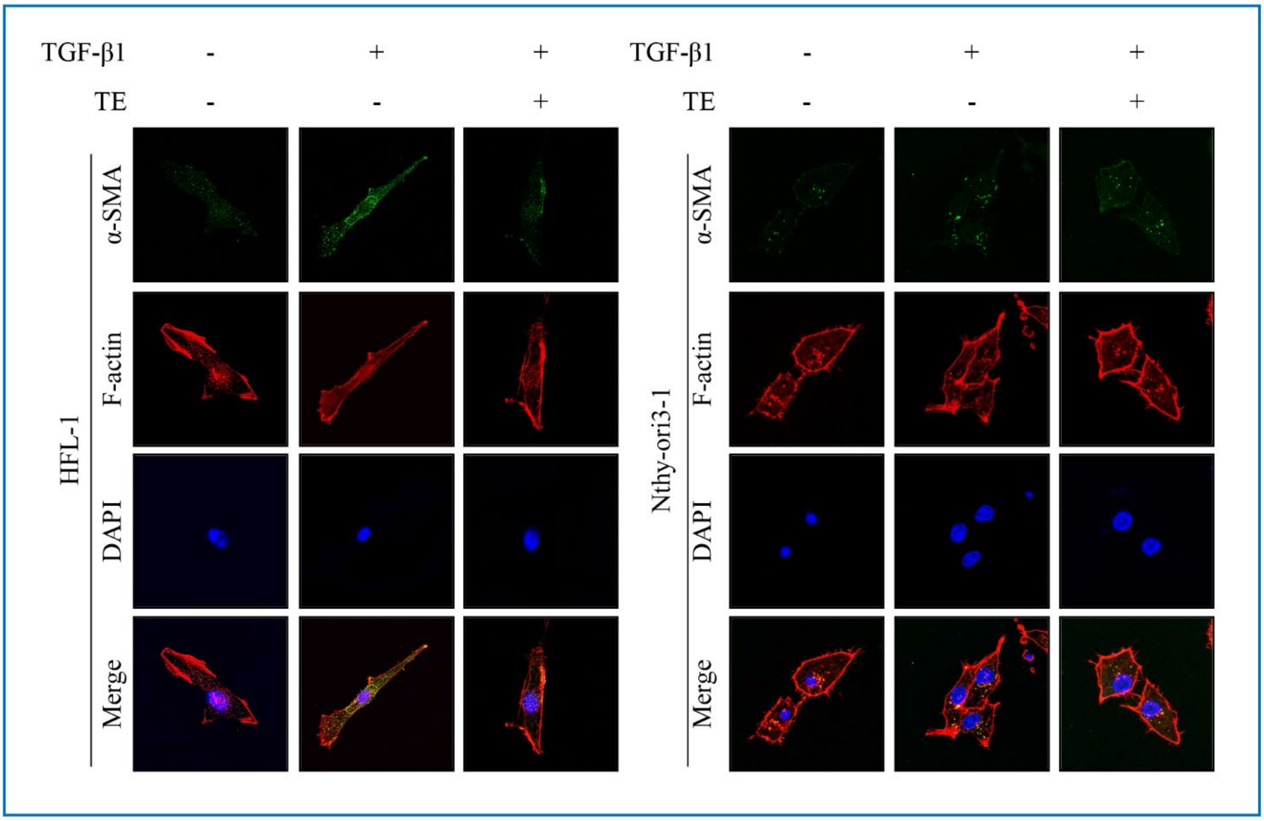

Figure 3A

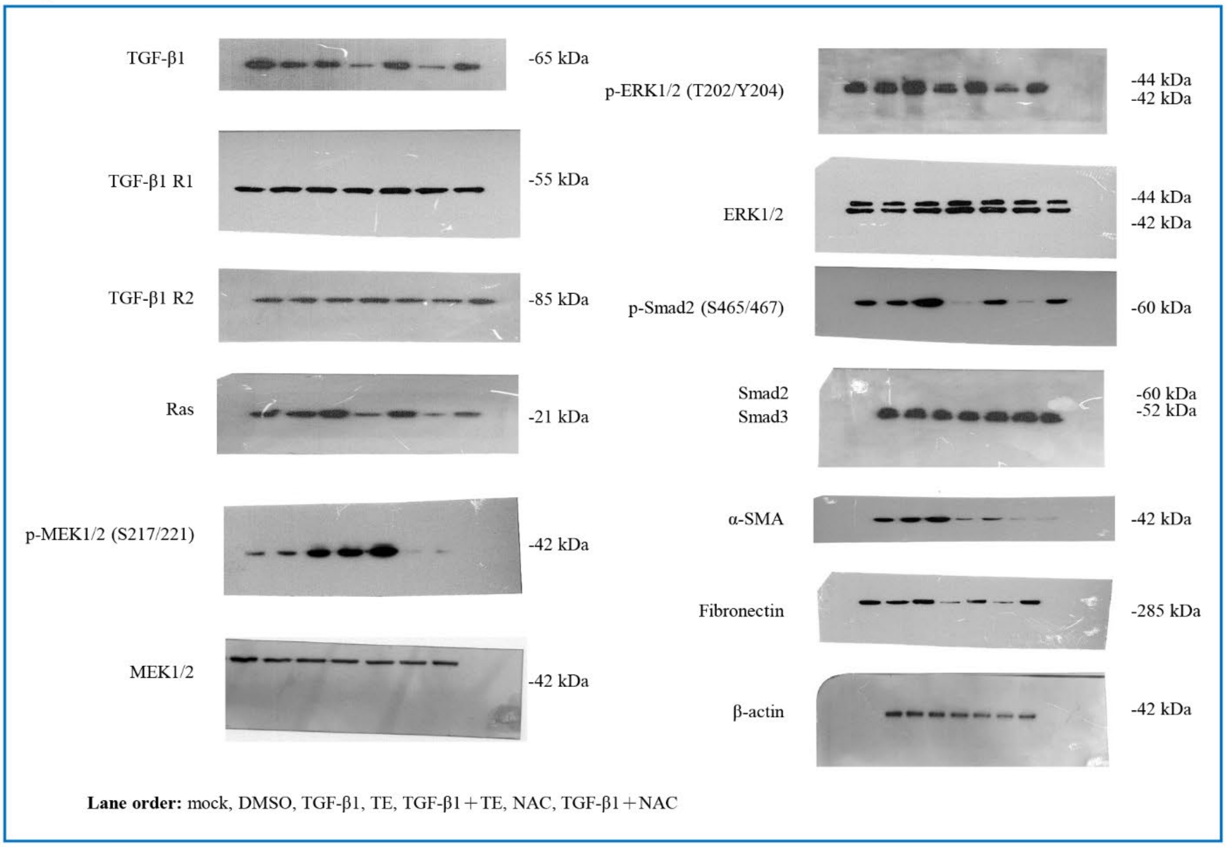

Figure 3B

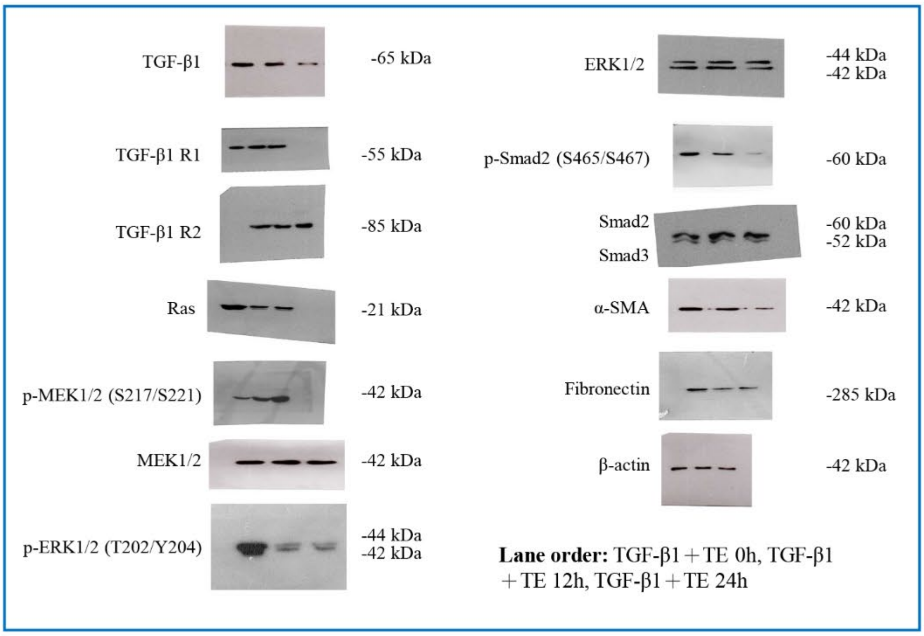

Figure 4A

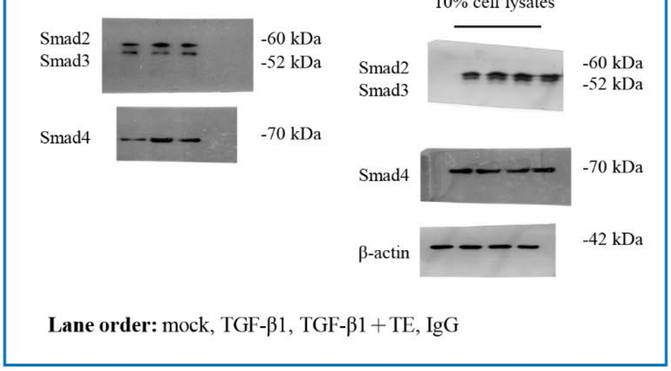

Figure 4B

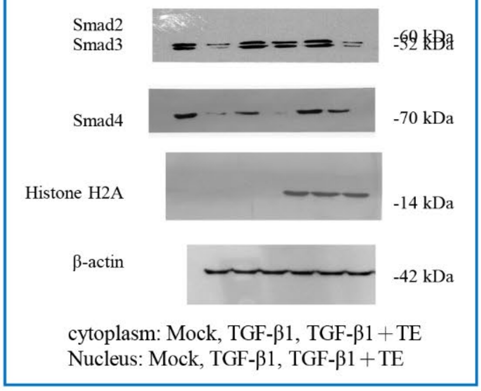

Figure S3

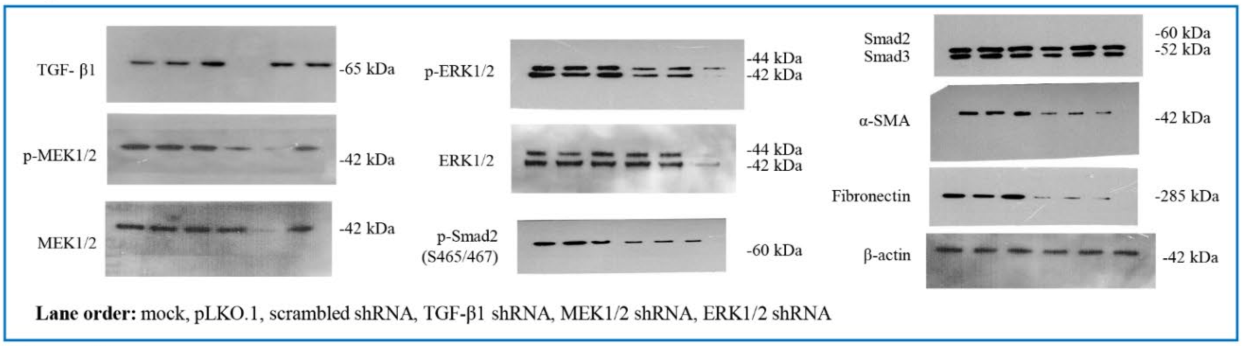

Figure 5A

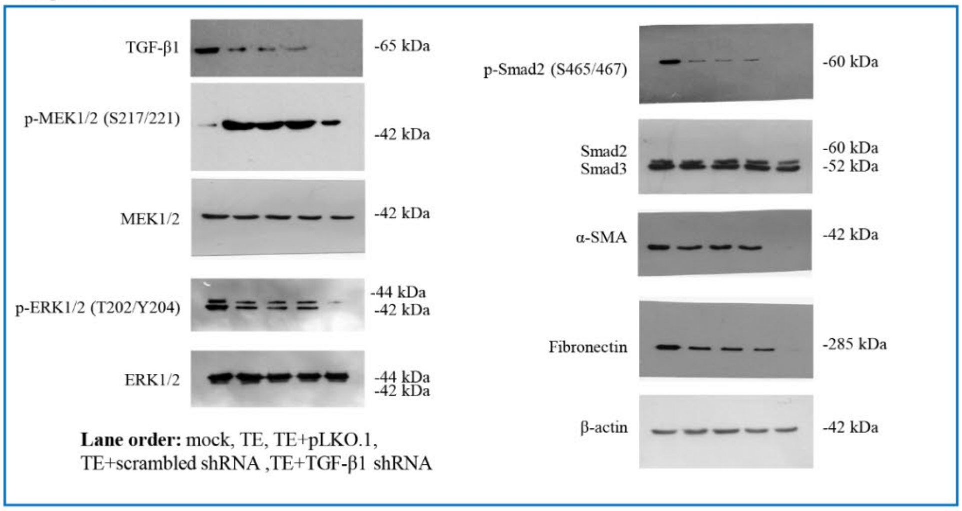

Figure 5B

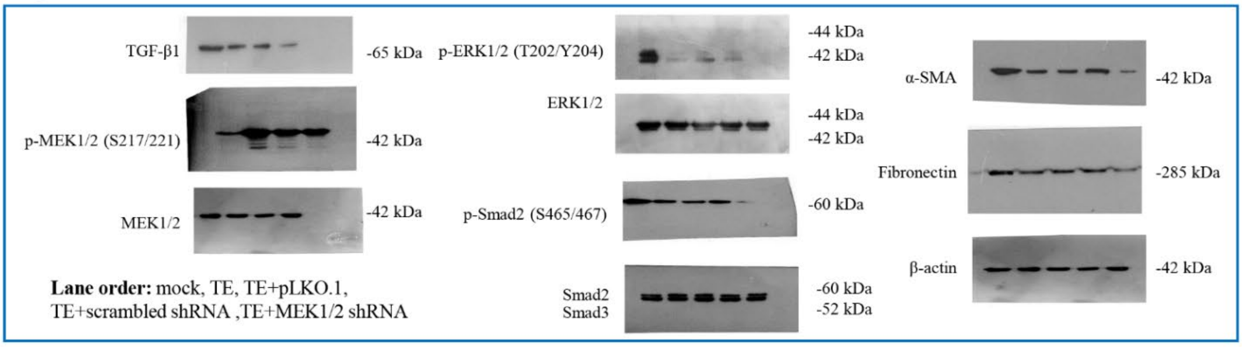

Figure 5C

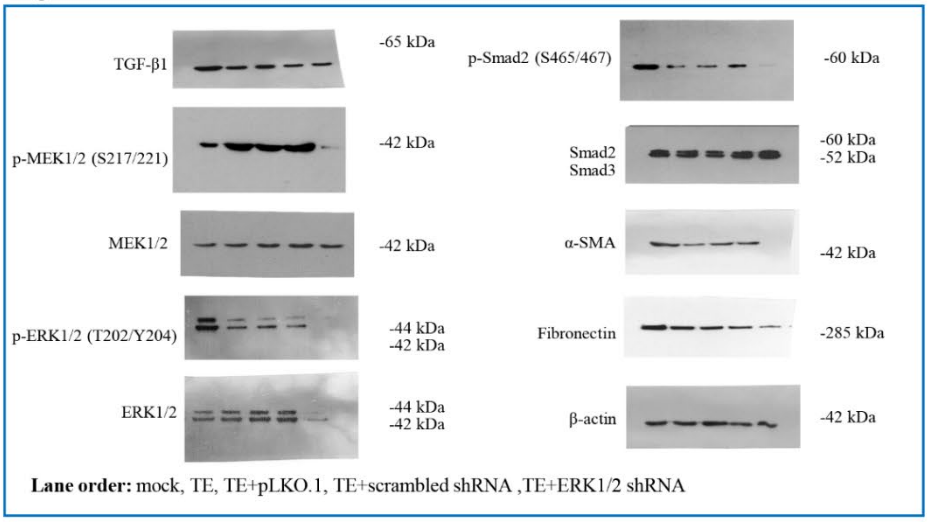

Figure S6. Original uncropped blots and source images used in this study.

Datasheet 1. Peptide-level Mascot search results, including score-based metrics, unique-peptide information, and single-entry/shared peptide assignments.

Family ID (Group)	Accession	Description	Observed	Mr (expt)	Mr (calculated)	Delta	Score	E-value	Match sequence	Unique
1	GL82_ORYSJ	Germin-like protein 8-2	412.7039	823.3933	823.3898	0.0035	47	0.022	R.VNGFVCK.N+ Carbamidomethyl (C); Deamidated (NQ)	Yes
			530.8056	1059.5967	1059.5964	0.0003	61	2.9e-05	K.IIDLLQAQF.-	Yes
			531.2932	1060.5719	1060.5804	-0.0086	51	4e-05	K.IIDLLQAQF.-+ Deamidated (NQ)	Yes
			531.2932	1060.5719	1060.5804	-0.0086	46	0.001	K.IIDLLQAQF.-+ Deamidated (NQ)	Yes
			594.8526	1187.6906	1187.6914	-0.0008	64	4.2e-06	K.KIIDLLQAQF.-	Yes
			446.2403	1780.9322	1780.9373	-0.0051	73	3.9e-07	R.LDFAPLGLNPPHTHPR.A	Yes
			594.6526	1780.9359	1780.9373	-0.0014	72	1.1e-06	R.LDFAPLGLNPPHTHPR.A	Yes
			891.4759	1780.9372	1780.9373	-0.0001	82	1.3e-07	R.LDFAPLGLNPPHTHPR.A	Yes
			594.9673	1781.8800	1781.9213	-0.0413	23	0.0065	R.LDFAPLGLNPPHTHPR.A+ Deamidated (NQ)	Yes
			599.9890	1796.9452	1796.9322	0.0130	50	9.6e-05	R.LDFAPLGLNPPHTHPR.A+ Oxidation (HW)	Yes
2	GLUB1_ORYSJ	Glutelin type-B 1	464.7477	927.4809	927.4774	0.0035	48	0.015	R.VNIENPSR.A	Yes
			487.2769	972.5392	972.5392	-0.0001	43	0.0049	R.LQAFEPiR	No
			541.2728	1080.5311	1080.5274	0.0037	45	0.0024	R.EGCQYIAIK.T+ Carbamidomethyl (C)	Yes
			367.8900	1100.6481	1100.6342	0.0139	28	0.024	R.LQAFEPLRK.V	Yes
			610.3830	1218.7515	1218.7448	0.0067	68	3.3e-06	R.VIQPQGLLVPR.Y	Yes
			610.8715	1219.7285	1219.7289	-0.0004	78	3.3e-06	R.VIQPQGLLVPR.Y+ Deamidated (NQ)	Yes
			610.8750	1219.7355	1219.7289	0.0066	36	0.037	R.VIQPQGLLVPR.Y+ Deamidated (NQ)	Yes
			644.3552	1286.6959	1286.6983	-0.0024	87	3.3e-07	R.ALPVDVVANAYR.I	Yes
			644.8314	1287.6483	1287.6823	-0.0340	24	0.019	R.ALPVDVVANAYR.I+ Deamidated (NQ)	Yes
			443.9019	1328.6838	1328.6837	0.0001	53	0.0075	K.TNANAFVSHLAGK.N	Yes
2	GLUB4_ORYSJ	Glutelin type-B 4	487.2769	972.5392	972.5392	-0.0001	43	0.0049	R.LQAFEPiR	No
			538.7890	1075.5634	1075.5298	0.0336	24	0.05	R.GEELGAFTPR.Y	Yes
			610.8715	1219.7285	1219.7289	-0.0004	81	3.3e-07	R.VIEPQGLLVPR.Y	Yes
			611.3611	1220.7076	1220.7129	-0.0053	47	0.0076	R.VIEPQGLLVPR.Y+ Deamidated (NQ)	Yes
			443.8950	1328.6632	1328.6507	0.0125	46	0.032	K.TNANSMVSHLAGK.N	Yes

Family ID (Group)	Accession	Description	Observed	Mr (expt)	Mr (calculated)	Delta	Score	E-value	Match sequence	Unique
			580.9519	1739.8339	1739.8227	0.0112	58	3.5e-05	R.LNIENPSHADTYNPR.A	Yes
2	GLUA2_ORYSJ	Glutelin type-A 2	468.2525	934.4905	934.4872	0.0033	34	0.026	K.TVFNGELR.R	Yes
			487.2769	972.5392	972.5392	-0.0001	43	0.0049	R.LQAFEPiR	No
			529.7542	1057.4938	1057.4902	0.0036	66	9.9e-06	R.EGCAYIAFK.T+ Carbamidomethyl (C)	Yes
			652.3462	1302.6778	1302.6932	-0.0153	71	1.5e-05	R.ALPTDVLANAYR.I	Yes
			452.5570	1354.6491	1354.6663	-0.0172	22	0.02	K.TNPNSMVSHIAGK.S	Yes
2	GLUA3_ORYSJ	Glutelin type-A 3	487.2769	972.5392	972.5392	-0.0001	43	0.0049	R.LQAFEPiR	No
			652.3310	1302.6474	1302.6568	-0.0094	53	0.00074	R.ALPDDVVANAYR.I	Yes
3	CUCIN_ORYSI	Cupincin	463.2769	924.5392	924.5392	-0.0001	61	0.00039	R.VAVLEAAPR.A	Yes
			542.8197	1083.6249	1083.6288	-0.0039	84	9.4e-07	K.EGEGVIVLLR.E	Yes
			675.9083	1349.8020	1349.8031	-0.0011	57	1.6e-05	R.VLAAQPEQILLR.G	Yes
			782.8894	1563.7643	1563.7682	-0.0039	94	7.6e-09	R.FPDEQVVGAAVGGYR.V	Yes
			522.2644	1563.7715	1563.7682	0.0033	58	1.5e-05	R.FPDEQVVGAAVGGYR.V	Yes
			783.3915	1564.7684	1564.7522	0.0162	29	0.0028	R.FPDEQVVGAAVGGYR.V+ Deamidated (NQ)	Yes
4	HSP71_PICAN	Heat shock protein 70 1	592.3318	1182.6491	1182.6397	0.0094	60	0.00089	FELSGIPPAPR.G	No
			678.8228	1355.6311	1355.6180	0.0132	76	1.5e-06	R.FEELCQDLFR.S+ Carbamidomethyl (C)	Yes
			528.5930	1582.7570	1582.7562	0.0008	57	0.00011	R.ARFEELCQDLFR.S+ Carbamidomethyl (C)	Yes
			792.3895	1582.7645	1582.7562	0.0083	78	3e-06	R.ARFEELCQDLFR.S+ Carbamidomethyl (C)	Yes
			830.4452	1658.8758	1658.8879	-0.0121	75	3.5e-05	R.IINEPTAAAiAYGiDK.K	No
			559.2623	1674.7651	1674.7234	0.0417	23	0.0069	K.ATAGDTHLGGEDFDNR.L	No
4	HSP76_HUMAN	Heat shock 70 kDa protein 6	585.8309	1169.6473	1169.6404	0.0069	66	0.00021	K.DAGAIAGiNVLR.I+ Deamidated (NQ)	No
			592.3318	1182.6491	1182.6397	0.0094	60	0.00089	FELSGIPPAPR.G	No
			559.2623	1674.7651	1674.7234	0.0417	23	0.0069	K.ATAGDTHLGGEDFDNR.L	No
			844.4483	1686.8820	1686.8940	-0.0120	86	2.1e-08	R.IINEPTAAAIAYGLDR.R	Yes
			563.3108	1686.9105	1686.8940	0.0164	65	1.5e-06	R.IINEPTAAAIAYGLDR.R	Yes
4	HSP70_LEIAM	Heat shock 70 kDa protein	737.3515	1472.6885	1472.6783	0.0101	78	1.9e-05	R.TTPSYVAFTDSER.L	No
			783.9017	1565.7888	1565.7838	0.0050	65	4.3e-06	K.AVVTVPAYFNDSQR.Q	Yes
			522.9389	1565.7949	1565.7838	0.0111	53	3e-05	K.AVVTVPAYFNDSQR.Q	Yes

Family ID (Group)	Accession	Description	Observed	Mr (expt)	Mr (calculated)	Delta	Score	E-value	Match sequence	Unique
			784.3978	1566.7809	1566.7678	0.0131	46	0.0002	K.AVVTVPAYFNDSQR.Q+ Deamidated (NQ)	Yes
			830.4452	1658.8758	1658.8879	-0.0121	75	3.5e-05	R.IINEPTAAAiAYGiDK.K	No
4	HSP68_DROME	Heat shock protein 68	585.8309	1169.6473	1169.6404	0.0069	66	0.00021	K.DAGAIAGiNVLR.I+ Deamidated (NQ)	No
			737.3515	1472.6885	1472.6783	0.0101	78	1.9e-05	R.TTPSYVAFTDSER.L	No
			830.4452	1658.8758	1658.8879	-0.0121	75	3.5e-05	R.IINEPTAAAiAYGiDK.K	No
4	HSP71_CANAL	Heat shock protein SSA1	592.3318	1182.6491	1182.6397	0.0094	60	0.00089	FELSGIPPAPR.G	No
			600.3398	1198.6650	1198.6670	-0.0019	62	0.00067	K.DAGTIAGLNVLR.I	Yes
			830.4452	1658.8758	1658.8879	-0.0121	75	3.5e-05	R.IINEPTAAAiAYGiDK.K	No
			559.2623	1674.7651	1674.7234	0.0417	23	0.0069	K.ATAGDTHLGGEDFDNR.L	No
4	BIP4_ORYSJ	Heat shock 70 kDa protein BIP4	775.9037	1549.7929	1549.7889	0.0040	55	8.7e-05	R.AVVTVPAYFNDAQR.Q	Yes
			538.6045	1612.7916	1612.7917	-0.0001	55	5.7e-05	R.NDRVEIIANDQGNR.I	Yes
			830.4452	1658.8758	1658.8879	-0.0121	75	3.5e-05	R.IINEPTAAAiAYGiDK.K	No
4	DNAK_DESRM	Chaperone protein DnaK	435.7724	869.5303	869.5334	-0.0031	59	0.00073	K.IAGLEVLR.I	No
			591.8107	1181.6068	1181.6557	-0.0489	19	0.05	R.FQLSGIPPAPR.G	Yes
			592.3318	1182.6491	1182.6397	0.0094	60	0.00089	FELSGIPPAPR.G	No
			830.4452	1658.8758	1658.8879	-0.0121	75	3.5e-05	R.IINEPTAAAiAYGiDK.K	No
4	DNAK_CLOAB	Chaperone protein DnaK	435.7724	869.5303	869.5334	-0.0031	59	0.00073	K.IAGLEVLR.I	No
			451.2788	900.5431	900.5393	0.0039	45	0.033	K.IVLVGGSTR.I	Yes
5	G3P_SCHCO	Glyceraldehyde-3-phosphate dehydrogenase	389.2345	776.4545	776.4545	0.0000	42	0.022	R.LTGLAFR.V	Yes
			410.2233	818.4321	818.4399	-0.0078	51	0.004	K.VGINGFGR.I	Yes
			410.7233	819.4321	819.4239	0.0082	49	0.022	K.VGINGFGR.I+ Deamidated (NQ)	Yes
			428.2561	854.4976	854.4974	0.0002	31	0.042	K.VIPSLNGR.L	Yes
			428.7502	855.4858	855.4814	0.0044	35	0.03	K.VIPSLNGR.L+ Deamidated (NQ)	Yes
			755.9453	1509.8761	1509.8767	-0.0006	86	1.6e-08	R.VPTLDVSVVDLVVR.L	Yes
			504.3055	1509.8948	1509.8767	0.0181	39	0.00086	R.VPTLDVSVVDLVVR.L	Yes
5	G3PC3_ORYSJ	Glyceraldehyde-3-phosphate dehydrogenase 3, cytosolic	561.2971	1120.5796	1120.6128	-0.0332	48	0.00077	K.AGIALSDTFVK.L	Yes
			749.9200	1497.8254	1497.8403	-0.0149	82	6.7e-08	R.VPTVDVSVVDLTVR.L	Yes
6	EF1A_SERIN	Elongation factor 1-alpha	442.7839	883.5532	883.5491	0.0042	59	0.00049	R.QLIVAVNK.M	No

Family ID (Group)	Accession	Description	Observed	Mr (expt)	Mr (calculated)	Delta	Score	E-value	Match sequence	Unique
			457.7808	913.5471	913.5597	-0.0125	68	0.00015	R.QTVAVGVIK.S	Yes
			488.2807	974.5469	974.5437	0.0033	49	0.0099	R.LPLQDVYK.I	No
			513.3095	1024.6045	1024.6030	0.0016	70	4.6e-05	K.IGGIGTVPVGR.V	No
			374.2056	1119.5949	1119.5924	0.0025	30	0.012	K.STTTGHLIYK.C	No
			453.5694	1357.6864	1357.6813	0.0051	70	1.7e-06	K.YMVTVIDAPGHR.D	No
6	EF1A_SCHCO	Elongation factor 1-alpha	442.7839	883.5532	883.5491	0.0042	59	0.00049	R.QLIVAVNK.M	No
			464.7931	927.5717	927.5753	-0.0036	66	0.00016	R.QTVAVGIIK.S	Yes
			488.2807	974.5469	974.5437	0.0033	49	0.0099	R.LPLQDVYK.I	No
			513.3095	1024.6045	1024.6030	0.0016	70	4.6e-05	K.IGGIGTVPVGR.V	No
			374.2056	1119.5949	1119.5924	0.0025	30	0.012	K.STTTGHLIYK.C	No
			453.5694	1357.6864	1357.6813	0.0051	70	1.7e-06	K.YMVTVIDAPGHR.D	No
7	TBB_SCHCO	Tubulin beta chain	563.3470	1124.6795	1124.6706	0.0089	52	8.4e-05	K.LAVNLVPFPR.L	Yes
			573.8063	1145.5981	1145.5829	0.0152	45	0.0059	R.FPGQLNSDLR.K	Yes
			808.4279	1614.8413	1614.8287	0.0126	48	0.0079	R.AILVDLEPGTMDSVR.S	Yes
			816.4152	1630.8159	1630.8236	-0.0077	36	0.0075	R.AILVDLEPGTMDSVR.S+ Oxidation (M)	Yes
			648.3318	1941.9736	1941.9796	-0.0060	59	1.5e-05	K.GHYTEGAELVDAVLDVVR.K	Yes
8	HSP81_ORYSI	Heat shock protein 81-1	541.2796	1080.5446	1080.5240	0.0206	35	0.036	R.APFDLFDTR.K	Yes
			764.4086	1526.8026	1526.7940	0.0086	101	7.9e-08	K.GIVDSEDLPLNISR.E	Yes
8	HSC82_YEAST	ATP-dependent molecular chaperone HSC82	401.2658	800.5170	800.5160	0.0010	43	0.021	R.AILFIPK.R	Yes
			527.2672	1052.5198	1052.5179	0.0020	49	0.016	R.APFDLFESK.K	Yes
9	ACT1_SCHCO	Actin-1	482.7713	963.5281	963.5277	0.0004	51	0.008	R.DLTDFLIK.N	Yes
			571.7790	1141.5435	1141.5404	0.0031	36	0.0026	R.GYPFTTTAER.E	Yes
			400.2393	1197.6961	1197.6982	-0.0022	37	0.0017	R.AVFPSIVGRPR.H	No
			895.9515	1789.8884	1789.8846	0.0038	100	1.6e-07	K.SYELPDGQVITIGNER.F	Yes
			652.0248	1953.0527	1953.0571	-0.0044	42	0.013	R.VAPEEHPVLLTEAPLNPK.A	No
9	ACT1_TOBAC	Actin	400.2393	1197.6961	1197.6982	-0.0022	37	0.0017	R.AVFPSIVGRPR.H	No
			818.9255	1635.8364	1635.8291	0.0074	23	0.035	K.IVLSGGSTMFPGIADR.M+ Oxidation (M)	Yes
			652.0248	1953.0527	1953.0571	-0.0044	42	0.013	R.VAPEEHPVLLTEAPLNPK.A	No

Family ID (Group)	Accession	Description	Observed	Mr (expt)	Mr (calculated)	Delta	Score	E-value	Match sequence	Unique
10	GLB63_ORYSJ	63 kDa globulin-like protein	468.2498	934.4851	934.4872	-0.0021	33	0.026	K.ELAFAASAR.E	Yes
			484.7803	967.5460	967.5451	0.0009	55	0.002	R.VAVLEANPR.S	Yes
			447.2192	1338.6356	1338.6317	0.0040	49	9.6e-05	R.SFHDLAEHDIR.V	Yes
			613.6755	1838.0046	1838.0051	-0.0005	45	0.00019	R.GTVFVVPSGHPIVVTSSR.D	Yes
11	PRO7_ORYSI	Prolamin PPROL 14E	756.9056	1511.7966	1511.7872	0.0095	71	2.1e-07	R.YYGAPSTITTLGGVL.-	Yes
12	IAAS_ORYSJ	Alpha-amylase/subtilisin inhibitor	668.8799	1335.7453	1335.7511	-0.0057	63	4.6e-06	R.VVTGPLIGPSPSGR.E	Yes
13	RS5B_DROME	40S ribosomal protein S5b	492.7823	983.5501	983.5400	0.0101	49	0.0038	R.QAVDVSPLR.R	Yes
			809.4114	1616.8083	1616.8079	0.0004	90	4e-08	K.TVAECLADELINAAK.G+ Carbamidomethyl (C)	Yes
14	BT1_MAIZE	Adenine nucleotide transporter BT1, chloroplastic/amyloplastic/mitochondrial	550.8210	1099.6274	1099.6349	-0.0075	64	0.00043	R.LVSGAIAGAVSR.T	Yes
			573.8295	1145.6445	1145.6445	0.0001	59	0.00081	R.TFVAPLETIR.T	Yes
15	AI171_ORYSJ	17kDa alpha-amylase/trypsin inhibitor 1	516.7796	1031.5446	1031.5434	0.0013	67	4.8e-06	R.CDALSVLVR.G+ Carbamidomethyl (C)	Yes
16	IF4A1_ORYSJ	Eukaryotic initiation factor 4A-1	557.8453	1113.6761	1113.6758	0.0004	55	0.0012	R.VLITTDLLAR.G	Yes
			487.9488	1460.8245	1460.8464	-0.0218	73	1.5e-06	R.ILASGVHVVVGTPGR.V	Yes
17	ALDH4_YEAST	Potassium-activated aldehyde dehydrogenase, mitochondrial	408.7497	815.4848	815.4753	0.0095	49	0.023	K.VTLELGGK.S	No
			534.2799	1066.5452	1066.5407	0.0044	60	0.00014	K.VAFTGSTATGR.H	Yes
17	ALDH_ASPNG	Aldehyde dehydrogenase	408.7497	815.4848	815.4753	0.0095	49	0.023	K.VTLELGGK.S	No
			554.2989	1106.5832	1106.6084	-0.0253	51	0.00064	K.VAFTGSTLVGR.T	Yes
18	RS9A_SCHPO	40S ribosomal protein S9-A	439.7407	877.4668	877.4657	0.0010	46	0.004	K.LAGEYGLR.N	Yes
			516.7969	1031.5793	1031.5764	0.0029	65	0.00036	R.LFEGNAIIR.R	Yes
19	ATPBM_ARATH	ATP synthase subunit beta-1, mitochondrial	488.2859	974.5573	974.5549	0.0023	52	0.0075	K.IGLFGGAGVGK.T	No
			693.3606	1384.7066	1384.7020	0.0046	59	5.8e-05	R.IMNVLGEPIDER.G	Yes
			693.8412	1385.6678	1385.6860	-0.0182	36	0.0015	R.IMNVLGEPIDER.G+ Deamidated (NQ)	Yes
19	ATPB1_PELCD	ATP synthase subunit beta 1	488.2859	974.5573	974.5549	0.0023	52	0.0075	K.IGLFGGAGVGK.T	No
			558.8247	1115.6348	1115.6339	0.0009	50	0.0087	K.VVDLLAPYAR.G	Yes
20	ADT_NEUCR	ADP,ATP carrier protein	618.8367	1235.6588	1235.6510	0.0078	82	5.7e-07	K.TIASDGIAGLYR.G	Yes
21	6PGD1_YEAST	6-phosphogluconate dehydrogenase, decarboxylating 1	557.2659	1112.5172	1112.5040	0.0133	45	0.0019	R.DYFGAHTFR.V	Yes
			796.4106	1590.8067	1590.8002	0.0065	66	1.4e-05	K.GILFVGSGVSGGEEGAR.Y	Yes
22	RS9_DROME	40S ribosomal protein S9	444.7412	887.4678	887.4712	-0.0034	52	0.012	R.IGVLDESR.M	Yes

Family ID (Group)	Accession	Description	Observed	Mr (expt)	Mr (calculated)	Delta	Score	E-value	Match sequence	Unique
			516.7969	1031.5793	1031.5764	0.0029	59	0.0015	R.LFQGNALLR.R+ Deamidated (NQ)	Yes
23	GL81_ORYSJ	Putative germin-like protein 8-1	393.2429	784.4712	784.4694	0.0018	37	0.028	K.IIDLLQA.-	Yes
			446.7351	1782.9112	1782.9166	-0.0053	52	1.8e-05	R.VDYAPLGLNPPHTHPR.A	Yes
			600.6484	1798.9235	1798.9115	0.0120	30	0.0021	R.VDYAPLGLNPPHTHPR.A+ Oxidation (HW)	Yes
24	SCP_CHIOP	Sarcoplasmic calcium-binding protein (Fragment)	421.7589	841.5032	841.5022	0.0011	52	0.0037	-.VATVSLPR.-	Yes
25	RL2_METB6	50S ribosomal protein L2	479.2779	956.5413	956.5403	0.0010	65	0.0004	R.ATVGIVAGGGR.V	Yes
26	G6PD_TAKRU	Glucose-6-phosphate 1-dehydrogenase	630.3121	1258.6097	1258.5983	0.0115	75	3e-06	R.GGYFDDFGIIR.D	Yes
27	SAHH_SCHPO	Adenosylhomocysteinase	559.8078	1117.6011	1117.6132	-0.0121	55	0.0047	K.VAVVAGFGDVGK.G	Yes
			628.8455	1255.6764	1255.6772	-0.0008	48	0.00013	K.VPAINVNDSVTK.S	Yes
28	MDHM_BRANA	Malate dehydrogenase, mitochondrial	610.3553	1218.6960	1218.6972	-0.0012	60	0.00066	K.LFGVTTLDVVR.V	Yes
29	BROM1_ANACO	Fruit bromelain	771.3639	1540.7132	1540.6695	0.0437	65	2e-06	R.NSWGSSWGEGGYVR.M	Yes
30	ATPF_YEAST	ATP synthase subunit 4, mitochondrial	473.2722	944.5298	944.5080	0.0218	52	0.0045	K.AVLDSWVR.Y	Yes
31	MDHM_CAEEL	Probable malate dehydrogenase, mitochondrial	603.3461	1204.6777	1204.6816	-0.0039	64	0.00033	R.VFGVTTLDVVR.S	Yes
32	PGDH_MOUSE	15-hydroxyprostaglandin dehydrogenase [NAD(+)]	592.8466	1183.6785	1183.6925	-0.0139	61	8.5e-05	K.VALVTGAAQGIGK.A	Yes
33	RA05_ORYSJ	Seed allergenic protein RA5	580.9543	1739.8410	1739.8301	0.0109	39	0.00024	R.ELGAPDVGHPMSEVFR.G	Yes
34	ACT17_DICDI	Actin-17	473.2728	944.5311	944.5444	-0.0133	48	0.0013	R.AVFPSIVGR.Q	Yes
35	NLTP1_ORYSI	Non-specific lipid-transfer protein 1	653.7908	1305.5671	1305.5554	0.0117	58	4.5e-05	R.GGAGPSAACCSGVR.S+ 2 Carbamidomethyl (C)	Yes
36	FTSI4_ARATH	Probable inactive ATP-dependent zinc metalloprotease FTSHI 4, chloroplastic	569.8482	1137.6818	1137.6757	0.0060	57	0.00049	R.LDILDPALLR.K	Yes
37	GUAA_HELAH	GMP synthase [glutamine-hydrolyzing]	551.7986	1101.5827	1101.5778	0.0049	57	0.00077	R.ITNEVSGINR.V	Yes
38	ENO_ALTAL	Enolase	755.4231	1508.8316	1508.8562	-0.0246	57	4.8e-06	K.AVANVNDTIAPALIK.E	Yes
39	GLNA_SUIBO	Glutamine synthetase	396.8755	1187.6048	1187.6047	0.0001	37	0.0083	R.RPASNIDPYR.V	Yes
			839.4120	1676.8095	1676.8158	-0.0064	43	0.00065	K.VQAEYVWIDGDGGLR.S	Yes
40	YADA_YERPS	Adhesin YadA	502.2857	1002.5568	1002.5709	-0.0141	41	0.0025	K.KSAEALVSAK.V	Yes
41	PRDX_ASCSU	Peroxiredoxin	606.3391	1210.6637	1210.6670	-0.0033	55	0.0023	R.QITVNDLPVGR.S	Yes
42	NDK_BACHD	Nucleoside diphosphate kinase	474.2665	1419.7776	1419.7544	0.0232	55	0.0024	R.TYLMIKPDGVQR.N	Yes
43	RL5_CUCSA	60S ribosomal protein L5	485.3088	968.6031	968.6019	0.0012	55	0.00098	R.ALLDVGLIR.T	Yes
44	DHSO_ARATH	Sorbitol dehydrogenase	644.3426	1286.6707	1286.6441	0.0266	53	0.0015	R.VALEPGISCWR.C+ Carbamidomethyl (C)	Yes
45	RL4A_SCHPO	60S ribosomal protein L4-A	605.3505	1208.6865	1208.6877	-0.0012	45	0.0013	R.NIPGVEIVNVR.R	Yes

Family ID (Group)	Accession	Description	Observed	Mr (expt)	Mr (calculated)	Delta	Score	E-value	Match sequence	Unique
46	Y3511_METJA	Uncharacterized protein MJECL11	458.7551	915.4957	915.5025	-0.0069	51	0.014	R.ETLGIDLR.G	Yes
47	DNAK_GRATL	Chaperone protein dnaK	614.3159	1226.6173	1226.6619	-0.0446	50	0.0004	K.DAGQIAGLDVLR.I	Yes
48	Y2672_THEFY	UPF0234 protein Tfu_2672	382.2242	762.4338	762.4276	0.0062	51	0.013	K.AALDVFK.S	Yes
49	EF2_BOVIN	Elongation factor 2	546.2974	1090.5803	1090.5771	0.0032	56	0.00027	M.VNFTVDQIR.A	Yes
50	RL13_CANAL	60S ribosomal protein L13	439.7507	877.4868	877.4909	-0.0041	50	0.0014	R.GFTLAELK.A	Yes
51	GLGL1_MAIZE	Glucose-1-phosphate adenylyltransferase large subunit 1, chloroplastic/amyloplastic	624.3126	1246.6107	1246.6129	-0.0021	50	0.00021	R.ATPAVPVGGCYR.L+ Carbamidomethyl (C)	Yes
52	NLTP2_MAIZE	Probable non-specific lipid-transfer protein 2	528.7619	1055.5093	1055.5036	0.0057	50	0.00028	R.SPNYGSYIR.S	Yes
53	PPDK2_ORYSJ	Pyruvate, phosphate dikinase 2	498.7614	995.5081	995.5076	0.0005	49	0.0038	R.LDFEGIFR.A	Yes
54	SBNO2_MOUSE	Protein strawberry notch homolog 2	514.7965	1027.5784	1027.6026	-0.0242	49	0.014	R.LSLEAAGVIR.I	Yes
55	PGK_MYCBO	Phosphoglycerate kinase	408.7345	815.4544	815.4389	0.0156	48	0.03	K.ALLDADAK.V	Yes
56	ARTA_ASPFN	14-3-3 family protein artA	454.2690	906.5234	906.5174	0.0060	48	0.011	R.NLLSVAYK.N	Yes
57	KTHY_CHLPM	Thymidylate kinase	422.2670	842.5194	842.5225	-0.0031	48	0.018	R.LILLESR.H	Yes
58	AMPA_GEOTN	Probable cytosol aminopeptidase	479.7696	957.5246	957.5243	0.0003	47	0.028	R.LGGQLSNLR.K+ Deamidated (NQ)	Yes
59	PSAM_PROMP	Photosystem I reaction center subunit XII	365.2364	728.4581	728.4545	0.0037	47	0.026	R.LGISLAR.T	Yes
60	TBAB_SCHCO	Tubulin alpha-1B chain	750.9022	1499.7898	1499.7845	0.0053	47	0.00034	K.NLGVAQPGFTNLNR.L	Yes
61	IPYR_ASHGO	Inorganic pyrophosphatase	462.2208	922.4271	922.4297	-0.0026	47	0.025	R.ATNEWFR.I	Yes
62	RL15_FRAP2	50S ribosomal protein L15	480.2718	958.5291	958.5335	-0.0044	47	0.0091	K.IIASGEIQK.A+ Deamidated (NQ)	Yes
63	AMYG_SCHCM	Glucoamylase ARB_02327-1	443.2263	884.4380	884.4392	-0.0012	35	0.003	K.VYVDSFR.G	Yes
64	FUSIL_DROME	RNA-binding protein fusilli	378.7428	755.4711	755.4654	0.0058	46	0.012	K.GAQVIIR.M	Yes
65	ARGJ_ACIAD	Arginine biosynthesis bifunctional protein ArgJ	443.2784	884.5423	884.5331	0.0092	46	0.018	K.ILEILQR.I+ Deamidated (NQ)	Yes
66	SAM11_HUMAN	Sterile alpha motif domain-containing protein 11	493.2902	984.5659	984.5465	0.0194	45	0.03	R.ISSPVNRGR.L	Yes
67	YIGA_SALTY	Uncharacterized protein YigA	430.7364	859.4582	859.4552	0.0031	45	0.0096	R.QAFEPLR.I	Yes
68	GYRA_CLOAB	DNA gyrase subunit A	489.2628	976.5111	976.4978	0.0134	45	0.039	R.LAQDFNLR.Y+ Deamidated (NQ)	Yes
69	HEAT1_HUMAN	HEAT repeat-containing protein 1	523.2930	1044.5715	1044.5564	0.0152	45	0.0064	M.TSLAQQLQR.L+ Deamidated (NQ)	Yes
70	Y7732_DICDI	FNIP repeat-containing protein DDB_G0277323	350.7160	699.4174	699.4167	0.0007	43	0.014	K.IINILN.-+ Deamidated (NQ)	Yes
71	Y1823_CLOD6	UPF0246 protein CD630_18230	464.7738	927.5330	927.5501	-0.0171	44	0.038	K.LAVLNKNR.F+ Deamidated (NQ)	Yes
72	EIF3F_ASPCL	Eukaryotic translation initiation factor 3 subunit F	465.2885	928.5624	928.5706	-0.0081	44	0.035	R.VIGTLLGTR.S	Yes
73	RA16_ORYSJ	Alpha-amylase/trypsin inhibitor RA16	653.8184	1305.6223	1305.6136	0.0087	44	0.00058	R.QLAAIDSSWCR.C+ Carbamidomethyl (C)	Yes

Family ID (Group)	Accession	Description	Observed	Mr (expt)	Mr (calculated)	Delta	Score	E-value	Match sequence	Unique
74	NUR1_YEAS1	Nuclear rim protein 1	462.7470	923.4795	923.4865	-0.0070	38	0.0086	R.FLNNLFR.F+ Deamidated (NQ)	Yes
75	PP1_EMENI	Serine/threonine-protein phosphatase PP1	684.3505	1366.6864	1366.6882	-0.0018	44	0.0023	R.GVSFTFGPDVVSR.F	Yes
76	MURA_ALKEH	UDP-N-acetylglucosamine 1-carboxyvinyltransferase	556.8159	1111.6173	1111.6462	-0.0289	43	0.0065	K.LAQLGADIRR.V	Yes
77	KINH_BOTFU	Kinesin heavy chain	587.3117	1172.6089	1172.6149	-0.0061	43	0.011	R.ILQESLGGNSR.T	Yes
78	HTPG_BAUCH	Chaperone protein HtpG	639.6756	1916.0050	1916.0190	-0.0139	34	0.0021	R.FVKGIIDCNDLPLNISR.E	Yes
79	YKT6_ASHGO	Synaptobrevin homolog YKT6	473.2579	944.5012	944.5291	-0.0279	42	0.016	K.TIESVLQR.G	Yes
80	PPSA_NEIMB	Phosphoenolpyruvate synthase	508.7900	1015.5655	1015.5550	0.0105	42	0.038	R.TLGEAEAVVK.A	Yes
81	RSSA_AJECG	40S ribosomal protein S0	463.7896	925.5647	925.5597	0.0050	42	0.026	R.LIIVTDPR.T	Yes
82	COBRA_ARATH	Protein COBRA	571.7945	1141.5745	1141.5768	-0.0022	35	0.014	K.ITITNFNYR.L+ Deamidated (NQ)	Yes
83	CYPH_SCHPO	Peptidyl-prolyl cis-trans isomerase	466.7633	931.5121	931.5015	0.0106	39	0.029	K.LFDDVVPK.T	Yes
84	SEPT2_DROME	Septin-2	483.8200	965.6255	965.6273	-0.0018	38	0.012	K.VNIIPVIAK.A	Yes
85	RS3_AMBME	40S ribosomal protein S3	399.7588	797.5031	797.5123	-0.0092	39	0.045	K.LLGGLAVR.R	Yes
86	MURD_COXBN	UDP-N-acetylmuramoylalanine--D-glutamate ligase	578.3270	1154.6395	1154.6659	-0.0264	39	0.012	K.GQLILIAGGQGK.G+ Deamidated (NQ)	Yes
87	UBIQ_AVESA	Ubiquitin	356.5418	1066.6035	1066.6135	-0.0099	33	0.0031	K.ESTLHLVLR.L	Yes
88	FBL63_ARATH	F-box/LRR-repeat protein At3g59190	760.4001	1518.7856	1518.8042	-0.0186	38	0.0019	K.VNFDSLVEASIGLR.M	Yes
89	AEP1_YEAS1	ATPase expression protein 1, mitochondrial	468.2580	934.5015	934.5123	-0.0108	38	0.043	K.YQLQIIR.A+ 2 Deamidated (NQ)	Yes
90	TREPH_GRIFR	Trehalose phosphorylase	602.8201	1203.6256	1203.6248	0.0009	33	0.0014	K.GIPNVIDSYAR.F	Yes
			603.3142	1204.6138	1204.6088	0.0050	20	0.019	K.GIPNVIDSYAR.F+ Deamidated (NQ)	Yes
			419.2576	1254.7510	1254.7448	0.0062	15	0.038	R.IRPDLPIIYR.S	Yes
91	RHOA_EMENI	GTP-binding protein rhoA	673.3282	2016.9627	2016.9177	0.0449	37	0.00042	K.HVELALWDTAGQEDYDR.L	Yes
92	PPDK_FLABI	Pyruvate, phosphate dikinase, chloroplastic	485.7437	969.4728	969.4556	0.0172	36	0.0099	R.SDFEGIFR.A	Yes
93	SAHHA_XENLA	Adenosylhomocysteinase A	511.3068	1020.5991	1020.6080	-0.0089	36	0.019	R.HIILLAEGR.L	Yes
94	CDC48_EMENI	Cell division control protein 48	634.8182	1267.6219	1267.6408	-0.0190	36	0.011	R.EVDIGIPDPTGR.L	Yes
95	ACKA_STAEQ	Acetate kinase	482.2814	962.5482	962.5437	0.0046	36	0.028	R.VALDLFASK.I	Yes
96	RISB_ACTPJ	6,7-dimethyl-8-ribityllumazine synthase	506.7330	1011.4515	1011.4662	-0.0147	35	0.049	R.FNDFINDK.L	Yes
97	PRO25_ORYSJ	13 kDa prolamin C	526.7745	1051.5345	1051.5339	0.0006	34	0.0031	R.SIATVGGVWY.-	Yes
98	RS2_COREF	30S ribosomal protein S2	510.2829	1018.5512	1018.5593	-0.0081	33	0.05	K.RETLMLTR.E	Yes
99	PALC_CRYNB	pH-response regulator protein palC	428.7642	855.5138	855.4854	0.0284	33	0.018	K.AFIPPPSK.F	Yes

Family ID (Group)	Accession	Description	Observed	Mr (expt)	Mr (calculated)	Delta	Score	E-value	Match sequence	Unique
100	K2C1B_HUMAN	Keratin, type II cytoskeletal 1b	590.3035	1178.5924	1178.5931	-0.0007	32	0.017	K.YQELQITAGR.H+ Deamidated (NQ)	Yes
101	TAW22_PYRAB	tRNA (guanine(37)-N1)/4-demethylwyosine(37)-methyltransferase Taw22	390.7035	779.3925	779.3966	-0.0042	32	0.035	K.VFFNPR.M+ Deamidated (NQ)	Yes
102	MURF_BUCBP	UDP-N-acetylmuramoyl-tripeptide--D-alanyl-D-alanine ligase	466.7609	931.5072	931.5412	-0.0340	32	0.038	-.MISINLIK.L+ Deamidated (NQ)	Yes
103	ANK3_HUMAN	Ankyrin-3	478.2606	954.5066	954.4771	0.0295	32	0.03	K.SAVSPDVHK.S+ Oxidation (HW)	Yes
104	IAAT_ELECO	Alpha-amylase/trypsin inhibitor	585.3225	1168.6304	1168.5910	0.0394	32	0.0078	R.LLQDLPGCPR.Q+ Carbamidomethyl (C); Deamidated (NQ)	Yes
105	MIAA_GRAFK	tRNA dimethylallyltransferase	591.3186	1180.6225	1180.5910	0.0315	31	0.042	R.EMIYDRINK.R	Yes
106	BGH3A_BACO1	Beta-glucosidase BoGH3A	680.3359	1358.6572	1358.6751	-0.0179	31	0.0062	R.EGKIPMEVVNDK.A+ Deamidated (NQ)	Yes
107	UBC1_COLGL	Ubiquitin-conjugating enzyme E2-16 kDa	623.3352	1244.6559	1244.6401	0.0158	31	0.0019	R.DQWSPALTISK.V	Yes
108	RECF_PROMH	DNA replication and repair protein RecF	596.3430	1190.6714	1190.6295	0.0419	30	0.03	K.VLSYTSLGAHK.A+ Oxidation (HW)	Yes
109	RPOB_WOLPI	DNA-directed RNA polymerase subunit beta	425.2332	848.4518	848.4433	0.0085	29	0.014	K.VPFFPDK.F	Yes
110	CYC_CANAL	Cytochrome c	384.8800	1151.6183	1151.6200	-0.0017	28	0.013	K.VGPNLHGVFGR.K	Yes
111	PYRD2_BOVIN	Pyridine nucleotide-disulfide oxidoreductase domain-containing protein 2	584.8302	1167.6459	1167.6135	0.0323	28	0.047	R.DILTPPDLER.V	Yes
112	RPA43_YEAST	DNA-directed RNA polymerase I subunit RPA43	637.3200	1272.6254	1272.6561	-0.0307	28	0.012	R.SQAESLPIVSNK.K+ Deamidated (NQ)	Yes
113	GLYA_LACRD	Serine hydroxymethyltransferase	752.3921	1502.7696	1502.7576	0.0120	28	0.026	K.TGLTGKDAQNLLDR.V+ 2 Deamidated (NQ)	Yes
114	MED17_BOVIN	Mediator of RNA polymerase II transcription subunit 17	420.2289	1257.6649	1257.6751	-0.0102	27	0.014	K.LEAAQNVLLCK.E+ Carbamidomethyl (C)	Yes
115	VATA_NEUCR	V-type proton ATPase catalytic subunit A	441.5748	1321.7026	1321.6990	0.0036	26	0.033	K.VGHDQLVGEVIR.I+ Deamidated (NQ)	Yes
116	P2P_LACPA	PII-type proteinase	540.2695	1078.5245	1078.5295	-0.0050	26	0.045	K.NAVTFDQGVK.F+ Deamidated (NQ)	Yes
117	CIAO1_ASPTN	Probable cytosolic iron-sulfur protein assembly protein 1	493.7654	985.5162	985.4869	0.0294	26	0.041	R.VYSLSNFR.L+ Deamidated (NQ)	Yes
118	TOL4_ARATH	TOM1-like protein 4	602.8118	1203.6091	1203.5983	0.0108	25	0.039	R.NLSLTSSEPQK.E+ Deamidated (NQ)	Yes
119	Y2148_CALS8	UPF0102 protein Csac_2148	753.3791	1504.7437	1504.7660	-0.0223	25	0.015	K.NEVLDINLIKNAF.-+ 3 Deamidated (NQ)	Yes
120	PGK_DROME	Phosphoglycerate kinase	623.3230	1244.6314	1244.6765	-0.0450	25	0.029	R.VDFNVPIKEGK.I	Yes
121	MURG_SALAR	UDP-N-acetylglucosamine--N-acetylmuramyl-(pentapeptide) pyrophosphoryl-undecaprenol N-acetylglucosamine transferase	655.8818	1309.7490	1309.7354	0.0136	24	0.033	R.VLNQTLPQVAAR.L+ Deamidated (NQ)	Yes
122	RL32_METM6	50S ribosomal protein L32e	561.2912	1120.5679	1120.5764	-0.0085	23	0.047	K.QKQEEFLSL.-	Yes
123	CB016_HUMAN	Uncharacterized protein C2orf16	628.3385	1254.6625	1254.6682	-0.0057	22	0.025	K.FMQLLFQSLK.R+ Deamidated (NQ)	Yes
124	R23A1_ARATH	60S ribosomal protein L23a-1	405.2253	1212.6540	1212.6826	-0.0286	22	0.031	K.VNTLIRPDGTK.K	Yes
125	G3P2_MUCCL	Glyceraldehyde-3-phosphate dehydrogenase 2	427.5645	1279.6718	1279.6231	0.0487	21	0.012	-.MVTQVGINGFGR.I+ 2 Deamidated (NQ)	Yes

Family ID (Group)	Accession	Description	Observed	Mr (expt)	Mr (calculated)	Delta	Score	E-value	Match sequence	Unique
126	CLIP1_CHICK	CAP-Gly domain-containing linker protein 1	1077.5462	1077.5462	1077.5189	0.0273	20	0.014	K.NEEELTKSK.E+ Deamidated (NQ)	Yes
127	RSMH_DECAR	Ribosomal RNA small subunit methyltransferase H	837.4176	1672.8206	1672.8169	0.0037	19	0.04	R.EPGQDPATRSFQALR.I+ Deamidated (NQ)	Yes
128	MSP1_GLORO	Major sperm protein 1	481.7536	961.4926	961.4756	0.0170	18	0.031	K.NLPIEYNV.-+ Deamidated (NQ)	Yes
129	RLMN_PROMP	Probable dual-specificity RNA methyltransferase RlmN	575.7787	1149.5429	1149.5886	-0.0457	18	0.046	-.MKNLLGCSVK.D+ Carbamidomethyl (C); Deamidated (NQ)	Yes
130	PTOV1_BOVIN	Prostate tumor-overexpressed gene 1 protein homolog	727.9135	1453.8124	1453.8001	0.0123	16	0.035	R.RVIANQQQVLQR.N+ 2 Deamidated (NQ)	Yes
131	NFI_PETMO	Endonuclease V	791.4115	1580.8085	1580.8120	-0.0035	15	0.047	K.DEGLAVIVTMDFKK.L+ Oxidation (M)	Yes
132	XERS_STRSY	Tyrosine recombinase XerS	852.9833	1703.9521	1703.9821	-0.0300	15	0.04	R.IADIPLDVLEHLTKK.D	Yes
133	PUR7_PROMP	Phosphoribosylaminoimidazole-succinocarboxamide synthase	763.3778	1524.7411	1524.7494	-0.0082	15	0.042	K.FEGKGELNCLISSK.I+ Deamidated (NQ)	Yes
